# Copper(II)-Catalyzed (3+2) Cycloaddition of 2*H*-Azirines to Six-Membered Cyclic Enols as a Route to Pyrrolo[3,2-*c*]quinolone, Chromeno[3,4-*b*]pyrrole, and Naphtho[1,8-*ef*]indole Scaffolds

**DOI:** 10.3390/molecules27175681

**Published:** 2022-09-02

**Authors:** Pavel A. Sakharov, Nikolai V. Rostovskii, Alexander F. Khlebnikov, Mikhail S. Novikov

**Affiliations:** Institute of Chemistry, St. Petersburg State University, 7/9 Universitetskaya Nab., 199034 St. Petersburg, Russia

**Keywords:** azirines, pyrrolines, cycloaddition, annulation, copper catalysis

## Abstract

A method for the [2+3] pyrroline annulation to the six-membered non-aromatic enols using 3-aryl-2*H*-azirines as annulation agents is developed in the current study. The reaction proceeds as a formal (3+2) cycloaddition via the N1-C2 azirine bond cleavage and is catalyzed by both Cu(II) and Cu(I) compounds. The new annulation method can be applied to prepare pyrrolo[3,2-*c*]quinoline, chromeno[3,4-*b*]pyrrole, and naphtho[1,8-*ef*]indole derivatives in good to excellent yields from enols of the quinolin-2-one, 2*H*-chromen-2-one, and 1*H*-phenalen-1-one series.

## 1. Introduction

**Scheme 1:**
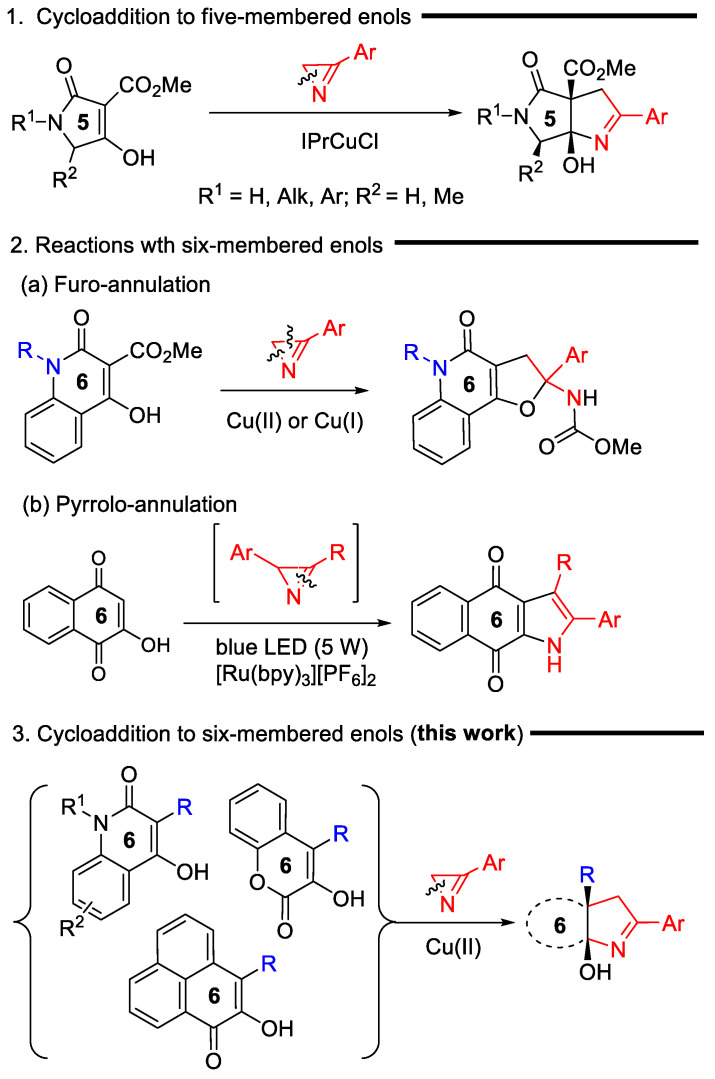
Reactions of cyclic enols with 2*H*-azirines.

2*H*-Azirines are widely used for the preparation of various 4–7-membered N-, N,N-, and N,O-heterocycles of varying degrees of unsaturation and different heteroatom arrangements [1,2]. The ability of azirines to open at any of the three bonds of the ring under the action of electrophilic and nucleophilic reagents, as well as transition metal compounds, underlies a powerful strategy for the synthesis of azete [3], pyrrole [4,5,6], oxazole [7,8,9], imidazole [10,11,12], 1,2,3-triazole [13], pyridine [14] derivatives, and other heterocycles, which are hardly accessible with conventional methods. Some of the azirine-ring opening reactions can be applied for the synthesis of *ortho*-fused, spiro-fused, and bridged heterocycles. These heteropolycycles can be the result of both intramolecular and intermolecular reactions of azirines [1]. Among them, intermolecular cycloaddition reactions are of particular interest, since they satisfy the requirements of green chemistry being atom-economical processes. In contrast to the (2+3)- and (2+4)-cycloaddition reactions of azirines with 1,3-dipoles and 1,3-dienes (or their aza-analogs) [15,16,17,18,19], in which azirines, without the ring opening, provide the incorporation of a diatomic N–C fragment in the resulting heterocycle, the reaction sequence “azirine-ring opening/cycloaddition” provides the incorporation of all atoms of the azirine ring into a new heterocyclic system. This annulation strategy includes transition-metal-catalyzed reactions of azirines with cyclic diazo compounds [20,21,22], Y(OTf)_3_-catalyzed [3+6] cycloaddition of azirines to fulvenes [23] leading to 3,4-dihydro-2*H*-cyclopenta[*c*]pyridine derivatives, photoinduced (3+2) cycloaddition of nitrile ylides, generated via the azirine-ring opening (C2–C3 azirine bond cleavage), to quinones [24] or *N*-benzylmaleimide [25], synthesis of cycloalkane-fused pyrroles by the Fe(III)-catalyzed decarboxylative (3+2) cycloaddition of the 2*H*-azirines to cyclic β-ketoacids (N1–C3 azirine bond cleavage) [26], and synthesis of pyrrolo[3,4-*b*]pyrrole derivatives via Cu(I)-catalyzed (3+2) cycloaddition of azirines to the enol carbon–carbon double bond of 3-methoxycarbonyl-substituted tetramic acids (Scheme 1, reaction 1) [27]. The last of these methods can be effectively applied to the pyrroline annulation of tetronic and thiotetronic acids as well [28]. However, attempts to extend this method to six-membered enols of the quinoline-3-carboxylate series unexpectedly encountered a serious problem associated with the involvement of the ester substituent in the transformation (Scheme 1, reaction 2a). This reaction also proceeds through azirine N1-C2 bond cleavage under both Cu(I) and Cu(II) catalysis, but exclusively produces the furo-annulation product [29]. A visible-light-promoted (3+2) cycloaddition of azirines, derived in situ from vinyl azides, to α-hydroxybenzoquinones is the only successful example of azirine cycloaddition to a multiple bond of a six-membered cyclic enol to date (Scheme 1, reaction 2b) [30]. However, this reaction cannot serve as an alternative to the method of copper-catalyzed annulation of enols with azirines, since it proceeds via the cleavage of not a single N-C2 azirine bond, but a multiple N-C3 bond, and provides a pyrrole ring with another substitution pattern. Thus, the search for the effective conditions for (3+2) cycloadditions of azirines to unsaturated cyclic systems, including cyclic enols, the elucidation of mechanisms of these reactions, assessing their scope and limitations still remains an outstanding challenge.

In this study, we describe a method for the pyrroline annulation of six-membered non-aromatic enols of the quinolin-2-one, 2*H*-chromen-2-one, and 1*H*-phenalen-1-one series. Additionally, a reaction mechanism is presented that allows us to define the scope of this method.

## 2. Results and Discussion

**Scheme 2:**
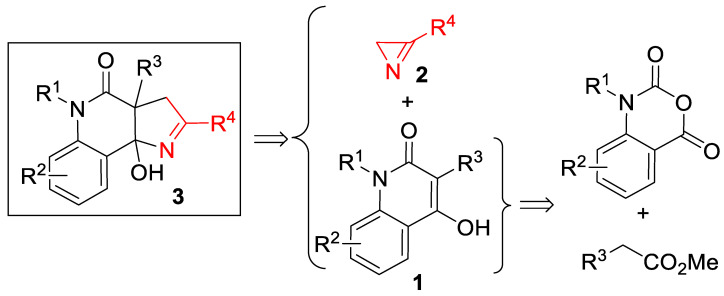
Retrosynthetic scheme for 1*H*-pyrrolo[3,2-*c*]quinolones **3**.

Our initial interest in (3+2)-cycloaddition reactions of azirines **2** was related to their possible use for the rapid assembly of 1*H*-pyrrolo[3,2-*c*]quinoline framework **3** from 4-hydroxyquinolone derivatives **1** (Scheme 2). The structural motif of 1*H*-pyrrolo[3,2-*c*]quinoline has always attracted the attention of synthetic chemists [31,32], as it is included in many bioactive, natural products [33,34,35,36] and synthetic compounds that possess enzyme modulator [37], antitumor [38], and 5-hydroxytryptamine(6) receptor antagonist activities [39]. The attractiveness of the mentioned approach to these compounds lies in the easy availability of quinolones **1**, which can be prepared from isatoic anhydrides. It should also be noted that the synthesis of pyrroline-fused systems bearing a bridgehead hydroxy group is a challenge, since conventional annulation methods either produce unsatisfactory results [40] or require the use of an aggressive medium, such as liquid ammonia [41].

In our initial experiments, we synthesized 3-(4-methoxyphenyl)-substituted quinolin-2-one **1a** from *N*-methylisatoic anhydride and methyl 4-methoxyphenylacetate according to the procedure described in the literature [42]. Enol **1a** turned out to be inactive toward azirine **2a** when heated at 100 °C in methanol, toluene, or 1,2-dichloroethane (DCE). At higher temperatures, the formation of azirine decomposition products without enol involvement was observed. The reaction between **1a** and **2a** commenced at 100 °C in methanol when catalytic amounts of the Cu(I)-NHC complex IPrCuCl (5 mol%) were added, and resulted in the formation of the desired annulation product, pyrroloquinolone **3a**, in 98% yield in 20 min (Table 1, entry 2). It was notable that a close to quantitative yield of **3a** was also achieved with all tested copper(II) catalysts (entries 3–5). The optimal ratio **1a**/**2a** was observed to be 1:1.6, whereas the reaction between equimolar amounts of the reagents provided only 65% yield (entry 6). Replacing anhydrous methanol with 96% aqueous ethanol led to a decrease in the yield (entry 7). A high yield of **3a** in the CuCl_2_-catalyzed reaction was obtained by increasing the amount of the azirine to 2 equiv. To our surprise, cobalt(II) acetate as well as nickel(II) and iron(III) acetylacetonates also catalyzed the reaction, albeit with less efficiency (entries 9–11). As a result, we used the 1:1.6 mixture of enol **1** and azirine **2** in the presence of copper(II) acetate monohydrate (5 mol%) as a catalyst at 100 °C in methanol as optimal conditions for the further experiments.

**Scheme 3:**
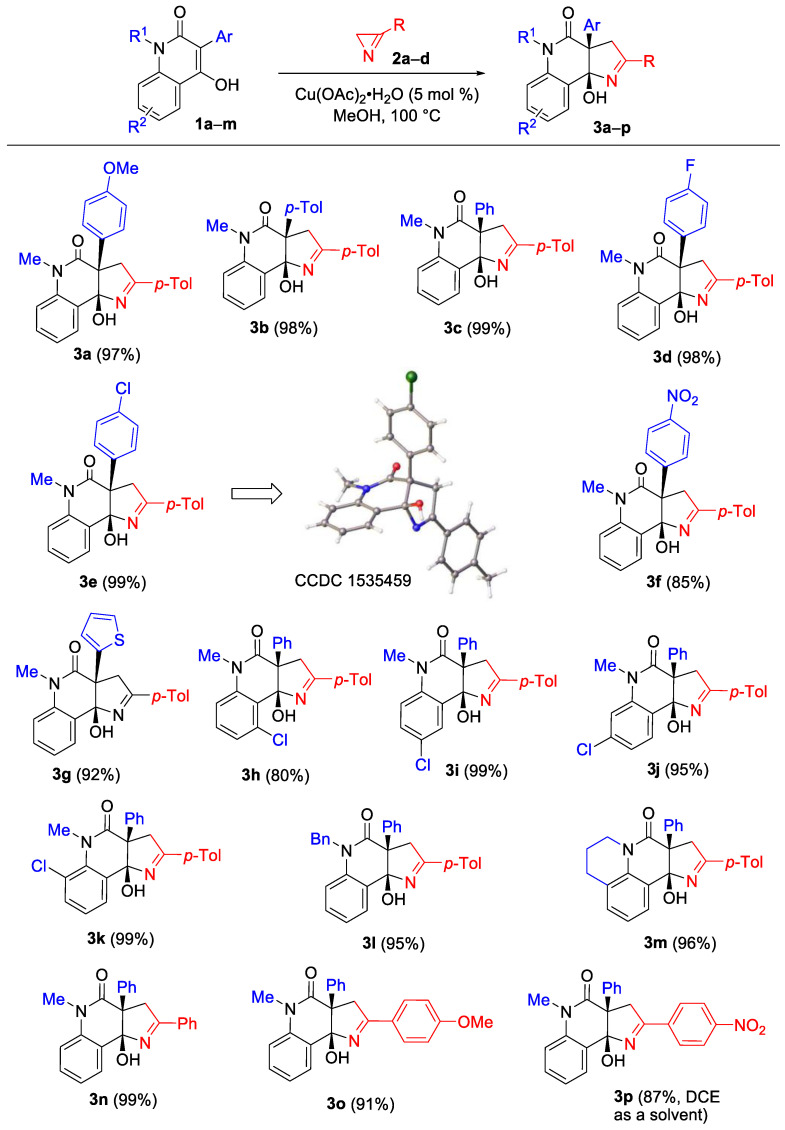
Synthesis of pyrroloquinolinones **3**.

The scope of hydroxyquinolones **1** was then evaluated under the optimized conditions using 3-(*p*-tolyl)-2*H*-azirine (**2a**) as the reaction partner (Scheme 3). The reaction displayed a low sensitivity to the electronic effects of the aryl substituent at C3 of the enol (compounds **3a**–**f**). In addition, the annulation product **3g** was synthesized from a quinolone with a 2-thienyl substituent at the C3 in 92% yield. The reaction was observed to be tolerant to the presence of a substituent at any position of the benzene ring of the quinolone moiety and provided high product yields (compounds **3h**–**k**). Additional *ortho*- and *peri*-fusion (as in quinolone **1m**) also did not influence the product yield (compound **3m**).

The structures of compounds **3a**–**p** were established using NMR spectroscopy and HRMS methods. The structure of compound **3e** was additionally verified by X-ray diffraction analysis.

**Scheme 4:**
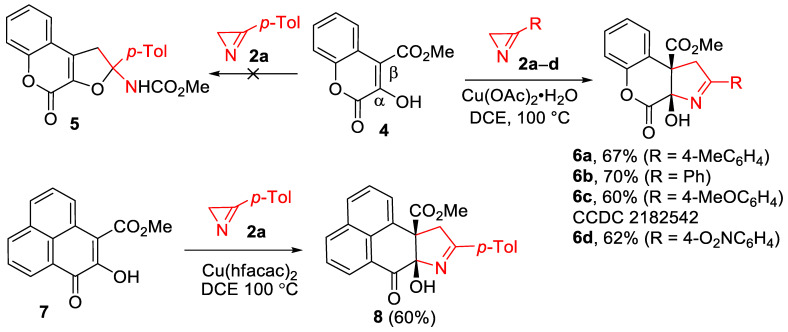
Synthesis of chromenopyrrolones **6** and adduct **8**.

The comparison of the obtained results with the data of our previous work [29] (Scheme 1, reaction 2a) revealed a dramatic dependence of the reaction outcome on the nature of the C3 substituent in quinolones **1**: 3-alkoxycarbonyl-substituted derivatives produced the products of furo-annulation, while 3-aryl-substituted derivatives exclusively produced pyrroline-fused products **3**. In order to find out how general this pattern was for six-membered enols, we examined compounds **4** and **7**, having carbonyl substituents both at the α- and β-carbon atoms of the enol moiety (Scheme 4). The Cu(OAc)_2_- and IPrCuCl-catalyzed reactions of chromenone **4** with azirine **2a**, conducted in methanol at 100 °C, resulted in a complex, inseparable mixture of products. IPrCuCl did not catalyzed the reaction in DCE at all, but, to our delight, the target chromenopyrrole **6a** was obtained in 67% yield in DCE using Cu(OAc)_2_ × H_2_O (5 mol%) as a catalyst. According to the ^1^H NMR spectrum of the reaction mixture, no traces of the furo-annulated product, compound **5**, were detected. The reaction of chromenone **4** with azirines **2b**–**d** occurred with almost the same efficiency, producing chromenopyrroles **6b**–**d** in 60–70% yields. The structure of compound **6c** was confirmed by X-ray diffraction analysis.

Phenalenone **7** with a similarly substituted enol moiety included in the *ortho*- and *peri*-fused system also reacted well with azirine **2a**, producing the tetracyclic annulation adduct **8** in a 60% yield. In this reaction, copper(II) hexafluoroacetylacetonate (10 mol%) was used as a catalyst since it provided a slightly higher product yield than copper(II) acetate.

**Scheme 5:**
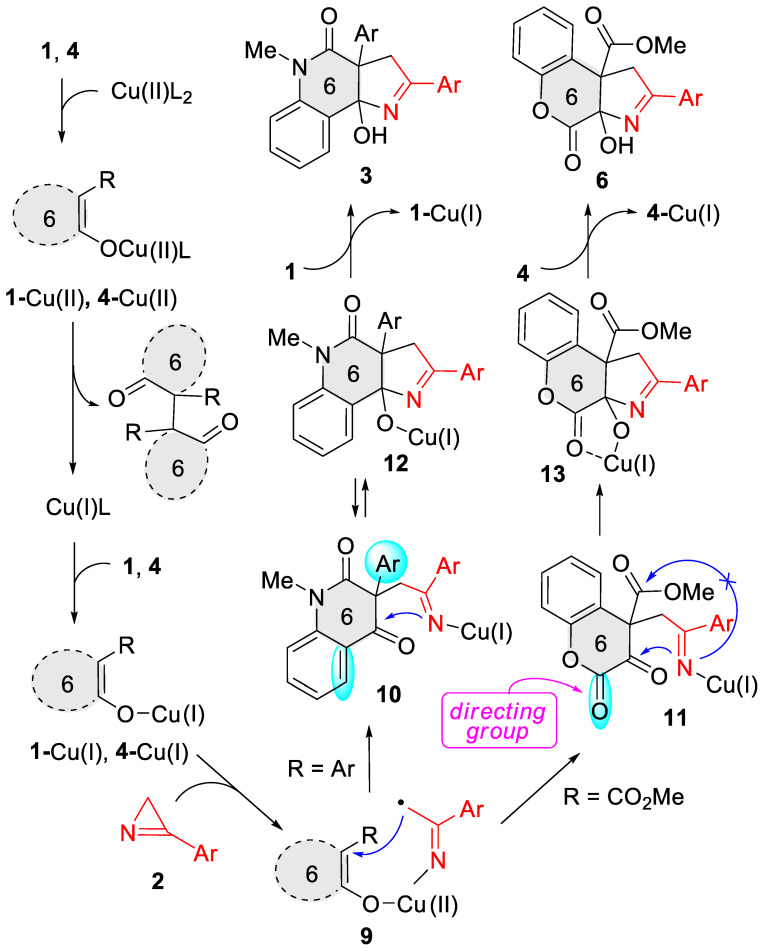
Plausible mechanism.

Thus, the substitution pattern of the enol moiety of six-membered cyclic enols controlled the outcome of their catalytic annulation with azirines directing the reaction toward either pyrroline- or furane-fused products. In the presence of an aryl substituent at the β-position of the enol moiety of quinolones **1**, its (2+3) cycloaddition to azirines **2** smoothly proceeded to afford pyrroline-annulated products **3**. In contrast, the CO_2_Me group in the same position directs the process toward the formation of furo-annulated products as follows from the results of the previous studies [29]. However, this switching does not occur if the α-carbon of the enol moiety is adjacent to the endocyclic carbonyl carbon atom. We proposed the reaction mechanism (Scheme 5) to address the observed reactivity of non-aromatic six-membered cyclic enols toward azirines under copper catalysis. The key step of the reaction was the azirine-ring opening across the N1-C2 bond to form radical **9** under the action of copper(I) enolate **1**-Cu(I)/**4**-Cu(I). The latter resulted from the oxidative homocoupling of the enol with the copper(II) catalyst. Such an oxidation with copper(II) acetylacetonate was previously reported for tetramic acids [27]. Indeed, enol **1a** reacts with Cu(OAc)_2_ in boiling MeOH, but, unfortunately, our attempts to isolate the oxidative coupling product failed because of the low selectivity of the reaction, which yielded an inseparable mixture of products. Intramolecular radical attack in intermediate **9** afforded copper(I) iminide **10**,**11**. 3-Aryl-substituted iminide **10** underwent the cyclization at the keto group followed by a copper–hydrogen exchange to produce cycloadduct **3**. The expected intramolecular nucleophilic attack of the iminide nitrogen on the ester carbonyl in intermediate **11** to form furo-annulation product **5** did not occur because of two reasons: (1) the additional activation of the electrophilic keto group by the lactone carbonyl group, and (2) stabilization of alcoholate **13** by the chelation of the copper by the lactone carbonyl group. As a result, the cyclization in the keto group proceeded rapidly and irreversibly. The copper–hydrogen exchange between alcoholate **13** and enol **4** afforded the final cycloadduct **6** and regenerated enolate **4**-Cu(I).

**Scheme 6:**
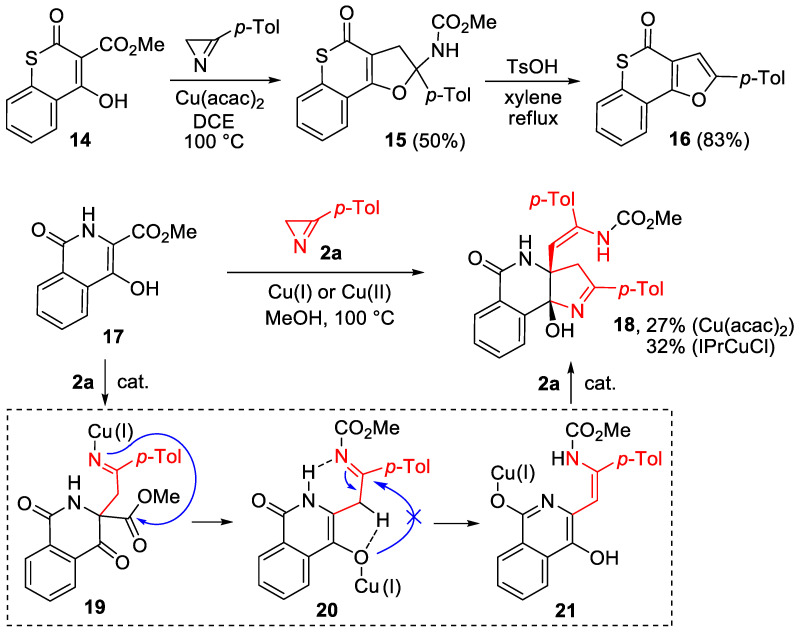
Synthesis of compounds **15**, **16**, and **18**.

Thus, the lactone carbonyl in intermediate **11** acted as a directing group, enabling the annulation of the pyrroline ring even though the enol system bears an ester substituent at the β-C enol atom. The reaction of methoxycarbonyl-substituted 6-membered cyclic enols, having no directing carbonyl group, should proceed through a furo-annulation involving the CO_2_Me group. This important conclusion was supported by the reaction between thiochromene-based enol **14** and azirine **2a** (Scheme 6). This reaction afforded carbamate **15** as a sole product in the presence of copper(II) acetylacetonate under the standard conditions. Compound **15** can be transformed under acidic conditions into thiochromenofuran **16** in good yields.

An unexpected result was obtained in the reaction of 4-hydroxyisoquinolinone **17** with azirine **2a** catalyzed with Cu(acac)_2_ (5 mol%) (Scheme 6). The formation of a furo-fused product (similar to **15**) did not occur from compound **17**, despite the presence of the methoxycarbonyl group at the β-C atom and the absence of the directing carbonyl group at the α-C atom of the enol moiety. Instead, pyrrolino-annulated 1:2 adduct **18** was isolated in 27% yield. Optimization experiments showed that the use of other copper(II) catalysts (Cu(OAc)_2_, Cu(hfacac)_2_) did not enhance the efficiency of the reaction, whereas NHC complex IPrCuCl (5 mol%) allowed a slight increase in the yield of **18** (32%). This product resulted from the addition of two molecules of azirine **2a** to isoquinolone **17**, one of which modified the methoxycarbonyl group of **17** and another one formed the pyrroline ring. The abnormal reaction course can be rationalized in terms of rapid copper–hydrogen exchange in intermediate **20**, which is more rapid than the furan ring closure. The (2+3) cycloaddition of aminovinyl-substituted enol **21** to azirine **2a** afforded compound **18**. The *Z* configuration of the C=C bond in compound **18** was established based on 2D ^1^H-^1^H-NOESY spectrum data (see the Supplementary Materials).

## 3. Materials and Methods

### 3.1. General Instrumentation

Melting points were determined on a melting-point apparatus and were uncorrected. ^1^H (400 MHz) and ^13^C (100 MHz) NMR spectra were recorded on a Bruker Avance 400 spectrometer in solvent indicated below. ^1^H NMR spectra were calibrated according to the residual peaks of CDCl_3_ (δ = 7.26 ppm), DMSO-d_6_ (δ = 2.50 ppm). ^13^C{1H} NMR spectra were calibrated according to the carbon atom peaks of CDCl_3_ (δ = 77.0 ppm), DMSO-d_6_ (δ = 40.0 ppm). High-resolution mass spectra were recorded with a Bruker maXis HRMS-QTOF, electrospray ionization. X-ray diffraction analysis was performed with an Agilent Technologies Xcalibur Eos (for **3e**) and Agilent Technologies Supernova (for **6c**) diffractometers. Crystallographic data for the structures **3e** (CCDC 1535459) and **6c** (CCDC 2182542) were deposited at the Cambridge Crystallographic Data Centre. Thin-layer chromatography (TLC) was conducted on aluminum sheets precoated with SiO_2_ ALUGRAM SIL G/UV_254_. Column chromatography was performed on silica gel 60 M (0.04–0.063 mm). Methanol was refluxed for 2 h with magnesium turnings and then distilled. 1,2-Dichloroethane was washed with concentrated H_2_SO_4_, water, then distilled from P_2_O_5_ and stored over anhydrous K_2_CO_3_.

Quinolones **1a**–**l** were prepared from isatoic anhydrides and corresponding methyl acetates according to the procedure described in the literature [42]. Quinolones **1a**,**l**,**m [43]**, and **1c** [42] are known compounds. Compounds **2a**–**c** [44], **2d** [45], **4 [46]**, **7** [47], **14** [48], and **17** [49] are known compounds, which were prepared by using the reported procedures.

### 3.2. Synthesis and Characterization of Quinolones

*4-Hydroxy-1-methyl-3-(4-methylphenyl)quinolin-2(1H)-one* (**1b**) [42]. Compound **1b** (1.09 g, 82%) was prepared from *N*-methylisatoic anhydride (0.89 g, 5 mmol), methyl 4-methylphenylacetate (0.82 g, 5 mmol), and KHDMS (1.99 g, 10 mmol) as a colorless solid. Mp: 204–205 °C (MeOH). ^1^H NMR (400 MHz, DMSO-d_6_), δ, ppm: 2.35 (s, 3H), 3.60 (s, 3H), 7.20–7.29 (m, 5H), 7.50 (d, *J* = 8.4 Hz, 1H), 7.61–7.65 (m, 1H), 8.02–8.04 (m, 1H), 9.92 (s, 1H). ^13^C NMR (100 MHz, DMSO-d_6_), δ, ppm: 21.4, 29.7, 112.6, 114.8, 116.8, 121.7, 124.0, 128.9, 131.1, 131.3, 131.5, 136.5, 139.3, 156.5, 162.5. HRMS-ESI: [M+H]^+^ calcd for C_17_H_16_NO_2_^+^ 266.1176, found 216.1187.

*3-(4-Fluorophenyl)-4-hydroxy-1-methylquinolin-2(1H)-one* (**1d**) [42]. Compound **1d** (0.89 g, 66%) was prepared from *N*-methylisatoic anhydride (0.89 g, 5 mmol), methyl 4-fluorophenylacetate (0.84 g, 5 mmol), and KHDMS (1.99 g, 10 mmol) as a colorless solid. Mp: 288–290 °C (MeOH). ^1^H NMR (400 MHz, DMSO-d_6_), δ, ppm: 3.61 (s, ^3^H), 7.20–7.24 (m, ^2^H), 7.28 (t, *J* = 7.5 Hz, ^1^H), 7.36–7.39 (m, ^2^H), 7.52 (d, *J* = 8.4 Hz, ^1^H), 7.65 (d, *J* = 7.5 Hz, ^1^H), 8.05 (d, *J* = 7.7 Hz, ^1^H), 10.11 (s, ^1^H). ^13^C NMR (100 MHz, DMSO-d_6_), δ, ppm: 29.7, 111.7, 114.9, 115.1 (d, *J* = 21.2 Hz), 116.7, 121.8, 124.0, 130.4 (d, *J* = 3.2 Hz), 131.5, 133.6 (d, *J* = 8.1 Hz), 139.4, 156.8, 161.8 (d, *J* = 243.0 Hz), 162.4. HRMS-ESI: [M+H]^+^ calcd for C_16_H_13_FNO_2_^+^ 270.0925, found 270.0931.

*3-(4-Chlorophenyl)-4-hydroxy-1-methylquinolin-2(1H)-one* (**1e**) [42]. Compound **1e** (1.24 g, 87%) was prepared from *N*-methylisatoic anhydride (0.89 g, 5 mmol), methyl 4-chlorophenylacetate (0.92 g, 5 mmol), and KHDMS (1.99 g, 10 mmol) as a colorless solid. Mp: 279–281 °C (MeOH). ^1^H NMR (400 MHz, DMSO-d_6_), δ, ppm: 3.60 (s, 3H), 7.28 (t, *J* = 7.5 Hz, 1H), 7.37 (d, *J* = 8.4 Hz, 2H), 7.45 (d, *J* = 8.4 Hz, 2H), 7.51 (d, *J* = 8.4 Hz, ^1^H), 7.65 (t, *J* = 7.3 Hz, 1H), 8.05 (d, *J* = 7.5 Hz, 1H), 10.21 (br. S, 1H). ^13^C NMR (100 MHz, DMSO-d_6_), δ, ppm: 29.7, 111.5, 114.9, 116.7, 121.9, 124.1, 128.2, 131.6, 132.1, 133.1, 133.5, 139.5, 156.9, 162.2. HRMS-ESI: [M+H]^+^ calcd for C_16_H_13_^35^ClNO_2_^+^ 286.0629, found 286.0639.

*4-Hydroxy-1-methyl-3-(4-nitrophenyl)quinolin-2(1H)-one* (**1f**) [42]. Compound **1f** (0.77 g, 52%) was prepared from *N*-methylisatoic anhydride (0.89 g, 5 mmol), methyl 4-nitrophenylacetate (0.98 g, 5 mmol), and KHDMS (1.99 g, 10 mmol) as a colorless solid. Mp: 339–340 °C (MeOH). ^1^H NMR (400 MHz, DMSO-d_6_), δ, ppm: 3.62 (s, 3H), 7.32 (t, *J* = 7.5 Hz, 1H), 7.56 (d, *J* = 8.5 Hz, 1H), 7.67–7.71 (m, 3H), 8.10 (d, *J* = 7.9 Hz, 1H), 8.26 (d, *J* = 8.5 Hz, 2H), 10.61 (br. s, 1H). ^13^C NMR (100 MHz, DMSO-d_6_), δ, ppm: 29.3, 110.9, 114.5, 116.4, 121.5, 122.6, 123.9, 131.6, 132.6, 139.5, 141.8, 146.5, 157.3, 161.5. HRMS-ESI: [M+H]^+^ calcd for C_16_H_13_N_2_O_4_^+^ 297.0870, found 297.0885.

*4-Hydroxy-1-methyl-3-(thiophen-2-yl)quinolin-2(1H)-one* (**1g**) [42]. Compound **1g** (0.71 g, 55%) was prepared from *N*-methylisatoic anhydride (0.89 g, 5 mmol), methyl (thiophen-2-yl)acetate (0.78 g, 5 mmol), and KHDMS (1.99 g, 10 mmol) as a colorless solid. Mp: 175–176 °C (MeOH). ^1^H NMR (400 MHz, DMSO-d_6_), δ, ppm: 3.68 (s, 3H), 7.13 (dd, *J* = 5.1 and 3.8 Hz, 1H), 7.33 (t, *J* = 7.2 Hz, 1H), 7.52 (dd, *J* = 5.2 and 1.0 Hz, 1H), 7.56 (d, *J* = 8.3 Hz, 1H), 7.64–7.68 (m, 1H), 8.01 (dd, *J* = 3.7 and 1.0 Hz, 1H), 8.17 (dd, *J* = 8.1 and 1.0 Hz, 1H), 11.08 (br. s, 1H). ^13^C NMR (100 MHz, DMSO-d_6_), δ, ppm: 30.1, 107.0, 115.1, 116.4, 122.1, 123.9, 126.2, 126.6, 128.7, 131.5, 135.1, 138.4, 156.4, 161.6. HRMS-ESI: [M+H]^+^ calcd for C_14_H_12_NO_2_S^+^ 258.0583, found 258.0595.

*5-Chloro-4-hydroxy-1-methyl-3-phenylquinolin-2(1H)-one* (**1h**) [42]. Compound **1h** (1.05 g, 74%) was prepared from *N*-methyl-5-chloroisatoic anhydride (1.06 g, 5 mmol), methyl phenylacetate (0.75 g, 5 mmol), and KHDMS (1.99 g, 10 mmol) as a colorless solid. Mp: 208–209 °C (MeOH). ^1^H NMR (400 MHz, DMSO-d_6_), δ, ppm: 3.61 (s, 3H), 7.31–7.57 (m, 8H), 9.78 (s, 1H). ^13^C NMR (100 MHz, DMSO-d_6_), δ, ppm: 30.7, 114.3, 114.6, 114.8, 125.7, 127.8, 128.5, 131.1 (2C), 131.7, 133.6, 141.8, 157.0, 161.6. HRMS-ESI: [M+H]^+^ calcd for C_16_H_13_^35^ClNO_2_^+^ 286.0629, found 286.0636.

*6-Chloro-4-hydroxy-1-methyl-3-phenylquinolin-2(1H)-one* (**1i**) [42]. Compound **1i** (0.97 g, 68%) was prepared from *N*-methyl-6-chloroisatoic anhydride (1.06 g, 5 mmol), methyl phenylacetate (0.75 g, 5 mmol), and KHDMS (1.99 g, 10 mmol) as a colorless solid. Mp: 265–267 °C (MeOH). ^1^H NMR (400 MHz, DMSO-d_6_), δ, ppm: 3.59 (s, 3H), 7.31–7.35 (m, 3H), 7.39–7.43 (m, 2H), 7.53 (d, *J* = 9.1 Hz, 1H), 7.65 (dd, *J* = 9.0 and 2.4 Hz, 1H), 8.02 (d, *J* = 2.4 Hz, 1H), 10.28 (br. s, 1H). ^13^C NMR (100 MHz, DMSO-d_6_), δ, ppm: 29.9, 113.8, 117.0, 118.2, 123.1, 126.2, 127.6, 128.2, 131.0, 131.5, 133.8, 138.2, 155.6, 162.2. HRMS-ESI: [M+H]^+^ calcd for C_16_H_13_^35^ClNO_2_^+^ 286.0629, found 286.0640.

*7-Chloro-4-hydroxy-1-methyl-3-phenylquinolin-2(1H)-one* (**1j**) [42]. Compound **1j** (1.0 g, 70%) was prepared from *N*-methyl-7-chloroisatoic anhydride (1.06 g, 5 mmol), methyl phenylacetate (0.75 g, 5 mmol), and KHDMS (1.99 g, 10 mmol) as a colorless solid. Mp: 230–231 °C (MeOH). ^1^H NMR (400 MHz, DMSO-d_6_), δ, ppm: 3.58 (s, 3H), 7.29–7.35 (m, 4H), 7.39–7.43 (m, 2H), 7.57 (d, *J* = 1.7 Hz, 1H), 8.02 (d, *J* = 8.6 Hz, 1H), 10.22 (s, 1H). ^13^C NMR (100 MHz, DMSO-d_6_), δ, ppm: 29.9, 113.0, 114.5, 115.7, 121.8, 125.9, 127.6, 128.3, 131.6, 133.8, 136.1, 140.4, 156.2, 162.4. HRMS-ESI: [M+H]^+^ calcd for C_16_H_13_^35^ClNO_2_^+^ 286.0629, found 286.0638.

*8-Chloro-4-hydroxy-1-methyl-3-phenylquinolin-2(1H)-one* (**1k**) [42]. Compound **1k** (0.87 g, 61%) was prepared from *N*-methyl-8-chloroisatoic anhydride (1.06 g, 5 mmol), methyl phenylacetate (0.75 g, 5 mmol), and KHDMS (1.99 g, 10 mmol) as a colorless solid. Mp: 238–239 °C (MeOH). ^1^H NMR (400 MHz, DMSO-d_6_), δ, ppm: 3.77 (s, 3H), 7.25 (t, *J* = 7.9 Hz, 1H), 7.31–7.43 (m, 5H), 7.69 (d, *J* = 7.7 Hz, 1H), 8.02 (d, *J* = 7.8 Hz, 1H), 10.32 (br. S, 1H). ^13^C NMR (100 MHz, DMSO-d_6_), δ, ppm: 37.3, 113.0, 119.8, 121.0, 123.1, 123.6, 127.6, 128.3, 131.5, 133.7, 134.4, 137.8, 156.5, 164.2. HRMS-ESI: [M+Na]^+^ calcd for C_16_H_12_^35^ClNNaO_2_^+^ 308.0449, found 308.0461.

### 3.3. Synthesis of Pyrrolo[3,2-c]quinolin-4-ones ***3***

General procedure. Quinolone **1a–m** (0.2 mmol), Cu(Oac)_2_·H_2_O (2 mg, 0.01 mmol), azirine **2** (0.32 mmol), and MeOH (3 mL) (or DCE for compound **3p**) were placed consequently into a screw-cap glass tube and heated at 100 °C for 15–20 min under stirring until full consumption of the quinolinone **1** was detected (control by TLC). The solvent was removed in vacuo and the residue was purified by column chromatography on silica gel (eluent hexane/EtOAc from 2:1 to 1:2) followed by recrystallization from hexane/CHCl_3_ to produce adduct **3** as pure crystals (compounds **3c** and **3l**) or as solvates with chloroform (compounds **3a**,**b**,**d**–**k**,**m**–**p**).

*rac-(3aR,9bR)-9b-Hydroxy-3a-(4-methoxyphenyl)-5-methyl-2-(4-methylphenyl)-3,3a,5,9b-tetrahydro-4H-pyrrolo[3,2-c]quinolin-4-one* (**3a**). Colorless solid (**3a** × 0.48CHCl_3_, 91 mg, 97%). Mp: 144–145 °C (CHCl_3_/hexane). ^1^H NMR (400 MHz, DMSO-d_6_), δ, ppm: 2.36 (s, 3H), 3.25 (s, 3H), 3.67 (s, 3H), 3.78 (d, *J* = 16.1 Hz, 1H), 4.27 (d, *J* = Hz, 1H), 6.17 (s, 1H), 6.78 (d, *J* = 8.8 Hz, 2H), 7.12 (d, *J* = 8.8 Hz, 2H), 7.20 (d, *J* = 8.1 Hz, 1H), 7.25–7.30 (m, 3H), 7.43–7.48 (m, 1H), 7.83 (d, *J* = 8.1 Hz, 2H), 7.96 (dd, *J* = 7.6 and 1.3 Hz, 1H). ^13^C NMR (100 MHz, DMSO-d_6_), δ, ppm: 21.5, 30.5, 44.2, 55.4, 60.9, 97.5, 113.8, 115.0, 123.7, 127.2, 128.4 (2C), 129.4, 129.7, 129.9, 130.0, 131.3, 138.3, 142.0, 158.7, 171.5, 175.6. HRMS-ESI: [M+H]^+^ calcd for C_26_H_25_N_2_O_3_^+^ 413.1860, found 413.1858.

*rac-(3aR,9bR)-9b-Hydroxy-5-methyl-2,3a-di(4-methylphenyl)-3,3a,5,9b-tetrahydro-4H-pyrrolo[3,2-c]quinolin-4-one* (**3b**). Colorless solid (**3b** × 1.47CHCl_3,_ 112 mg, 98%). Mp: 178–179 °C (CHCl_3_/hexane). ^1^H NMR (400 MHz, DMSO-d_6_), δ, ppm: 2.21 (s, 3H), 2.36 (s, 3H), 3.25 (s, 3H), 3.82 (d, *J* = 16.1 Hz, 1H), 4.29 (d, *J* = 16.1 Hz, 1H), 6.19 (s, 1H), 7.02 (d, *J* = 7.6 Hz, 2H), 7.10 (d, *J* = 7.6 Hz, 2H), 7.19 (d, *J* = 8.0 Hz, 1H), 7.28–7.30 (m, 3H), 7.45 (t, *J* = 7.2 Hz, 1H), 7.85 (d, *J* = 7.6 Hz, 2H), 7.98 (d, *J* = 7.2 Hz, 1H). ^13^C NMR (100 MHz, DMSO-d_6_), δ, ppm: 21.0, 21.5, 30.5, 44.1, 61.3, 97.5, 115.0, 123.6, 127.2, 128.2, 128.4 (2C), 128.9, 129.7, 129.9, 131.3, 135.0, 136.6, 138.3, 142.0, 171.4, 175.6. HRMS-ESI: [M+H]^+^ calcd for C_26_H_25_N_2_O_2_^+^ 397.1911, found 397.1914.

*rac-(3aR,9bR)-9b-Hydroxy-5-methyl-2-(4-methylphenyl)-3a-phenyl-3,3a,5,9b-tetrahydro-4H-pyrrolo[3,2-c]quinolin-4-one* (**3c**). Pale-yellow solid (76 mg, 99%). Mp: 165–167 °C (CHCl_3_/Et_2_O). ^1^H NMR (400 MHz, CDCl_3_), δ, ppm: 2.44 (s, 3H), 3.33 (br. S, 3H), 3.53–3.57 (m, 1H), 4.11 (br. S, 1H), 4.27–4.29 (m, 1H), 7.10–7.49 (m, 10H), 7.44–7.53 (m, 1H), 7.67–7.80 (m, 1H), 8.06–8.09 (m, 1H). ^13^C NMR (100 MHz, CDCl_3_), δ, ppm: 21.5, 30.4, 44.1, 61.4, 97.7, 114.4, 123.8, 127.0, 127.6 (2C), 128.4 (3C), 129.1, 129.9, 130.1, 136.6, 138.0, 142.5, 170.9, 177.6. HRMS-ESI: [M+H]^+^ calcd for C_25_H_23_N_2_O_2_^+^ 383.1754, found 383.1748.

*rac-(3aR,9bR)-3a-(4-Fluorophenyl)-9b-hydroxy-5-methyl-2-(4-methylphenyl)-3,3a,5,9b-tetrahydro-4H-pyrrolo[3,2-c]quinolin-4-one* (**3d**). Colorless solid (**3d** × 0.56CHCl_3_, 91 mg, 98%). Mp: 148–149 °C (CHCl_3_/hexane). ^1^H NMR (400 MHz, DMSO-d_6_), δ, ppm: 2.36 (s, 3H), 3.26 (s, 3H), 3.82 (d, *J* = 16.2 Hz, 1H), 4.30 (d, *J* = 16.2 Hz, 1H), 6.29 (s, 1H), 7.06 (t, *J* = 8.9 Hz, 2H), 7.21–7.30 (m, 6H), 7.44–7.49 (m, 1H), 7.84 (d, *J* = 8.1 Hz, 2H), 7.97 (dd, *J* = 7.6 and 1.4 Hz, 1H). ^13^C NMR (100 MHz, DMSO-d_6_), δ, ppm: 21.5, 30.6, 44.5, 61.0, 97.5, 115.1 (d, *J* = 21.1 Hz), 115.2, 123.8, 127.2, 128.2, 128.4, 129.7, 130.0, 130.3 (d, *J* = 8.0 Hz), 131.1, 134.3 (d, *J* = 3.1 Hz), 138.1, 142.1, 161.7 (d, *J* = 243.7 Hz), 171.1, 175.5. HRMS-ESI: [M+H]^+^ calcd for C_25_H_22_FN_2_O_2_^+^ 401.1660, found 401.1655.

*rac-(3aR,9bR)-3a-(4-Chlorophenyl)-9b-hydroxy-5-methyl-2-(4-methylphenyl)-3,3a,5,9b-tetrahydro-4H-pyrrolo[3,2-c]quinolin-4-one* (**3e**). Colorless solid (**3e** × 0.45CHCl_3_, 93 mg, 99%). Mp: 202–203 °C (CHCl_3_/hexane). ^1^H NMR (400 MHz, DMSO-d_6_), δ, ppm: 2.35 (s, 3H), 3.27 (s, 3H), 3.84 (d, *J* = 16.2 Hz, 1H), 4.29 (d, *J* = 16.2 Hz, 1H), 6.33 (s, 1H), 7.21–7.31 (m, 8H), 7.44–7.48 (m, 1H), 7.84 (d, *J* = 8.1 Hz, 2H), 7.97 (dd, *J* = 7.6 and 1.4 Hz, 1H). ^13^C NMR (100 MHz, DMSO-d_6_), δ, ppm: 21.5, 30.7, 44.4, 61.2, 97.5, 115.2, 123.9, 127.2, 128.1, 128.4 (2C), 129.7, 130.0, 130.2, 131.1, 132.3, 137.0, 138.1, 142.2, 170.9, 175.4. HRMS-ESI: [M+H]^+^ calcd for C_25_H_22_^35^ClN_2_O_2_^+^ 417.1364, found 417.1369.

*rac-(3aR,9bR)-9b-Hydroxy-5-methyl-2-(4-methylphenyl)-3a-(4-nitrophenyl)-3,3a,5,9b-tetrahydro-4H-pyrrolo[3,2-c]quinolin-4-one* (**3f**). Colorless solid (**3f** × 0.39CHCl_3_, 80 mg, 85%). Mp: 186–187 °C (CHCl_3_/hexane). ^1^H NMR (400 MHz, DMSO-d_6_), δ, ppm: 2.36 (s, 3H), 3.29 (s, 3H), 3.93 (d, *J* = 16.3 Hz, 1H), 4.33 (d, *J* = 16.3 Hz, 1H), 6.48 (s, 1H), 7.25–7.32 (m, 4H), 7.46–7.51 (m, 3H), 7.84 (d, *J* = 8.1 Hz, 2H), 7.96 (dd, *J* = 7.7 and 1.4 Hz, 1H), 8.11 (d, *J* = 8.9 Hz, 2H). ^13^C NMR (100 MHz, DMSO-d_6_), δ, ppm: 21.5, 30.8, 44.6, 61.9, 97.7, 115.4, 123.5, 124.1, 127.3, 127.8, 128.5, 129.7, 129.8, 130.2, 130.9, 137.9, 142.3, 145.5, 147.0, 170.2, 175.2. HRMS-ESI: [M+H]^+^ calcd for C_25_H_22_N_3_O_4_^+^ 428.1605, found 428.1608.

*rac-(3aR,9bR)-9b-Hydroxy-5-methyl-2-(4-methylphenyl)-3a-(thiophen-2-yl)-3,3a,5,9b-tetrahydro-4H-pyrrolo[3,2-c]quinolin-4-one* (**3g**). Colorless solid (**3g** × 0.49CHCl_3_, 82 mg, 92%). Mp: 112–114 °C (CHCl_3_/hexane). ^1^H NMR (400 MHz, DMSO-d_6_), δ, ppm: 2.36 (s, 3H), 3.28 (s, 3H), 3.77 (d, *J* = 15.9 Hz, 1H), 4.28 (d, *J* = 15.9 Hz, 1H), 6.35 (s, 1H), 6.82 (d, *J* = 3.1 Hz, 1H), 6.87–6.89 (m, 1H), 7.21–7.32 (m, 5H), 7.46 (t, *J* = 7.2 Hz, 1H), 7.81 (d, *J* = 8.0 Hz, 2H), 7.93 (d, *J* = 7.5 Hz, 1H). ^13^C NMR (100 MHz, DMSO-d_6_), δ, ppm: 21.5, 30.7, 45.0, 59.2, 97.6, 115.1, 123.8, 125.7, 126.3, 126.6, 127.3, 127.8, 128.4, 129.7, 130.1, 131.0, 138.2, 139.7, 142.2, 170.0, 176.1. HRMS-ESI: [M+H]^+^ calcd for C_23_H_21_N_2_O_2_S^+^ 389.1318, found 389.1333.

*rac-(3aR,9bS)-9-Chloro-9b-hydroxy-5-methyl-2-(4-methylphenyl)-3a-phenyl-3,3a,5,9b-tetrahydro-4H-pyrrolo[3,2-c]quinolin-4-one* (**3h**). Colorless solid (**3h** × 0.60CHCl_3_, 78 mg, 80%). Mp: 146–148 °C (CHCl_3_/hexane). ^1^H NMR (400 MHz, DMSO-d_6_), δ, ppm: 2.37 (s, 3H), 3.25 (s, 3H), 3.73 (d, *J* = 16.4 Hz, 1H), 4.29 (d, *J* = 16.4 Hz, 1H), 5.86 (s, 1H), 7.21–7.33 (m, 9H), 7.44 (t, *J* = 8.2 Hz, 1H), 7.87 (d, *J* = 8.1 Hz, 2H). ^13^C NMR (100 MHz, DMSO-d_6_), δ, ppm: 21.6, 31.6, 43.2, 62.5, 98.4, 114.8, 125.5, 126.9, 127.4, 128.4, 128.5 (2C), 129.7, 130.5, 131.0, 134.3, 138.7, 141.0, 142.3, 170.8, 176.2. HRMS-ESI: [M+H]^+^ calcd for C_25_H_22_^35^ClN_2_O_2_^+^ 417.1364, found 417.1372.

*rac-(3aR,9bR)-8-Chloro-9b-hydroxy-5-methyl-2-(4-methylphenyl)-3a-phenyl-3,3a,5,9b-tetrahydro-4H-pyrrolo[3,2-c]quinolin-4-one* (**3i**). Colorless solid (**3i** × 0.67CHCl_3_, 98 mg, 99%). Mp: 124–126 °C (CHCl_3_/hexane). Compound **3i** can be obtained as a solvate with DMSO-d_6_ by evaporation solution in DMSO-d_6_ with identical NMR spectra. ^1^H NMR (400 MHz, DMSO-d_6_), δ, ppm: 2.36 (s, 3H), 3.25 (s, 3H), 3.86 (d, *J* = 16.3 Hz, 1H), 4.27 (d, *J* = 16.3 Hz, 1H), 6.41 (s, 1H), 7.17–7.31 (m, 8H), 7.52 (dd, *J* = 8.7 and 2.6 Hz, 1H), 7.84 (d, *J* = 8.1 Hz, 2H), 7.91 (d, *J* = 2.6 Hz, 1H). ^13^C NMR (100 MHz, DMSO-d_6_), δ, ppm: 21.5, 30.7, 44.2, 61.5, 97.1, 117.2, 126.8, 127.6, 128.0, 128.3, 128.5 (2C), 129.6, 129.7, 130.5, 131.0, 137.2, 137.6, 142.3, 171.1, 176.1. HRMS-ESI: [M+H]^+^ calcd for C_25_H_22_^35^ClN_2_O_2_^+^ 417.1364, found 417.1376.

*rac-(3aR,9bR)-7-Chloro-9b-hydroxy-5-methyl-2-(4-methylphenyl)-3a-phenyl-3,3a,5,9b-tetrahydro-4H-pyrrolo[3,2-c]quinolin-4-one* (**3j**). Colorless solid (**3j** × 0.47CHCl_3_, 90 mg, 95%). Mp: 120–121 °C (CHCl_3_/hexane). ^1^H NMR (400 MHz, DMSO-d_6_), δ, ppm: 2.36 (s, 3H), 3.27 (s, 3H), 3.88 (d, *J* = 16.2 Hz, 1H), 4.29 (d, *J* = 16.2 Hz, 1H), 6.36 (s, 1H), 7.22–7.36 (m, 9H), 7.85 (d, *J* = 7.7 Hz, 2H), 7.96 (d, *J* = 8.2 Hz, 1H). ^13^C NMR (100 MHz, DMSO-d_6_), δ, ppm: 21.5, 30.7, 44.2, 61.6, 97.2, 115.1, 123.5, 127.3, 127.6, 128.3, 128.4, 128.5, 128.9, 129.7, 131.0, 134.4, 137.7, 139.6, 142.2, 171.5, 175.8. HRMS-ESI: [M+H]^+^ calcd for C_25_H_22_^35^ClN_2_O_2_^+^ 417.1364, found 417.1374.

*rac-(3aR,9bR)-6-Chloro-9b-hydroxy-5-methyl-2-(4-methylphenyl)-3a-phenyl-3,3a,5,9b-tetrahydro-4H-pyrrolo[3,2-c]quinolin-4-one* (**3k**). Colorless solid (**3k** × 0.68CHCl_3_, 98 mg, 99%). Mp: 170–171 °C (CHCl_3_/hexane). ^1^H NMR (400 MHz, DMSO-d_6_), δ, ppm: 2.37 (s, 3H), 3.29 (s, 3H), 3.79 (d, *J* = 15.9 Hz, 1H), 4.31 (d, *J* = 15.9 Hz, 1H), 6.50 (s, 1H), 7.13–7.34 (m, 8H), 7.55 (d, *J* = 7.4 Hz, 1H), 7.85–7.90 (m, 3H). ^13^C NMR (100 MHz, DMSO-d_6_), δ, ppm: 21.6, 38.1, 44.7, 62.3, 97.2, 122.1, 126.1, 126.2, 127.6, 128.2, 128.4, 128.5, 129.7, 131.2, 132.3, 134.1, 136.2, 137.2, 142.3, 173.3, 177.4. HRMS-ESI: [M+H]^+^ calcd for C_25_H_22_^35^ClN_2_O_2_^+^ 417.1364, found 417.1372.

*rac-(3aR,9bR)-5-Benzyl-9b-hydroxy-2-(4-methylphenyl)-3a-phenyl-3,3a,5,9b-tetrahydro-4H-pyrrolo[3,2-c]quinolin-4-one* (**3l**). Colorless solid (87 mg, 95%). Mp: 173–175 °C (CHCl_3_/hexane). ^1^H NMR (400 MHz, DMSO-d_6_), δ, ppm: 2.38 (s, 3H), 3.92 (d, *J* = 16.0 Hz, 1H), 4.34 (d, *J* = 16.0 Hz, 1H), 5.17 (s, 2H), 6.33 (s, 1H), 6.91–6.93 (m, 2H), 7.07–7.12 (m, 4H), 7.21–7.34 (m, 9H), 7.88 (d, *J* = 8.1 Hz, 2H), 7.98 (dd, *J* = 7.6 and 1.5 Hz, 1H). ^13^C NMR (100 MHz, DMSO-d_6_), δ, ppm: 21.6, 44.2, 45.3, 61.9, 97.4, 115.7, 123.9, 126.5, 127.2 (2C), 127.6, 128.4 (2C), 128.5, 128.8, 129.0, 129.7, 129.8, 131.4, 137.1, 137.2, 137.8, 142.1, 171.8, 176.1. HRMS-ESI: [M+Na]^+^ calcd for C_31_H_26_N_2_NaO_2_^+^ 481.1886, found 481.1896.

*rac-(8aR,11aR)-11a-Hydroxy-10-(4-methylphenyl)-8a-phenyl-5,6,9,11a-tetrahydro-4H-pyrido[3,2,1-ij]pyrrolo[3,2-c]quinolin-8(8aH)-one* (**3m**). Colorless solid (**3m** × 1.20CHCl_3_, 106 mg, 96%). Mp: 187–188 °C (CHCl_3_/MeOH). ^1^H NMR (400 MHz, DMSO-d_6_), δ, ppm: 1.79 (s, 2H), 2.36 (s, 3H), 2.81 (br. S, 2H), 3.43–3.45 (m, 1H), 3.85 (d, *J* = 16.1 Hz, 1H), 3.99–4.02 (m, 1H), 4.29 (d, *J* = 16.1 Hz, 1H), 6.15 (s, 1H), 7.15–7.30 (m, 9H), 7.78–7.86 (m, 3H). ^13^C NMR (100 MHz, DMSO-d_6_), δ, ppm: 21.1, 21.6, 27.5, 42.3, 44.0, 61.3, 97.6, 123.2, 125.0, 125.3, 127.4, 128.1, 128.3, 128.4 (2C), 129.7, 130.2, 131.3, 134.0, 138.2, 142.0, 170.6, 175.4. HRMS-ESI: [M+H]^+^ calcd for C_27_H_25_N_2_O_2_^+^ 409.1911, found 409.1916.

*rac-(3aR,9bR)-9b-Hydroxy-5-methyl-2,3a-diphenyl-3,3a,5,9b-tetrahydro-4H-pyrrolo[3,2-c]quinolin-4-one* (**3n**). Colorless solid (**3n** × 0.26CHCl_3_, 79 mg, 99%). Mp: 174–175 °C (CHCl_3_/hexane). ^1^H NMR (400 MHz, DMSO-d_6_), δ, ppm: 3.27 (s, 3H), 3.89 (d, *J* = 16.2 Hz, 1H), 4.32 (d, *J* = 16.2 Hz, 1H), 6.26 (s, 1H), 7.22–7.31 (m, 7H), 7.45–7.55 (m, 4H), 7.95–7.98 (m, 3H). ^13^C NMR (100 MHz, DMSO-d_6_), δ, ppm: 30.6, 44.2, 61.6, 97.6, 115.1, 123.8, 127.1, 127.5, 128.3, 128.4 (3C), 129.1, 130.0, 132.1, 133.8, 138.0, 138.2, 171.2, 175.7. HRMS-ESI: [M+H]^+^ calcd for C_24_H_21_N_2_O_2_^+^ 369.1598, found 369.1592.

*rac-(3aR,9bR)-9b-Hydroxy-2-(4-methoxyphenyl)-5-methyl-3a-phenyl-3,3a,5,9b-tetrahydro-4H-pyrrolo[3,2-c]quinolin-4-one* (**3o**). Colorless solid (**3o** × 0.51CHCl_3_, 83 mg, 91%). Mp: 99–100 °C (CHCl_3_/hexane). ^1^H NMR (400 MHz, DMSO-d_6_), δ, ppm: 3.26 (s, 3H), 3.78–3.82 (m, 4H), 4.26 (d, *J* = 16.1 Hz, 1H), 6.16 (s, 1H), 7.02 (d, *J* = 8.8 Hz, 2H), 7.19–7.29 (m, 7H), 7.44–7.48 (m, 1H), 7.88–7.94 (m, 3H). ^13^C NMR (100 MHz, DMSO-d_6_), δ, ppm: 30.6, 44.0, 55.9, 61.7, 97.4, 114.5, 115.1, 123.7, 126.5, 127.1, 127.4, 128.4 (2C), 128.5, 129.9, 130.2, 138.1, 138.2, 162.4, 171.3, 175.0. HRMS-ESI: [M+H]^+^ calcd for C_25_H_23_N_2_O_3_^+^ 399.1703, found 399.1693.

*rac-(3aR,9bR)-9b-Hydroxy-5-methyl-2-(4-nitrophenyl)-3a-phenyl-3,3a,5,9b-tetrahydro-4H-pyrrolo[3,2-c]quinolin-4-one* (**3p**). Compound **3p** was prepared according to the general procedure using DCE as a solvent. Colorless solid (**3p** × 0.45CHCl_3_, 81 mg, 87%). Mp: 101–102 °C (CHCl_3_/hexane). ^1^H NMR (400 MHz, DMSO-d_6_), δ, ppm: 3.27 (s, 3H), 3.96 (d, *J* = 16.5 Hz, 1H), 4.34 (d, *J* = 16.5 Hz, 1H), 6.40 (s, 1H), 7.21–7.31 (m, 7H), 7.47–7.51 (m, 1H), 7.95–7.98 (m, 1H), 8.20 (d, *J* = 8.8 Hz, 2H), 8.31–8.33 (m, 2H). ^13^C NMR (100 MHz, DMSO-d_6_), δ, ppm: 30.7, 44.5, 61.7, 97.9, 115.2, 123.9, 124.3, 127.2, 127.6, 127.7, 128.3, 128.5, 129.7, 130.2, 137.6, 138.3, 139.3, 149.6, 171.0, 174.6. HRMS-ESI: [M+H]^+^ calcd for C_24_H_20_N_3_O_4_^+^ 414.1448, found 414.1440.

### 3.4. Synthesis of Chromenopyrroles ***6***

General procedure. Hydroxycoumarin **4** (0.2 mmol), Cu(Oac)_2_ × H_2_O (2 mg, 0.01 mmol), azirine **2** (0.32 mmol), and DCE (3.0 mL) were consequently placed into a screw-cap glass tube and heated at 100 °C for 20–30 min until the full consumption of 3-hydroxycoumarin **4** was detected (control by TLC). The solvent was removed in vacuo and the residue was purified by column chromatography on silica gel (eluent hexane/EtOAc from 2:1 to 1:2), followed by recrystallization from a hexane–Et_2_O mixture to produce compound **6**.

*Methyl rac-(3aR,9bR)-3a-hydroxy-2-(4-methylphenyl)-4-oxo-3a,4-dihydrochromeno[3,4-b]pyrrole-9b(1H)-carboxylate* (**6a**). Colorless solid (47 mg, 67%). Mp: 203–204 °C (Et_2_O/hexane). ^1^H NMR (400 MHz, DMSO-d_6_), δ, ppm: 2.32 (s, 3H), 3.59 (s, 3H), 4.02 and 3.88 (AB-q, *J* = 17.2 Hz, 2H), 7.11 (d, *J* = 8.1 Hz, 1H), 7.18 (t, *J* = 7.4 Hz, 1H), 7.26 (d, *J* = 7.9 Hz, 2H), 7.34 (t, *J* = 7.4 Hz, 1H), 7.46 (d, *J* = 7.5 Hz, 1H), 7.59 (s, 1H), 7.78 (d, *J* = 8.0 Hz, 2H). ^13^C NMR (100 MHz, DMSO-d_6_), δ, ppm: 21.6, 44.6, 53.2, 60.8, 95.5, 117.4, 122.7, 125.8, 128.3, 128.7, 129.8, 130.0, 130.7, 143.3, 150.3, 166.2, 169.7, 179.8. HRMS-ESI: [M-H]^−^ calcd for C_20_H_16_NO_5_^−^ 350.1034, found 350.1039.

*Methyl rac-(3aR,9bR)-3a-hydroxy-4-oxo-2-phenyl-3a,4-dihydrochromeno[3,4-b]pyrrole-9b(1H)-carboxylate* (**6b**). Colorless solid (47 mg, 70%). Mp: 171–172 °C (Et_2_O/hexane). ^1^H NMR (400 MHz, DMSO-d_6_), δ, ppm: 3.59 (s, 3H), 3.92 and 4.07 (AB-q, *J* = 17.3 Hz, 2H), 7.12 (dd, *J* = 8.2 and 1.0 Hz, 1H), 7.19 (td, *J* = 7.6 and 1.1 Hz, 1H), 7.32–7.36 (m, 1H), 7.43–7.48 (m, 3H), 7.54 (t, *J* = 7.4 Hz, 1H), 7.66 (s, 1H), 7.88–7.90 (m, 2H). ^13^C NMR (100 MHz, DMSO-d_6_), δ, ppm: 44.7, 53.3, 60.9, 95.5, 117.4, 122.6, 125.8, 128.3, 128.7, 129.3, 130.7, 132.6, 133.0, 150.3, 166.1, 169.7, 180.1. HRMS-ESI: [M+H]^+^ calcd for C_19_H_16_NO_5_^+^ 338.1023, found 338.1039.

*Methyl rac-(3aR,9bR)-3a-hydroxy-2-(4-methoxyphenyl)-4-oxo-3a,4-dihydrochromeno[3,4-b]pyrrole-9b(1H)-carboxylate* (**6c**). Colorless solid (44 mg, 60%). Mp: 199–200 °C (Et_2_O/hexane). ^1^H NMR (400 MHz, DMSO-d_6_), δ, ppm: 3.58 (s, 3H), 3.79 (s, 3H), 3.87 and 4.00 (AB-q, *J* = 17.1 Hz, 2H), 6.98 (d, *J* = 8.6 Hz, 2H), 7.11 (d, *J* = 8.1 Hz, 1H), 7.18 (t, *J* = 7.4 Hz, 1H), 7.34 (t, *J* = 7.5 Hz, 1H), 7.45 (d, *J* = 7.6 Hz, 1H), 7.52 (s, 1H), 7.84 (d, *J* = 8.6 Hz, 2H). ^13^C NMR (100 MHz, DMSO-d_6_), δ, ppm: 44.5, 53.2, 55.9, 60.9, 95.4, 114.6, 117.4, 122.8, 125.3, 125.7, 128.3, 130.6, 130.7, 150.3, 163.1, 166.3, 169.8, 179.2. HRMS-ESI: [M+H]^+^ calcd for C_20_H_18_NO_6_^+^ 368.1129, found 368.1138.

*Methyl rac-(3aR,9bR)-3a-hydroxy-2-(4-nitrophenyl)-4-oxo-3a,4-dihydrochromeno[3,4-b]pyrrole-9b(1H)-carboxylate* (**6d**). Colorless solid (47 mg, 62%). Mp: 190–191 °C (Et_2_O/hexane). ^1^H NMR (400 MHz, DMSO-d_6_), δ, ppm: 3.61 (s, 3H), 3.99 and 4.16 (AB-q, *J* = 17.5 Hz, 2H), 7.13 (dd, *J* = 8.2 and 1.0 Hz, 1H), 7.20 (td, *J* = 7.7 and 1.1 Hz, 1H), 7.34–7.38 (m, 1H), 7.47 (dd, *J* = 7.8 and 1.3 Hz, 1H), 7.86 (s, 1H), 8.13 (d, *J* = 8.9 Hz, 2H), 8.27 (d, *J* = 8.9 Hz, 2H). ^13^C NMR (100 MHz, DMSO-d_6_), δ, ppm: 45.1, 53.4, 61.0, 95.6, 117.5, 122.2, 124.3, 125.9, 128.4, 130.1, 130.9, 138.0, 150.1, 150.2, 165.8, 169.5, 178.9. HRMS-ESI: [M-H]^−^ calcd for C_19_H_13_N_2_O_7_^−^ 381.0728, found 381.0720.

### 3.5. Synthesis of Methyl Rac-(7aR,10aR)-7a-hydroxy-9-(4-methylphenyl)-7oxo-7a,10-dihydronaphtho[1,8-ef]indole-10a(7H)-carboxylate (***8***)

Enol **7** (51 mg, 0.2 mmol), Cu(hfacac)_2_ (10 mg, 0.02 mmol), azirine **2**a (42 mg, 0.32 mmol), and DCE (3.0 mL) were consequently placed into a screw-cap glass tube and heated at 100 °C for 2.5 h until the full consumption of enol **7** was detected (control by TLC). The solvent was removed in vacuo and the residue was purified by column chromatography on silica gel (eluent hexane/EtOAc from 2:1 to 1:2). Yellow solid (46 mg, 60%). Mp: 208–209 °C (Et_2_O/hexane). ^1^H NMR (400 MHz, CDCl_3_–DMSO-d_6_ mixture), δ, ppm: 2.26 (s, 3H), 3.49 (s, 3H), 3.83 (d, *J* = 16.9 Hz, 1H), 4.32 (d, *J* = 16.9 Hz, 1H), 5.82 (s, 1H), 7.07 (d, *J* = 7.9 Hz, 2H), 7.48–7.52 (m, 1H), 7.60–7.64 (m, 4H), 7.80–7.82 (m, 1H), 8.08–8.10 (m, 1H), 8.32–8.34 (m, 1H). ^13^C NMR (100 MHz, CDCl_3_–DMSO-d_6_ mixture), δ, ppm 20.7, 46.0, 51.9, 63.0, 97.0, 125.4, 125.8, 125.9, 126.3, 127.1, 127.3, 127.6, 128.4, 129.5, 130.1, 132.3, 132.6, 134.4, 141.8, 169.9, 177.8, 193.3. HRMS-ESI: [M+H]^+^ calcd for C_24_H_20_NO_4_^+^ 386.1387, found 386.1393.

### 3.6. Synthesis of Methyl (2-(4-Methylphenyl)-4-oxo-2,3-dihydro-4H-thiochromeno[4,3-b]furan-2-yl)carbamate (***15***)

Enol **14** (60 mg, 0.25 mmol), Cu(acac)_2_ (3 mg, 0.01 mmol), azirine **2a** (54 mg, 0.41 mmol), and DCE (3.0 mL) were consequently placed into a screw-cap glass tube and heated at 100 °C for 20 min until the full consumption of enol **14** was detected (control by TLC). The solvent was removed in vacuo and the residue was purified by column chromatography on silica gel (eluent hexane/EtOAc 4:1). Colorless solid (46 mg, 50%). Mp: 183–184 °C (Et_2_O/hexane). ^1^H NMR (400 MHz, CDCl_3_), δ, ppm: 2.38 (s, 3H), 3.46 (d, *J* = 16.1 Hz, 1H), 3.68 (s, 3H), 4.05 (d, *J* = 16.1 Hz, 1H), 6.04 (s, 1H), 7.23 (d, *J* = 7.8 Hz, 2H), 7.42–7.48 (m, 3H), 7.50–7.56 (m, 2H), 8.13 (d, *J* = 7.8 Hz, 1H). ^13^C NMR (100 MHz, CDCl_3_), δ, 21.0, 41.2, 52.3, 98.9, 111.7, 119.9, 124.3, 125.5, 125.9, 126.1, 129.6, 130.4, 138.9, 139.1, 139.5, 154.5, 164.8, 180.1. HRMS-ESI: [M+Na]^+^ calcd for C_20_H_17_NNaO_4_S^+^ 390.0770, found 390.0770.

### 3.7. Synthesis of 2-(4-Methylphenyl)-4H-thiochromeno[4,3-b]furan-4-one (***16***)

A solution of carbamate **15** (38 mg, 0.1 mmol) and anhydrous *p*-toluenesulfonic acid (2 mg, 0.01 mmol) in anhydrous *o*-xylene (2.0 mL) was refluxed for 15 min. The reaction mixture was diluted with EtOAc (10 mL), washed with 0.1M NaOH, and dried over Na_2_SO_4_. Following filtration and concentration under vacuum, the residue was purified by flash column chromatography on silica gel (eluent hexane/EtOAc 5:1) to produce compound **16**. Colorless solid (25 mg, 83%). Mp: 172–173 °C (Et_2_O/hexane). ^1^H NMR (400 MHz, CDCl_3_), δ, ppm: 2.43 (s, 3H), 7.13 (s, 1H), 7.27–7.29 (m, 2H), 7.45–7.56 (m, 3H), 7.71 (d, *J* = 8.0 Hz, 2H), 8.22–8.25 (m, 1H). ^13^C NMR (100 MHz, CDCl_3_), δ, 21.4, 100.6, 120.6, 121.3, 123.3, 124.5, 125.6, 126.3, 126.6, 128.7, 129.7, 135.2, 139.3, 155.7, 157.5, 180.4. HRMS-ESI: [M+Na]^+^ calcd for C_18_H_13_NaO_2_S^+^ 293.0631, found 293.0633.

### 3.8. Synthesis of Methyl (2-(rac-(3aR,9bR)-9b-Hydroxy-2-(4-methylphenyl)-5-oxo-3,4,5,9b-tetrahydro-3aH-pyrrolo[3,2-c]isoquinolin-3a-yl)-1-(4-methylphenyl)vinyl)carbamate (***18***)

Enol **17** (44 mg, 0.2 mmol), IPrCuCl (5 mg, 0.01 mmol), azirine **2a** (42 mg, 0.32 mmol), and MeOH (3.0 mL) were consequently placed into a screw-cap glass tube and heated at 100 °C for 30 min until the full consumption of enol **17** was detected (control by TLC). The solvent was removed in vacuo and the residue was purified by column chromatography on silica gel (eluent hexane/EtOAc from 2:1 to 1:2). Light-yellow oil (31 mg, 32%). ^1^H NMR (400 MHz, DMSO-d_6_), δ, ppm: 2.27 (s, 3H), 2.37 (s, 3H), 3.35 (d, *J* = 17.0 Hz, 1H), 3.42 (d, *J* = 17.0 Hz, 1H), 3.54 (s, 3H), 5.48 (s, 1H), 6.29 (s, 1H), 7.10 (d, *J* = 8.0 Hz, 2H), 7.27 (d, *J* = 8.1 Hz, 2H), 7.32 (d, *J* = 8.0 Hz, 2H), 7.44–7.48 (m, 1H), 7.64–7.67 (s, 1H), 7.78–7.80 (m, 3H), 7.92–7.95 (m, 1H), 8.74 (s, 1H), 9.16 (br. s, 1H). ^13^C NMR (100 MHz, DMSO-d_6_), δ, ppm: 21.2, 21.6, 51.4, 52.1, 79.0, 90.2, 115.9, 126.0, 126.6, 126.8, 127.5, 128.2, 128.3, 128.9, 129.7, 131.1, 132.8, 136.1, 137.6, 138.5, 141.9, 142.2, 154.8, 161.8, 175.6. HRMS-ESI: [M+Na]^+^ calcd for C_29_H_27_N_3_NaO_4_^+^ 504.1894, found 504.1897.

## 4. Conclusions

In conclusion, we described an effective one-step procedure for the [2+3] pyrroline annulation to six-membered non-aromatic enols with 3-aryl-2*H* azirines as annulation agents. The reaction could be catalyzed by both Cu(II) and Cu(I) compounds. It proceeded as a formal (2+3) cycloaddition via the N1–C2 azirine bond cleavage with high-atom economy and efficiency. The method can be applied to cyclic enols of the quinolin-2-one, 2*H*-chromen-2-one, and 1*H*-phenalen-1-one series. The reaction outcome can be rationalized from the reactivity of the aminide intermediate, which was obtained following the copper(I) enolate-induced azirine-ring opening.

## Figures and Tables

**Table 1:** Optimization of synthesis of 3a.

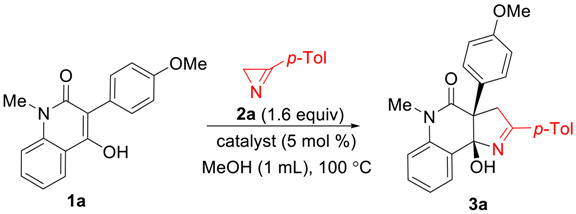			
Entry	Catalyst	Deviation from Standard Conditions	Yield of 3a, % ^a^
1	-		0
2	IPrCuCl ^b^		98
3	CuCl_2_·2H_2_O	**2a** (2 equiv)	95
4	Cu(acac)_2_		98
5	Cu(OAc)_2_·H_2_O		98
6	Cu(OAc)_2_·H_2_O	**2a** (1 equiv)	65
7	Cu(OAc)_2_·H_2_O	solvent EtOH	77
8	Cu(OAc)_2_·H_2_O	temperature 20 °C	53 ^c^
9	Co(OAc)_2_·4H_2_O		17
10	Ni(acac)_2_		64
11	Fe(acac)_3_		70

^a^ Yield is determined by ^1^H NMR spectroscopy using CH_2_Br_2_ as an internal standard. ^b^ IPrCuCl = Chloro[1,3-bis(2,6-diisopropylphenyl)imidazol-2-ylidene]copper(I). ^c^ Reaction time: 48 h.

## Data Availability

Data are contained within the article.

## Supplementary Materials

The following supporting information can be downloaded at: https://www.mdpi.com/article/10.3390/molecules27175681/s1: X-ray diffraction experiments; NMR spectra of compounds **1**, **3**, **6**, **8**, **18** [50,51,52,53].

## Sample Availability

Samples of compounds **3**, **6**, **8**, **15**–**18** are available from the authors.
