# Supplementary material for: Copper(II)-Catalyzed (3+2) Cycloaddition of 2H-Azirines to Six-Membered Cyclic Enols as a Route to Pyrrolo[3,2-c]quinolone, Chromeno[3,4-b]pyrrole, and Naphtho[1,8-ef]indole Scaffolds
Source: Molecules. 2022 Sep 2;27(17):5681. doi: 10.3390/molecules27175681 (PMC9457675; doi:10.3390/molecules27175681)
Supplement: Supplementary file 1 [file molecules-27-05681-s001.zip › molecules-1894159-supplementary.pdf]

*Supporting information for*

# Copper(II)-catalyzed (3+2) cycloaddition of 2*H*-azirines to 6-membered cyclic enols as a route to pyrrolo[3,2-*c*]quinolone, chromeno[3,4-*b*]pyrrole, and naphtho[1,8-*ef*]indole scaffolds

Pavel A. Sakharov, Nikolai V. Rostovskii,* Alexander F. Khlebnikov, and Mikhail S. Novikov*

St. Petersburg State University, Institute of Chemistry, 7/9 Universitetskaya nab.,
St. Petersburg, 199034, Russia.

E-mail: *n.rostovskiy@spbu.ru*, *m.novikov@spbu.ru*

**Contents:**

1. NMR Spectra of New Compounds .......... S-2

2. X-ray Data .......... S-39

3. References .......... S-43

# NMR Spectra of New Compounds

$^{1}H$ NMR (400 MHz, DMSO-$d_6$) spectrum of compound **1b**

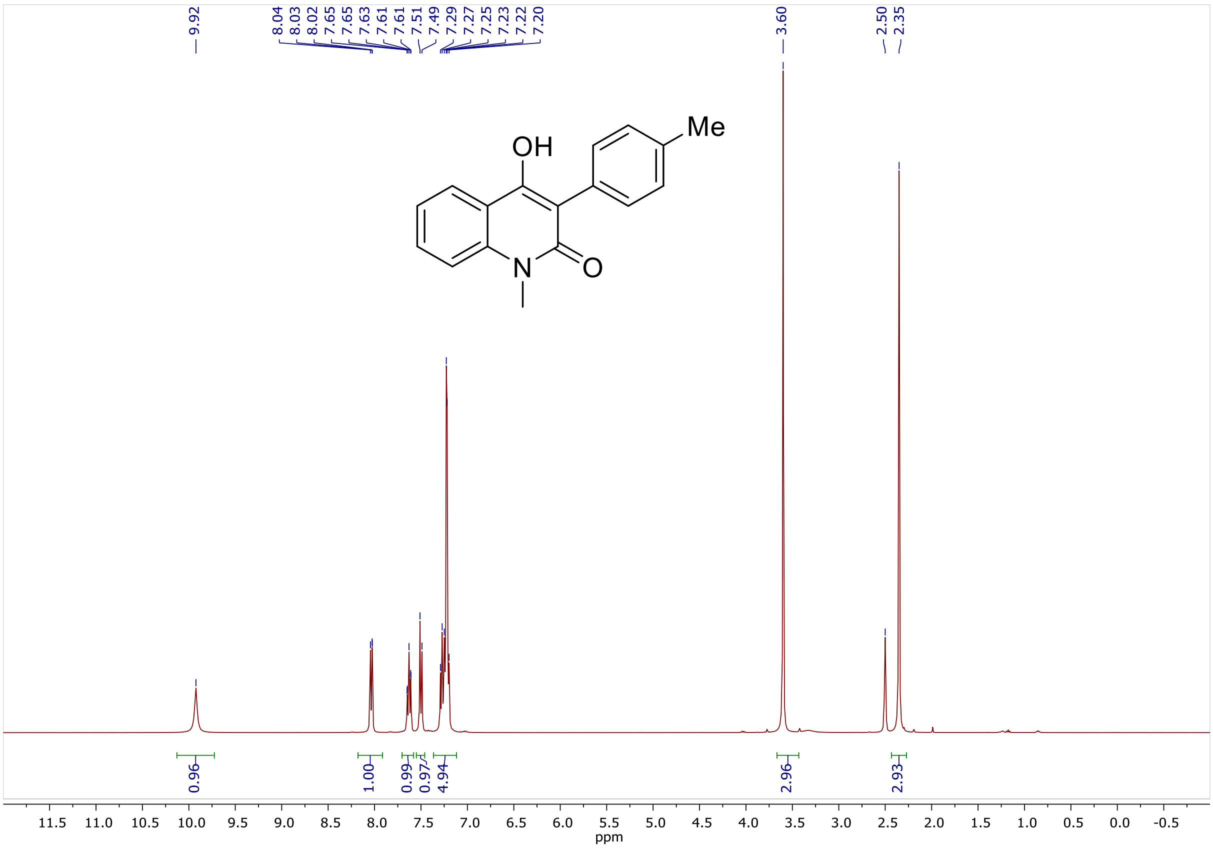


$^{13}C$ NMR (100 MHz, DMSO-$d_6$) spectrum of compound **1b**

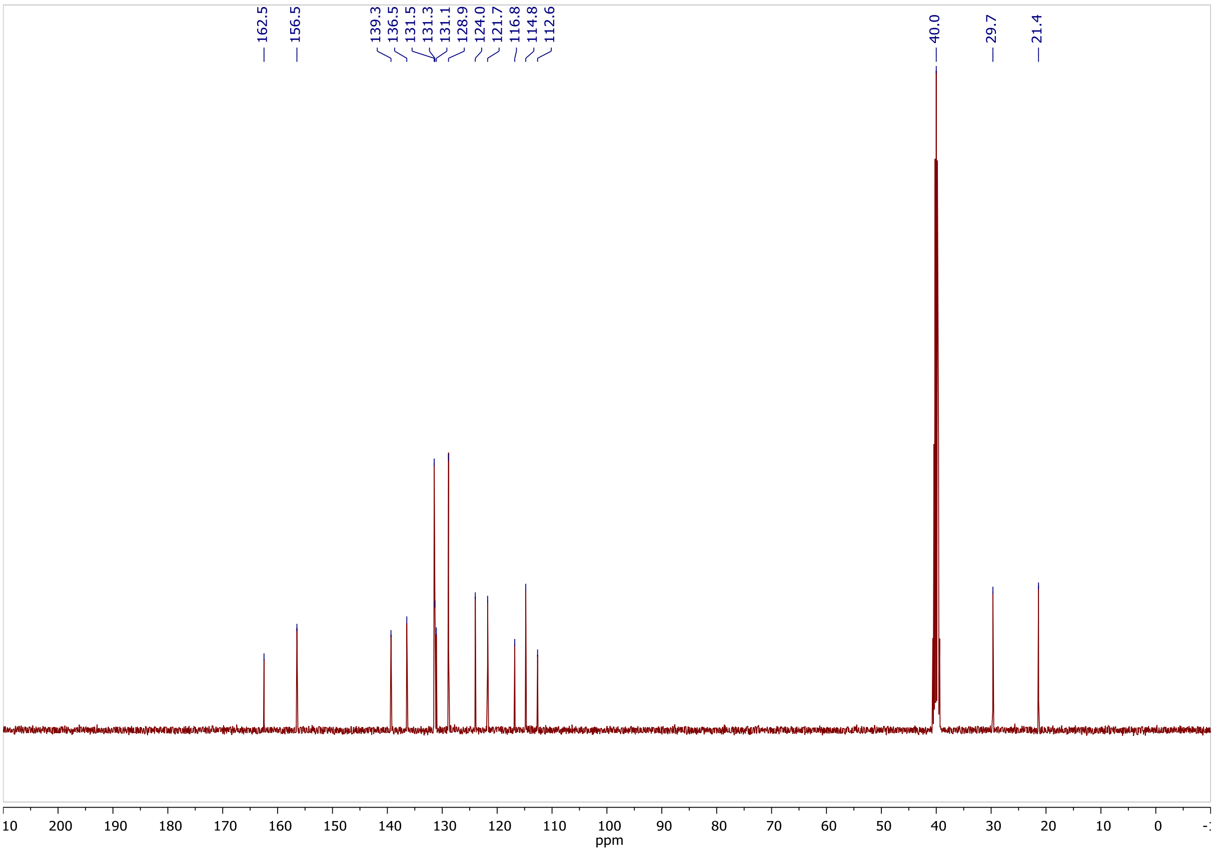

$^{1}H$ NMR (400 MHz, DMSO-$d_6$) spectrum of compound **1d**

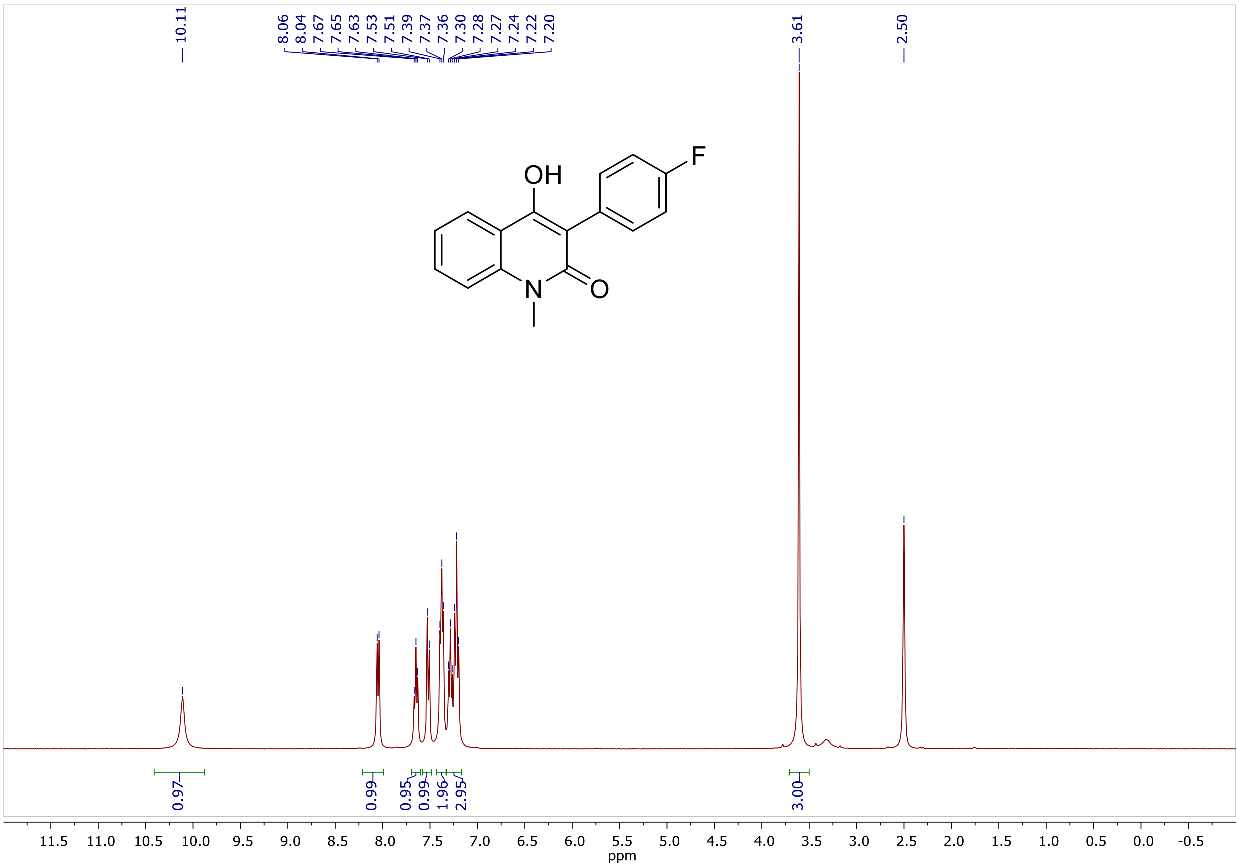


$^{13}C$ NMR (100 MHz, DMSO-$d_6$) spectrum of compound **1d**

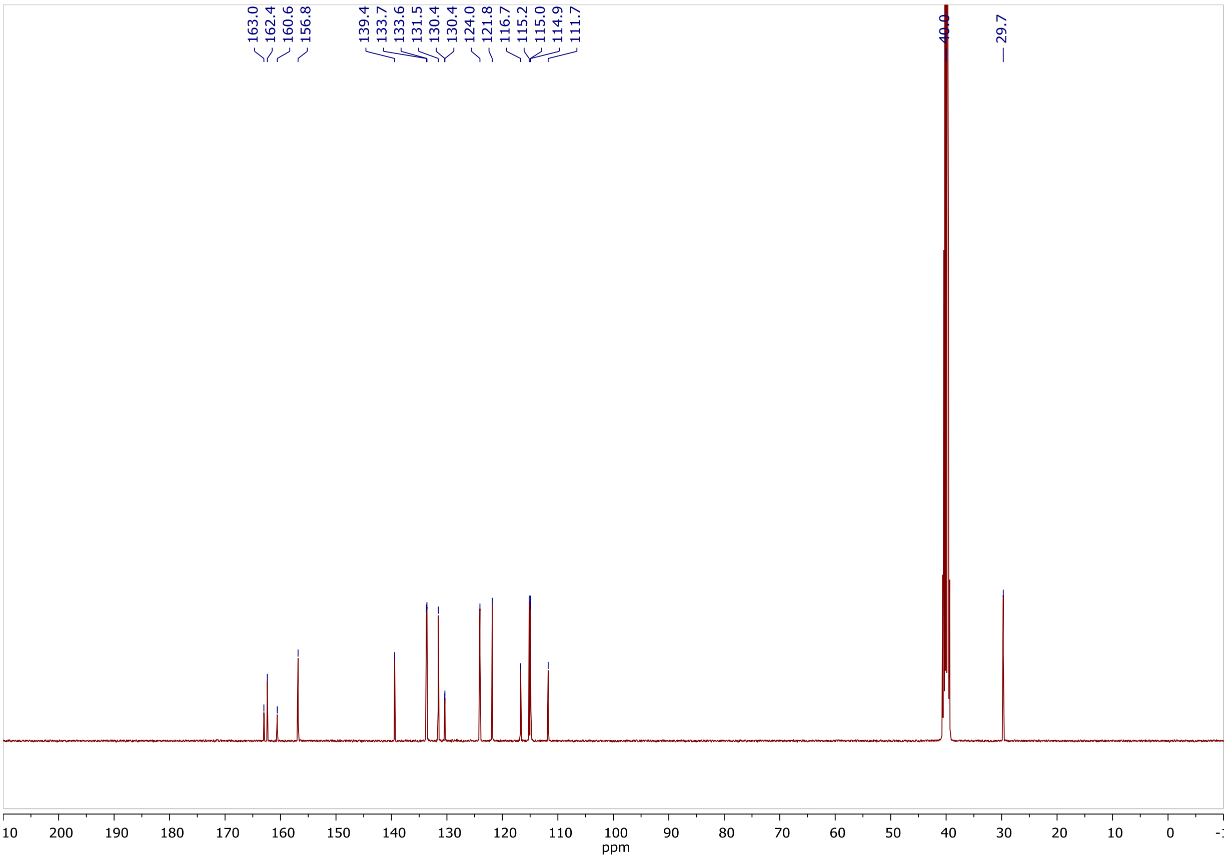

$^{1}H$ NMR (400 MHz, DMSO-d$_6$) spectrum of compound **1e**

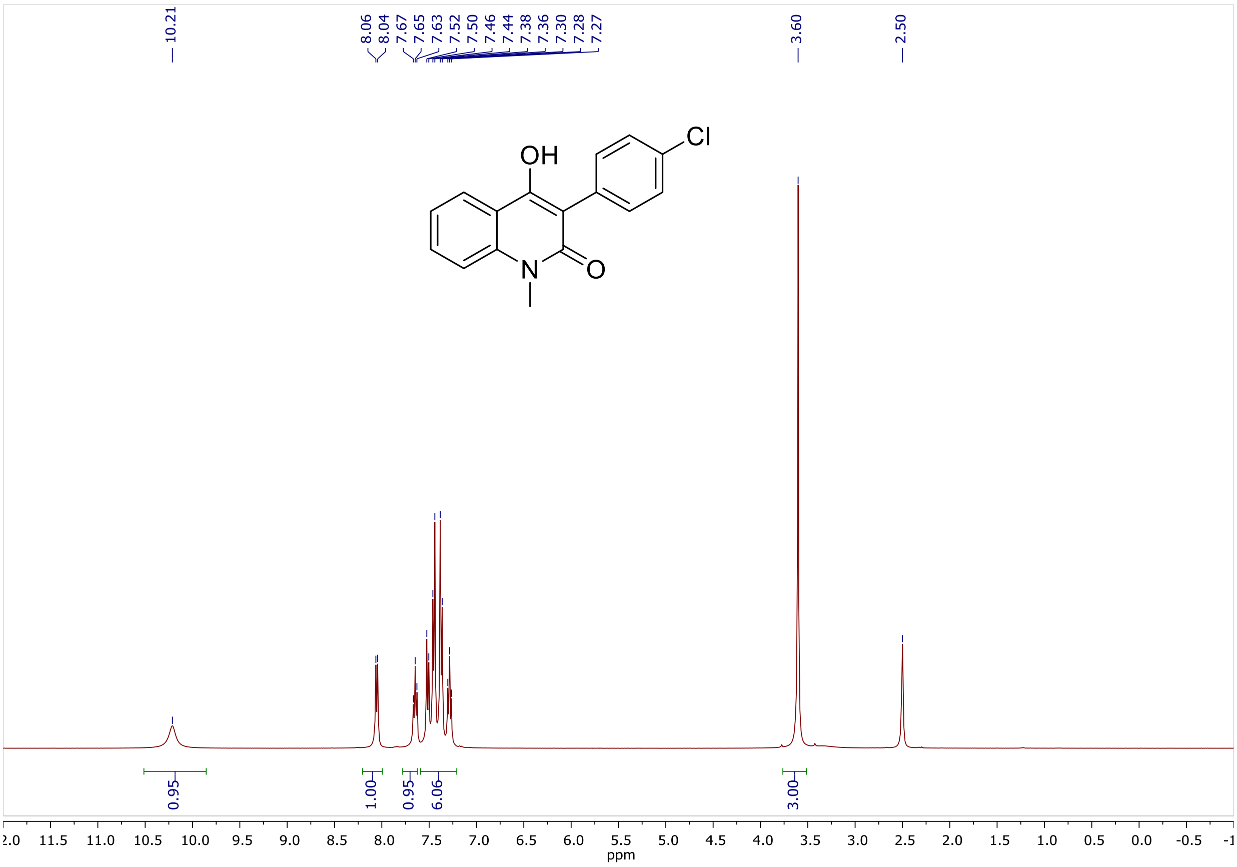


$^{13}C$ NMR (100 MHz, DMSO-d$_6$) spectrum of compound **1e**

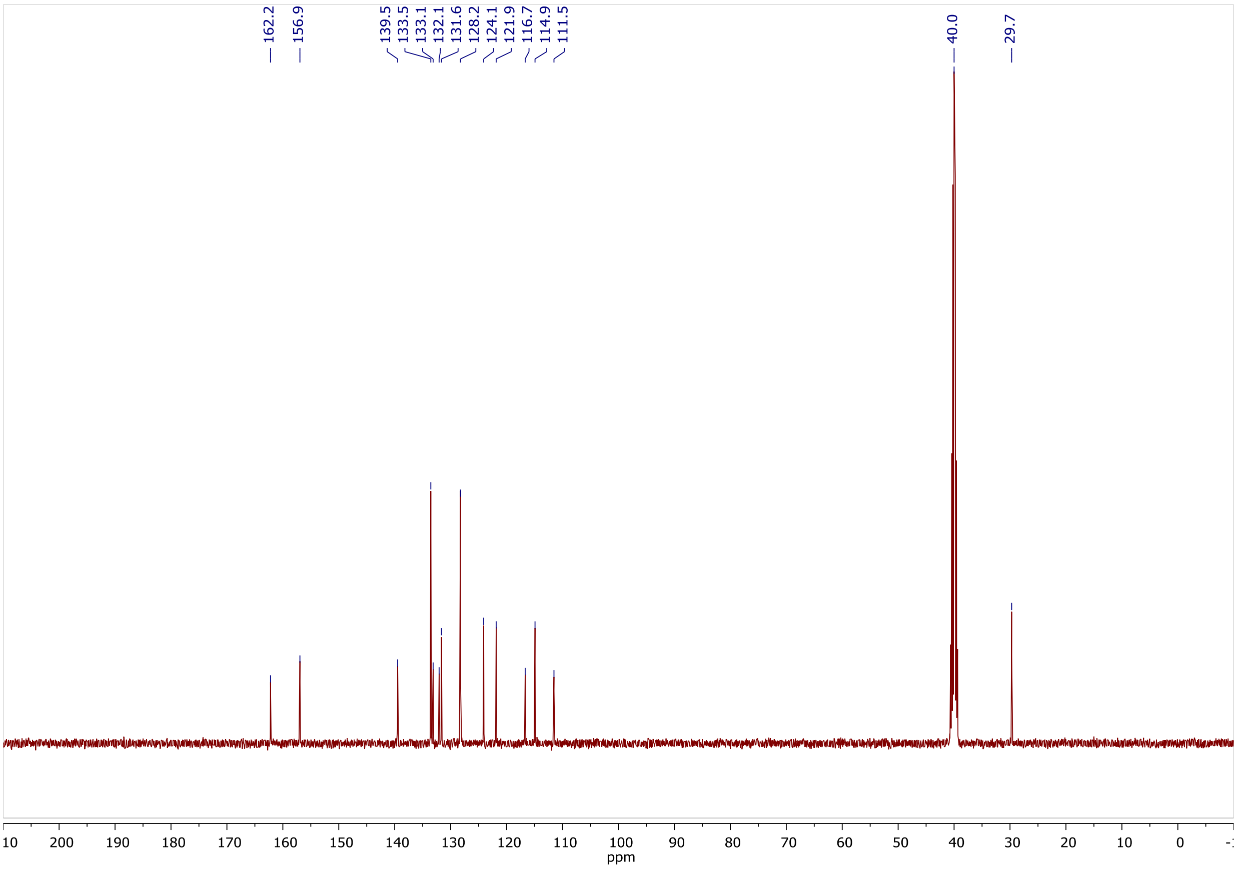

$^{1}H$ NMR (400 MHz, DMSO-$d_6$) spectrum of compound **1f**

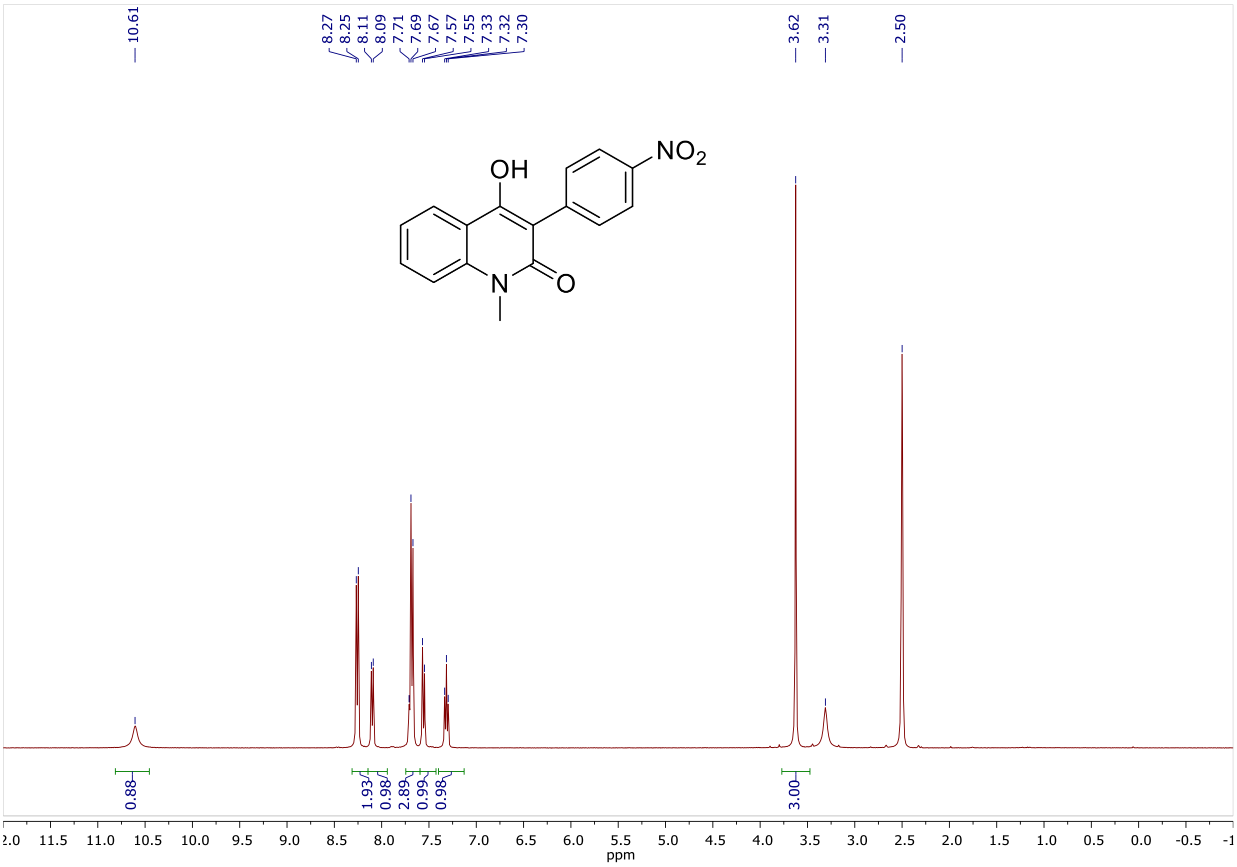


$^{13}C$ NMR (100 MHz, DMSO-$d_6$) spectrum of compound **1f**

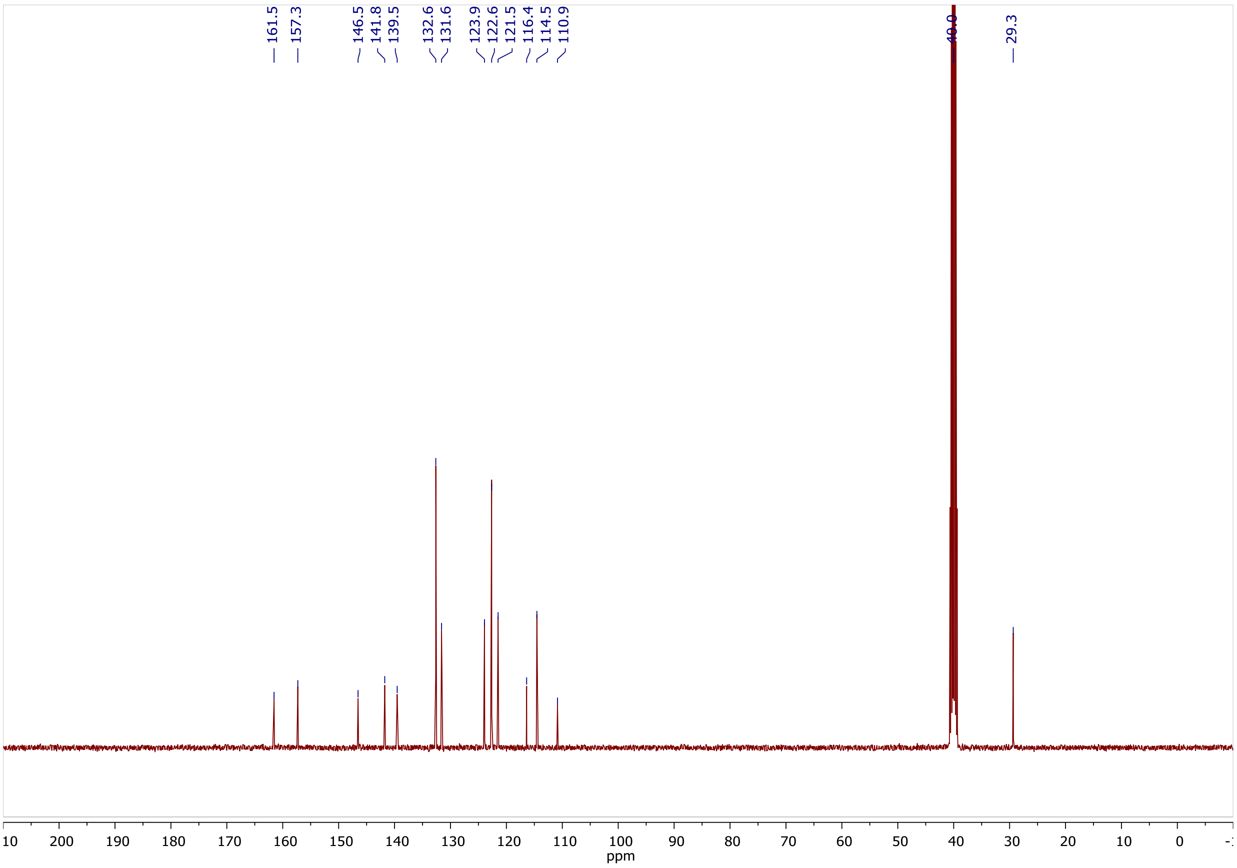

$^{1}H$ NMR (400 MHz, DMSO-$d_6$) spectrum of compound **1g**

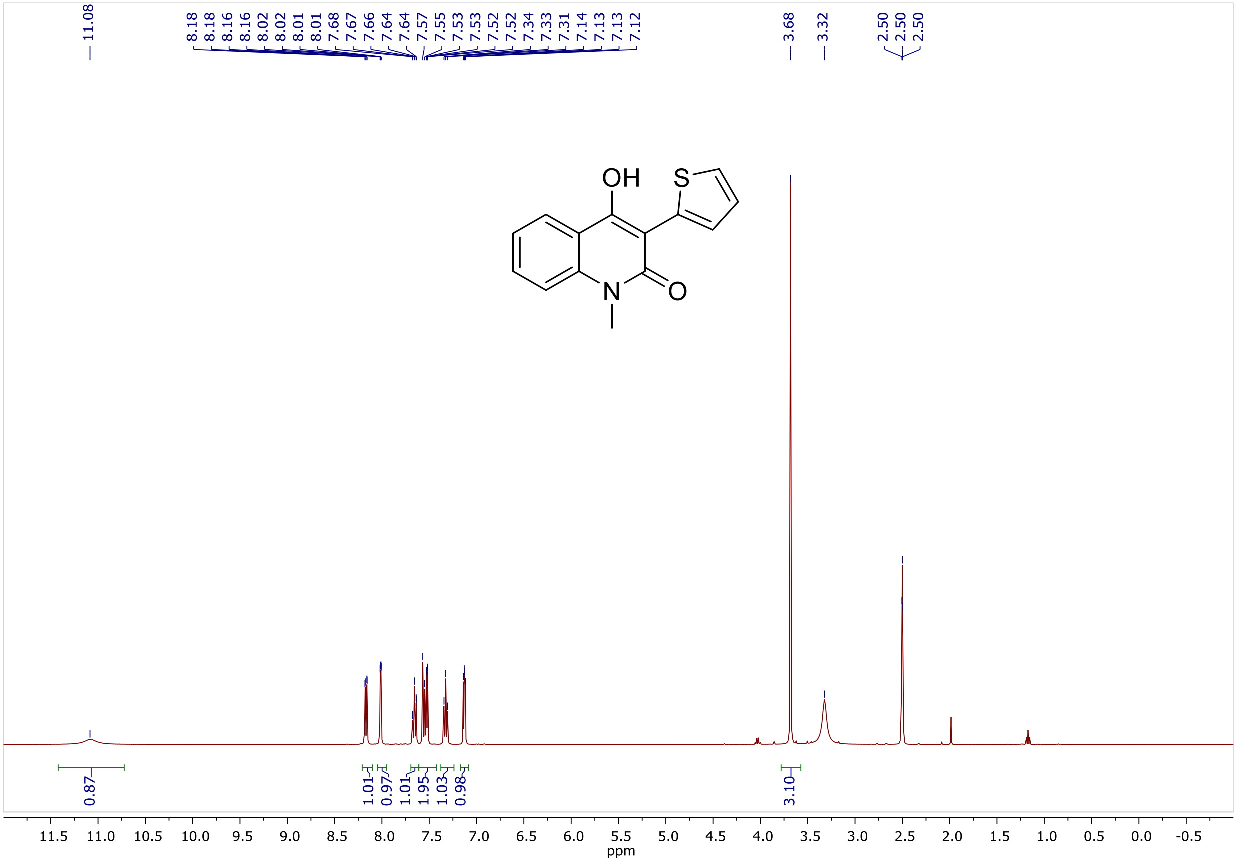


$^{13}C$ NMR (100 MHz, DMSO-$d_6$) spectrum of compound **1g**

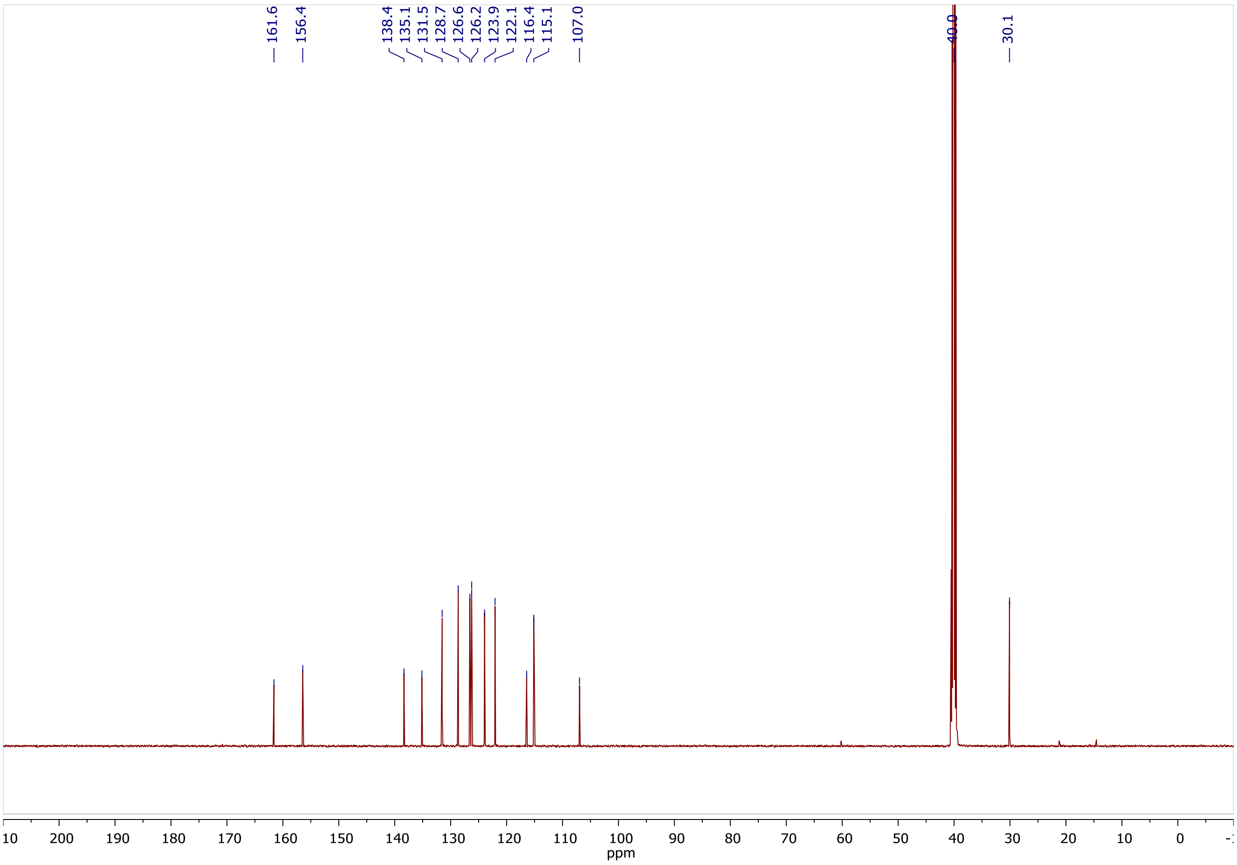

$^{1}$H NMR (400 MHz, DMSO-$d_6$) spectrum of compound **1h**

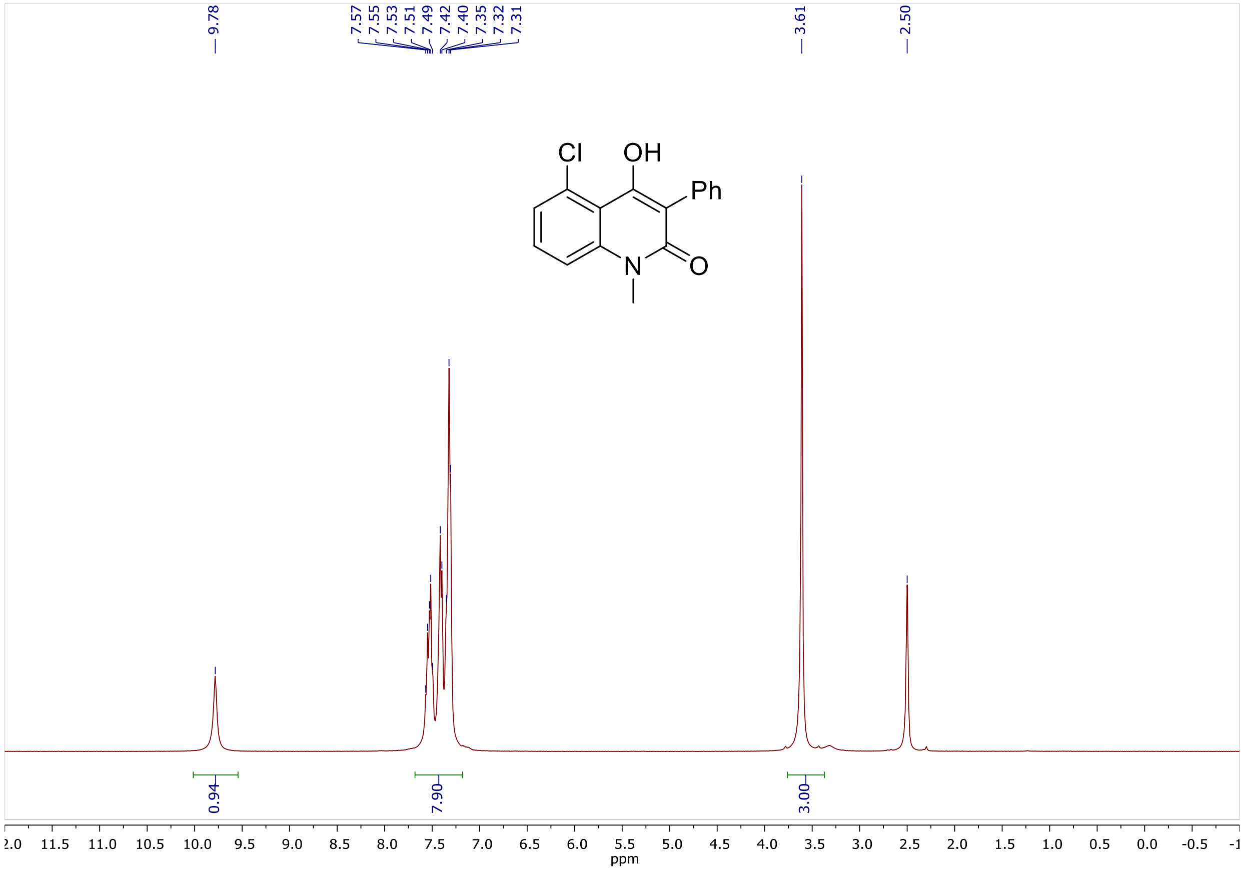

$^{13}$C NMR (100 MHz, DMSO-$d_6$) spectrum of compound **1h**

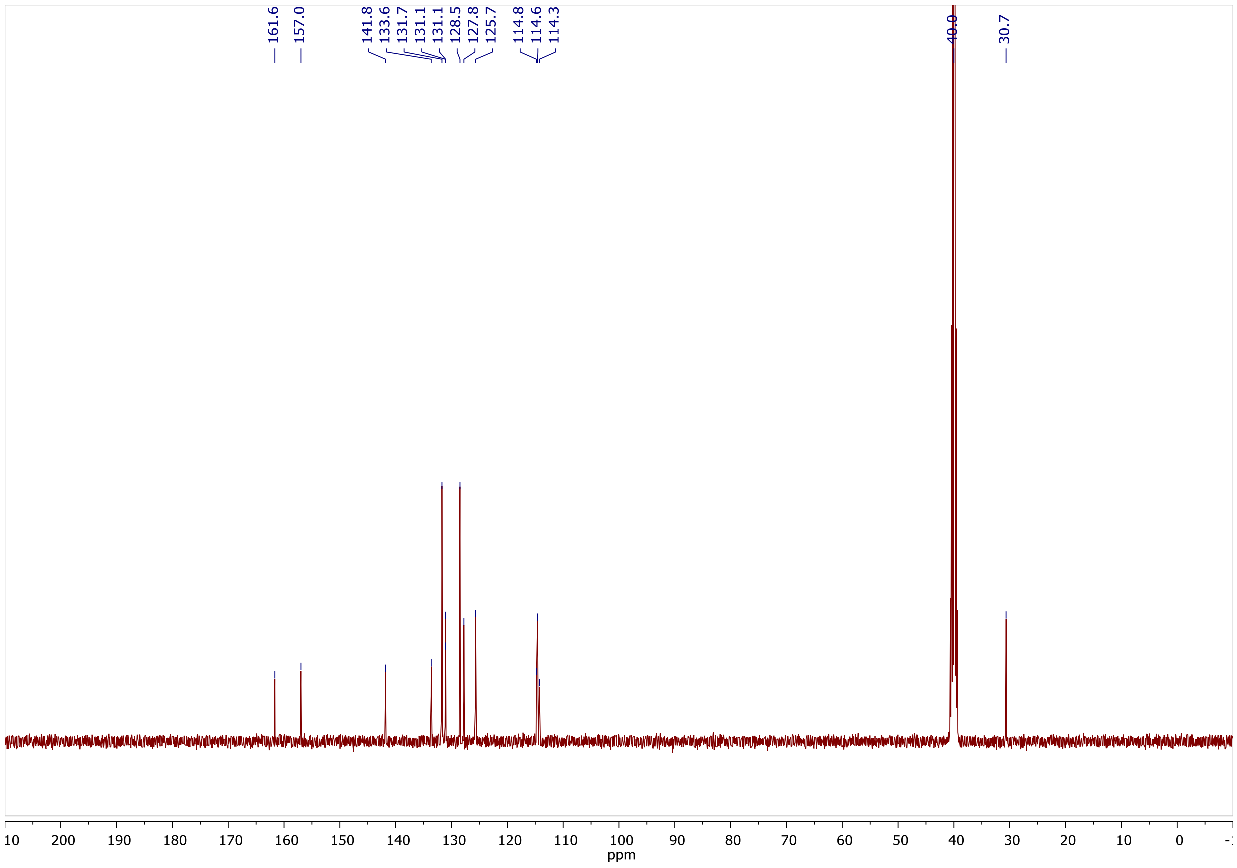

$^{1}$H NMR (400 MHz, DMSO-d$_6$) spectrum of compound **1i**

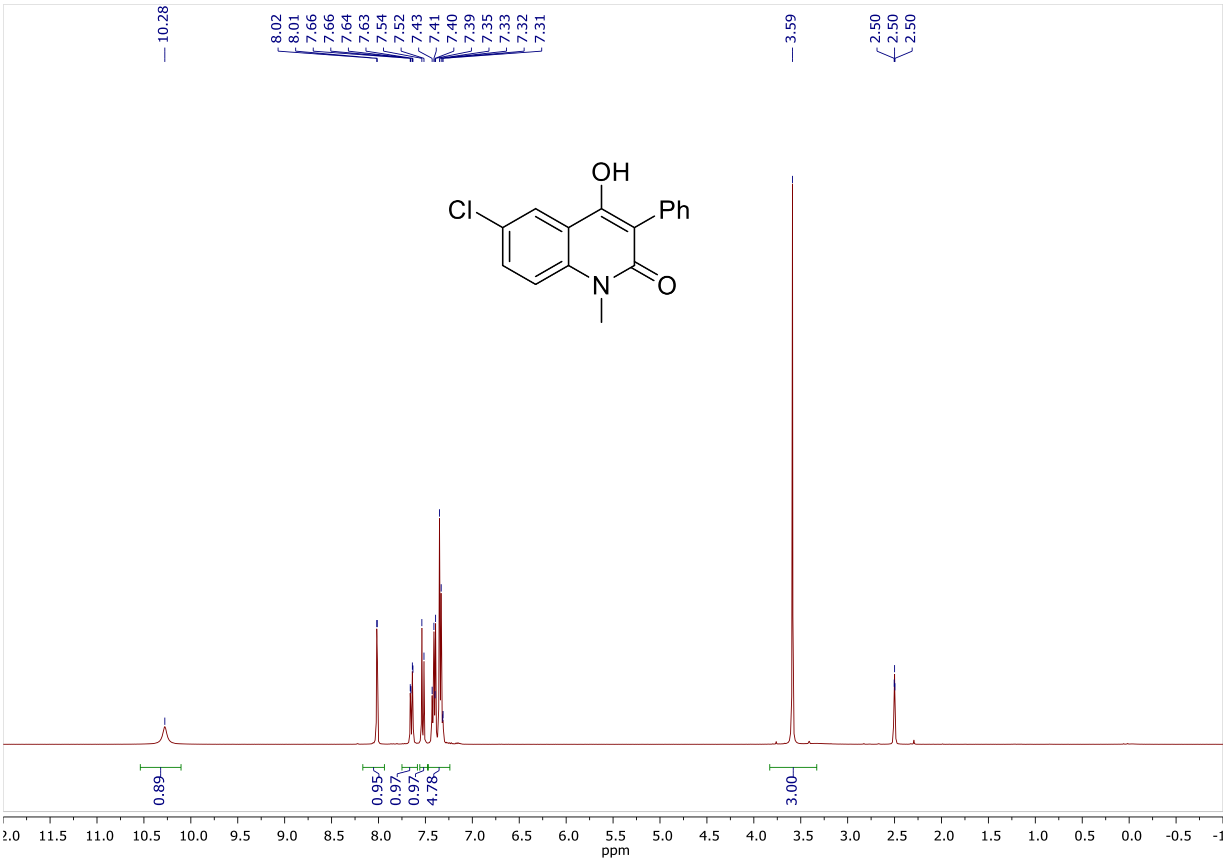


$^{13}$C NMR (100 MHz, DMSO-d$_6$) spectrum of compound **1i**

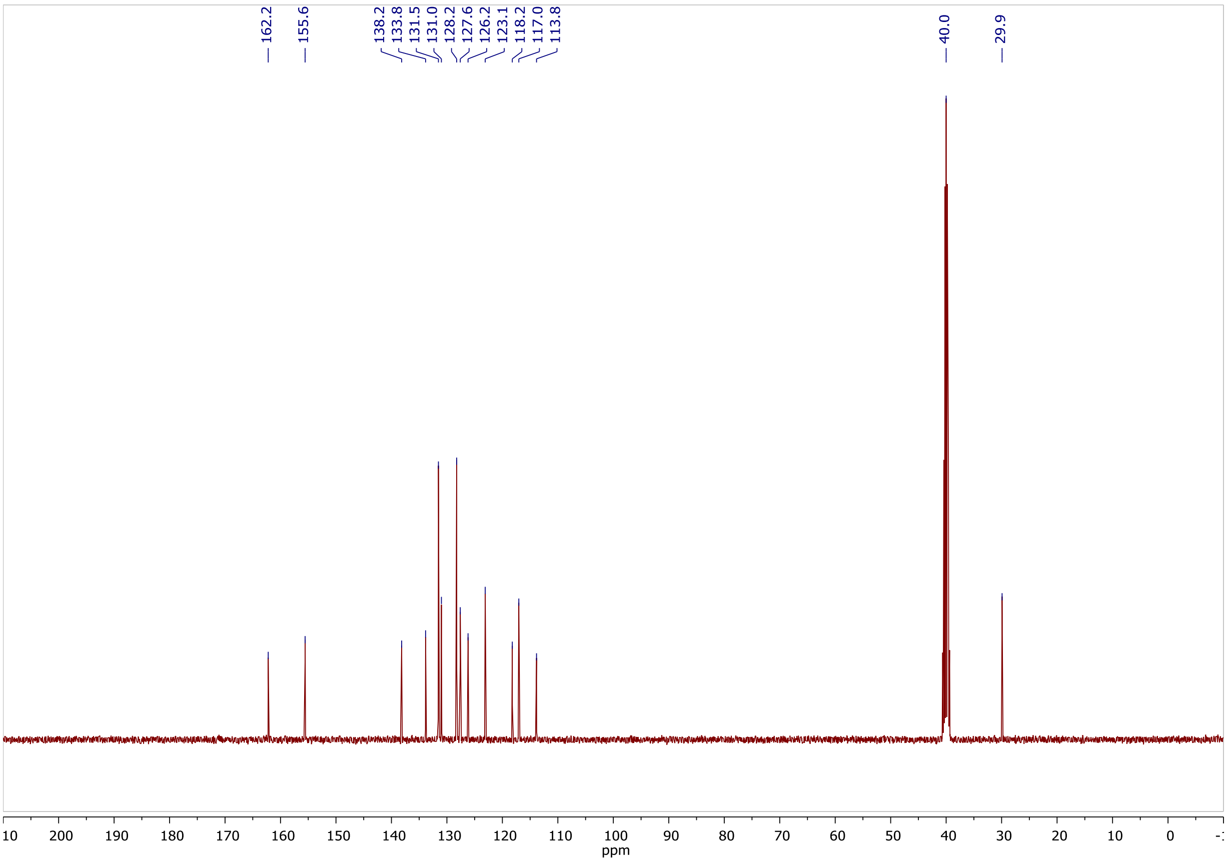

$^{1}H$ NMR (400 MHz, DMSO-$d_6$) spectrum of compound **1j**

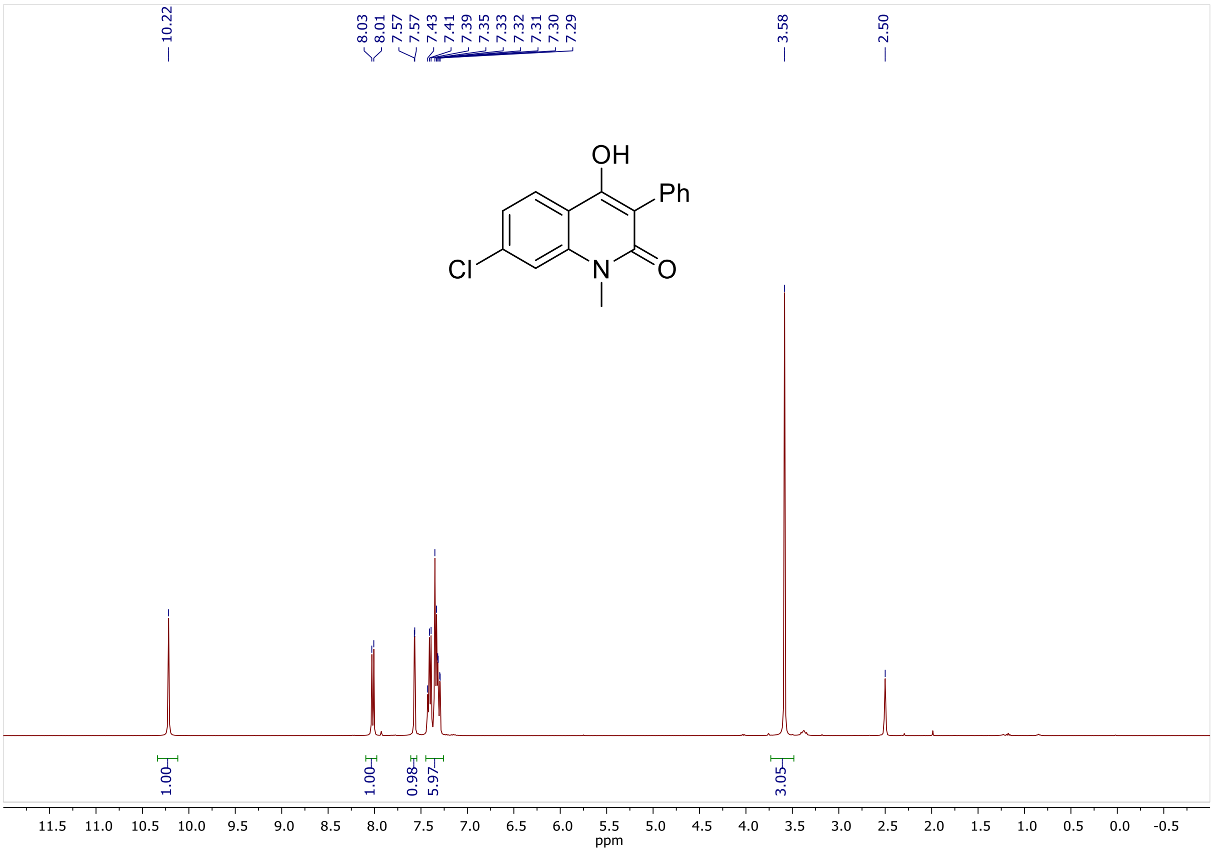


$^{13}C$ NMR (100 MHz, DMSO-$d_6$) spectrum of compound **1j**

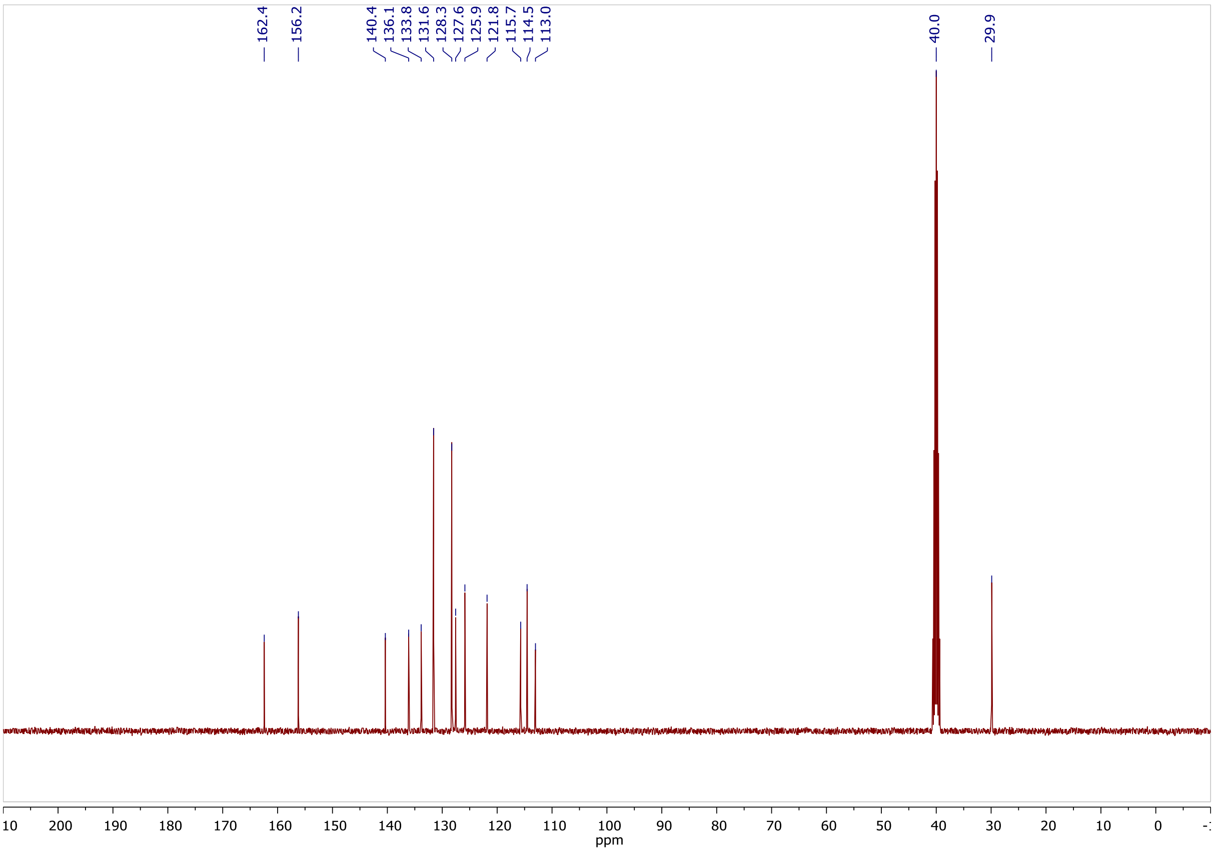

$^1$H NMR (400 MHz, DMSO-d$_6$) spectrum of compound **1k**

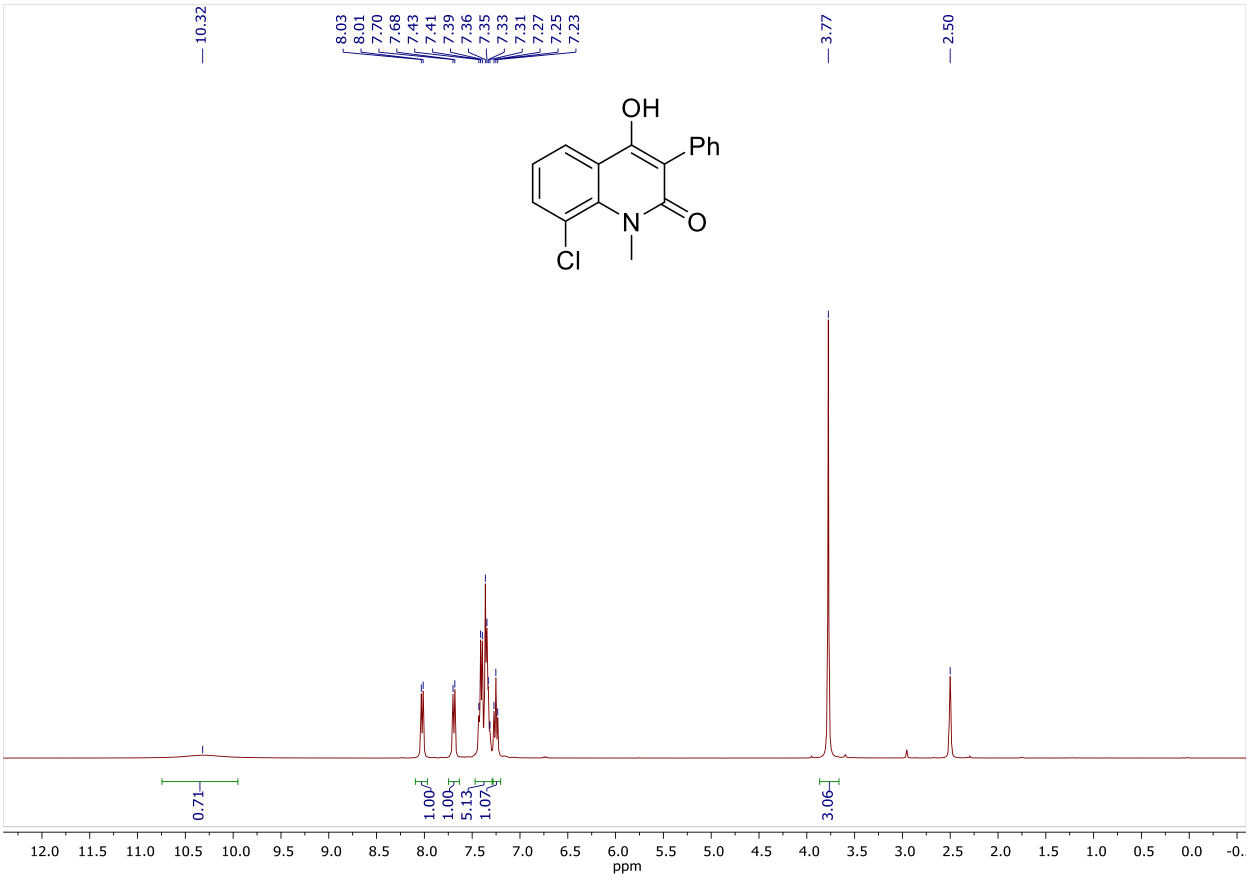


$^{13}$C NMR (100 MHz, DMSO-d$_6$) spectrum of compound **1k**

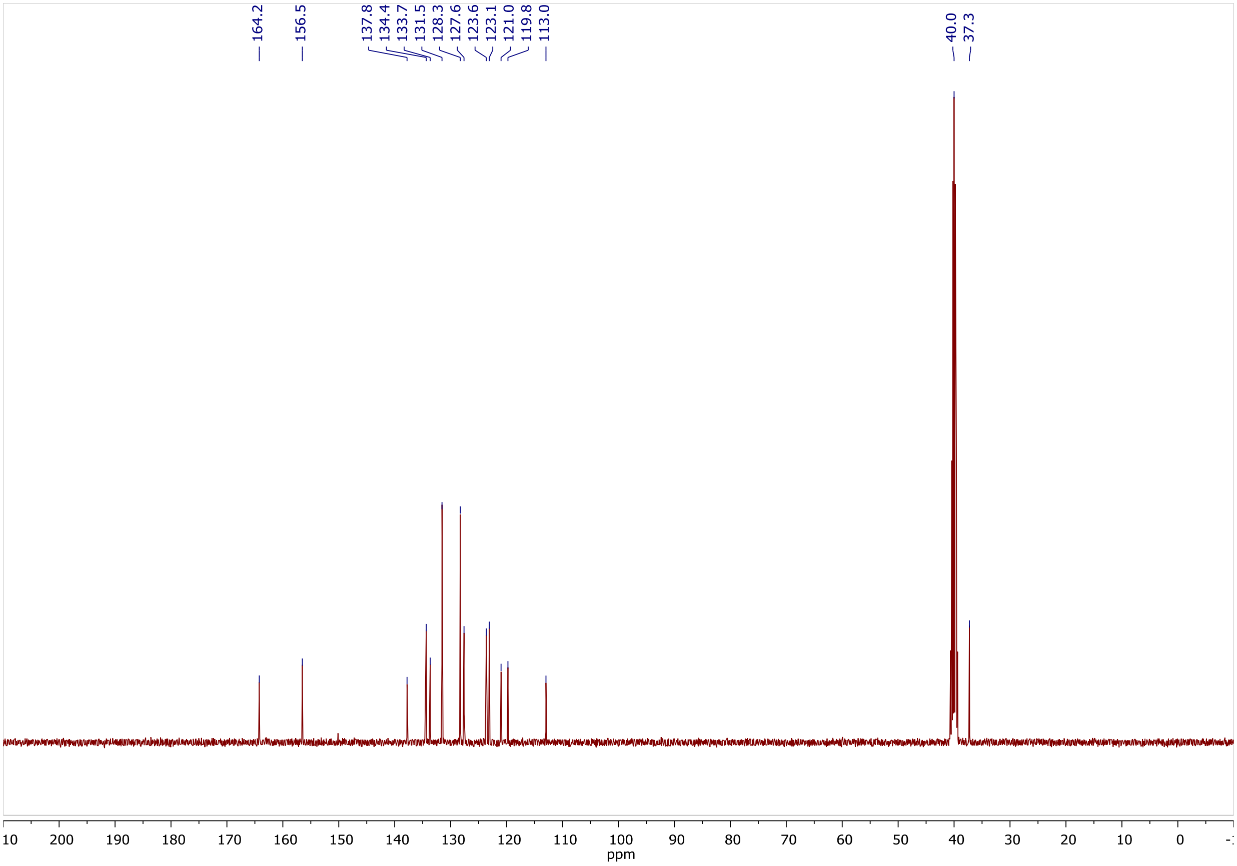

$^{1}$H NMR (400 MHz, DMSO-d$_6$) spectrum of compound **3a**

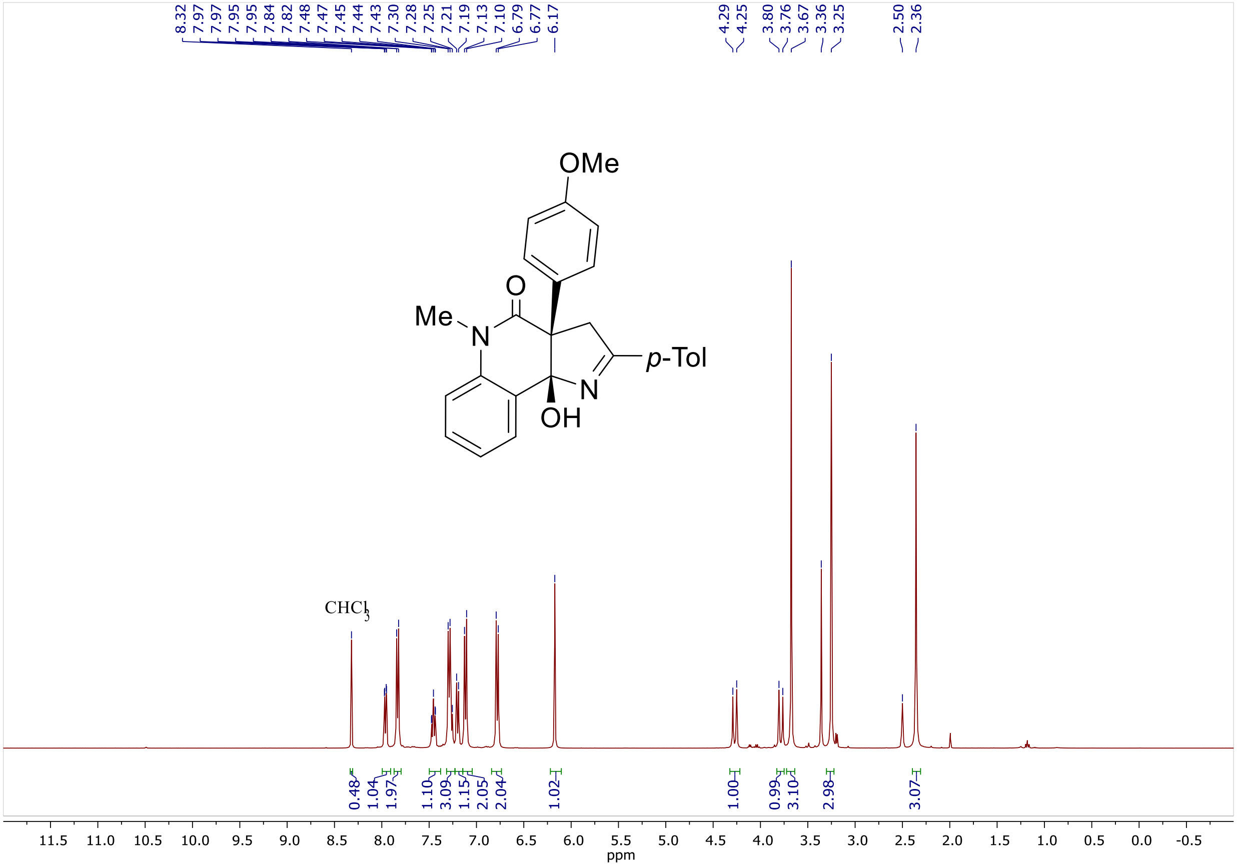


$^{13}$C NMR (100 MHz, DMSO-d$_6$) spectrum of compound **3a**

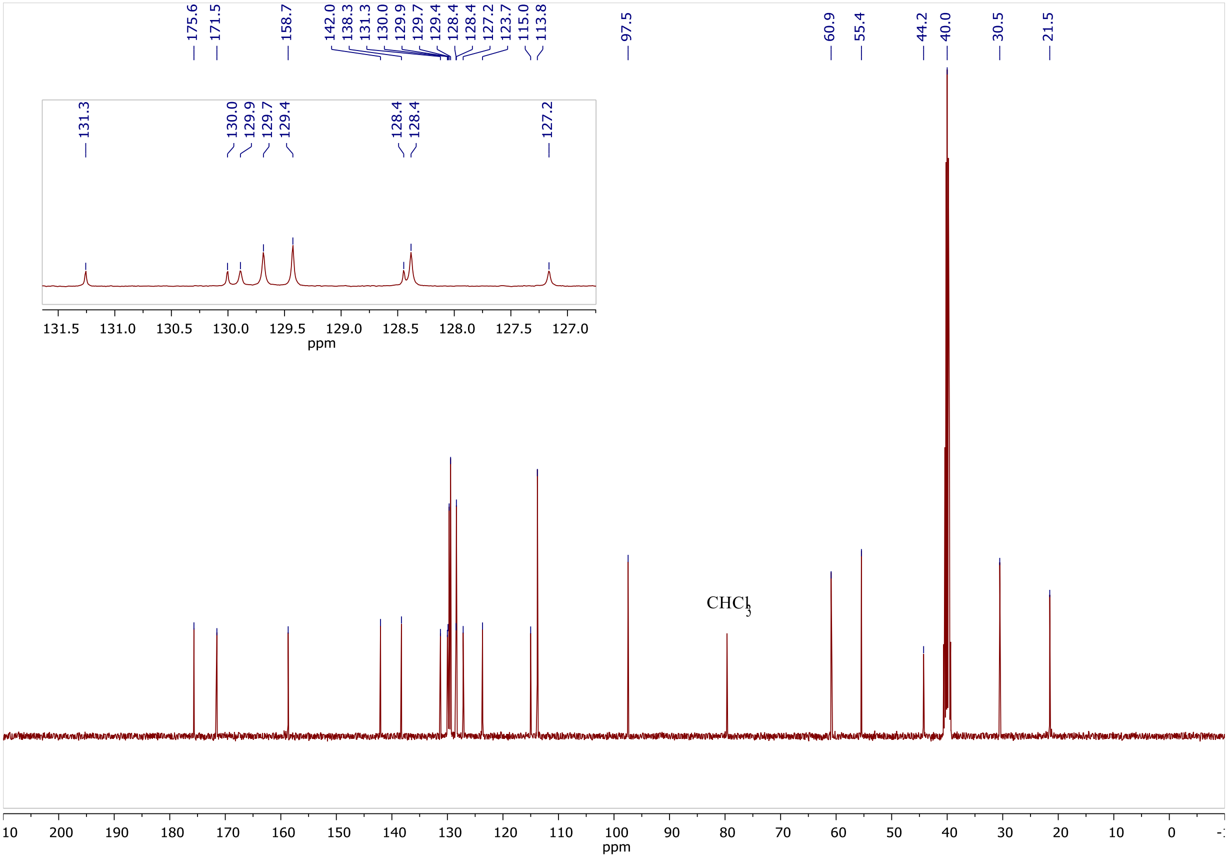

$^{1}$H NMR (400 MHz, DMSO-d$_6$) spectrum of compound **3b**

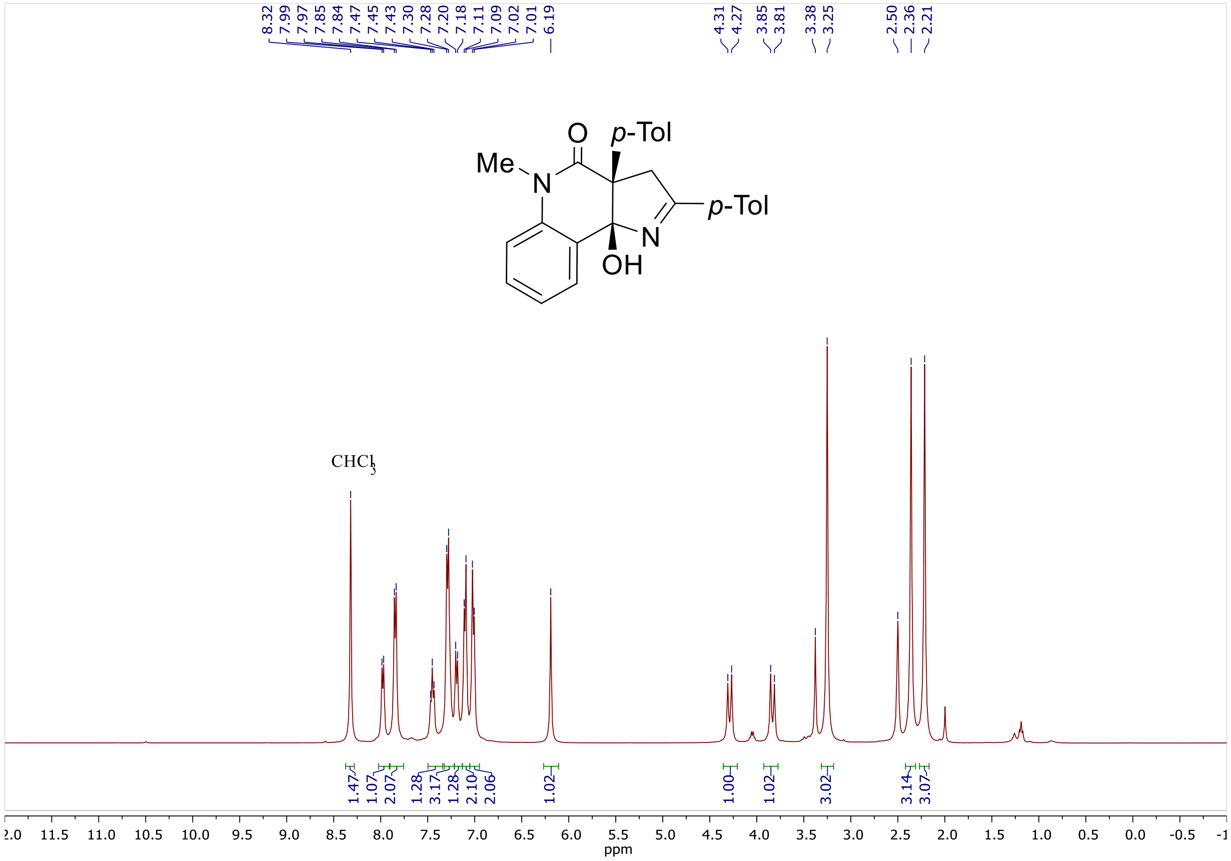

$^{13}$C NMR (100 MHz, DMSO-d$_6$) spectrum of compound **3b**

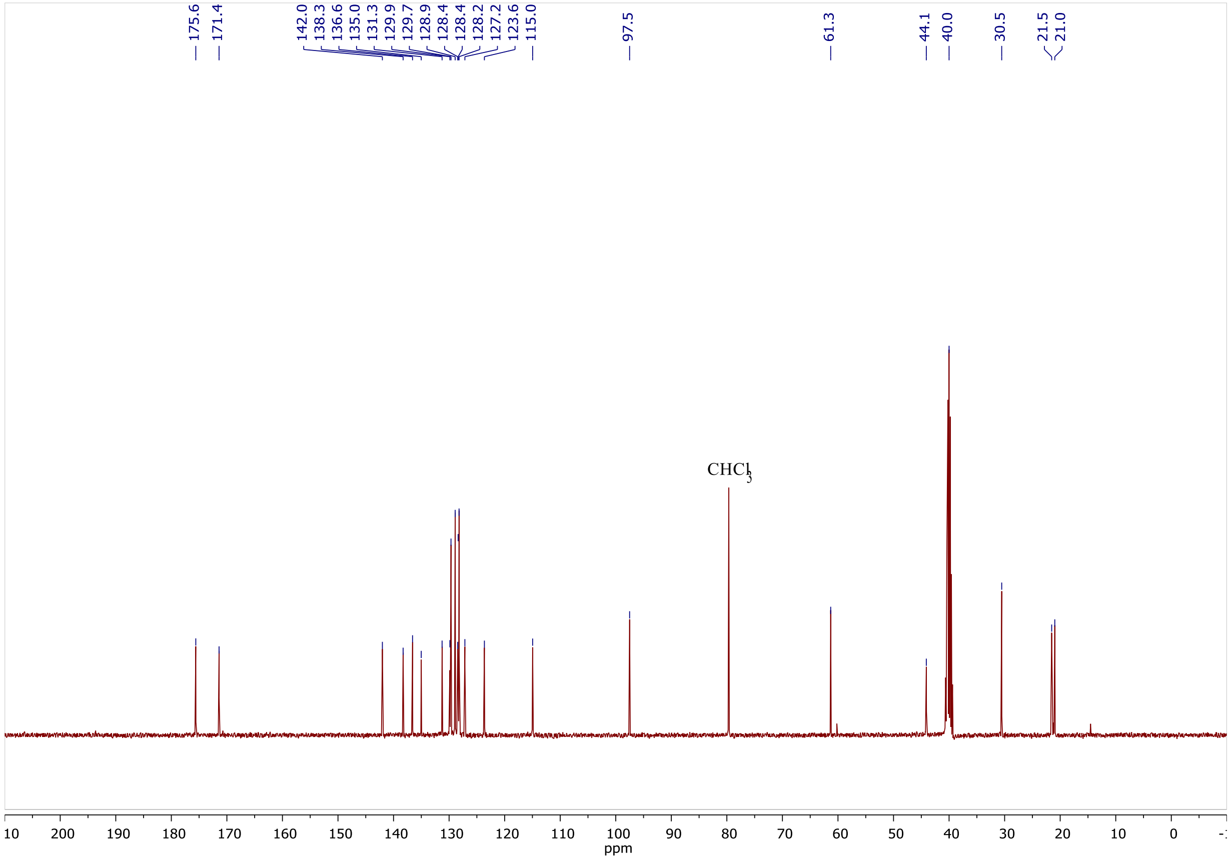

$^{1}H$ NMR (400 MHz, $CDCl_3$) spectrum of compound **3c**

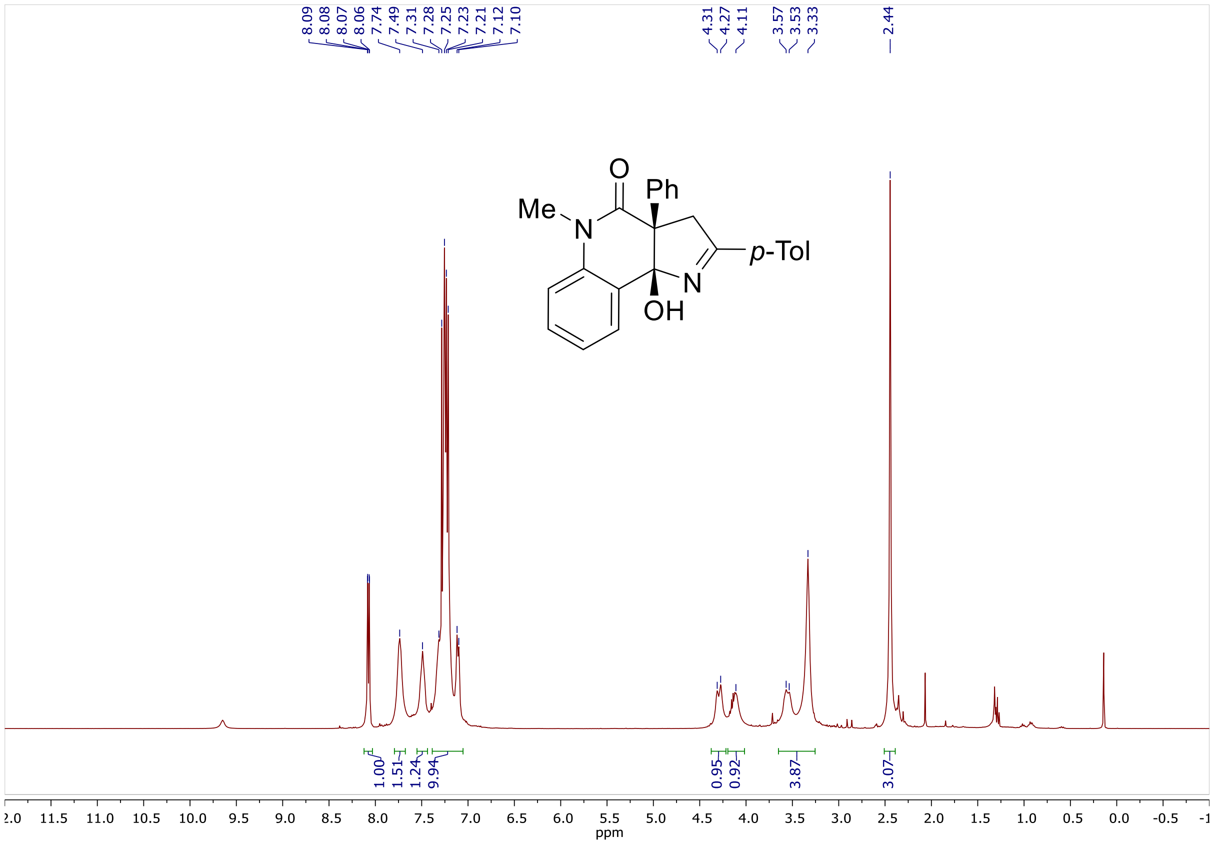

$^{13}C$ NMR (100 MHz, $CDCl_3$) spectrum of compound **3c**

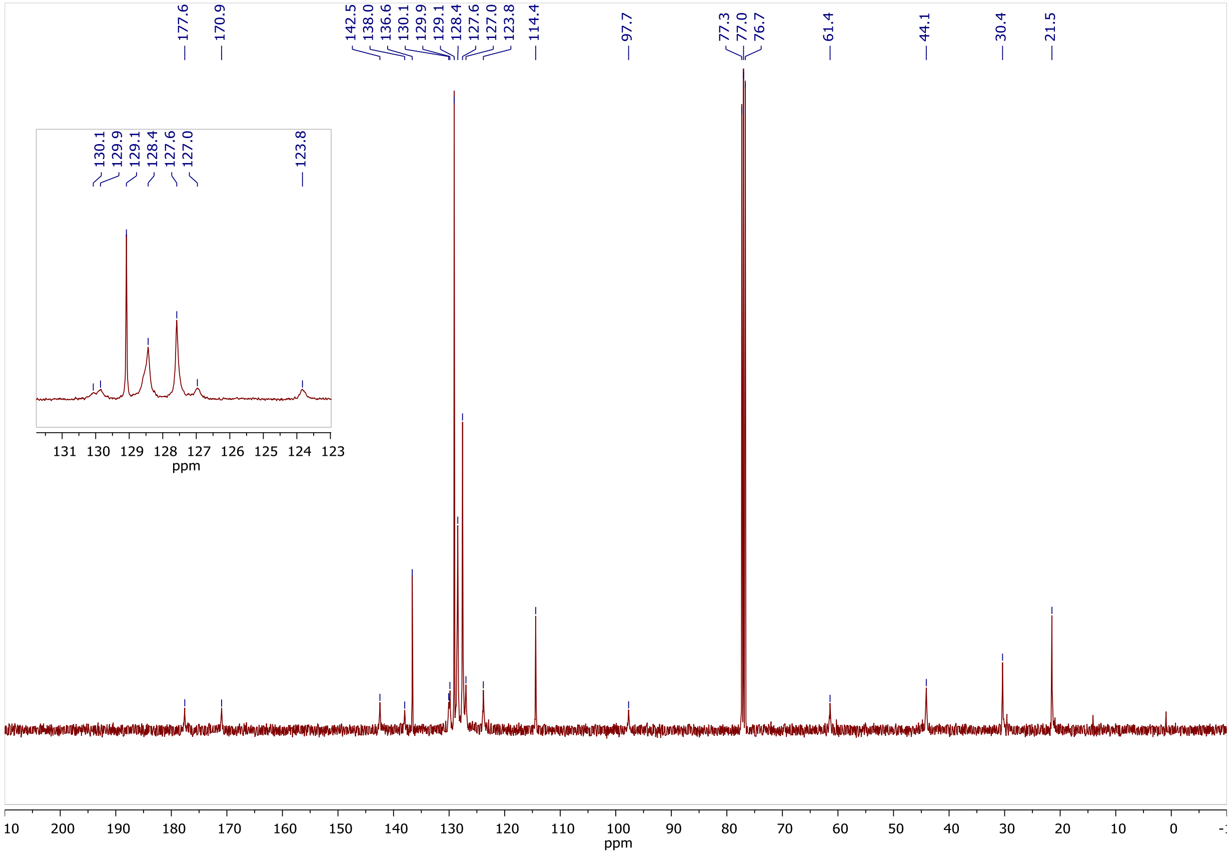

$^{1}H$ NMR (400 MHz, DMSO-d$_6$) spectrum of compound **3d**

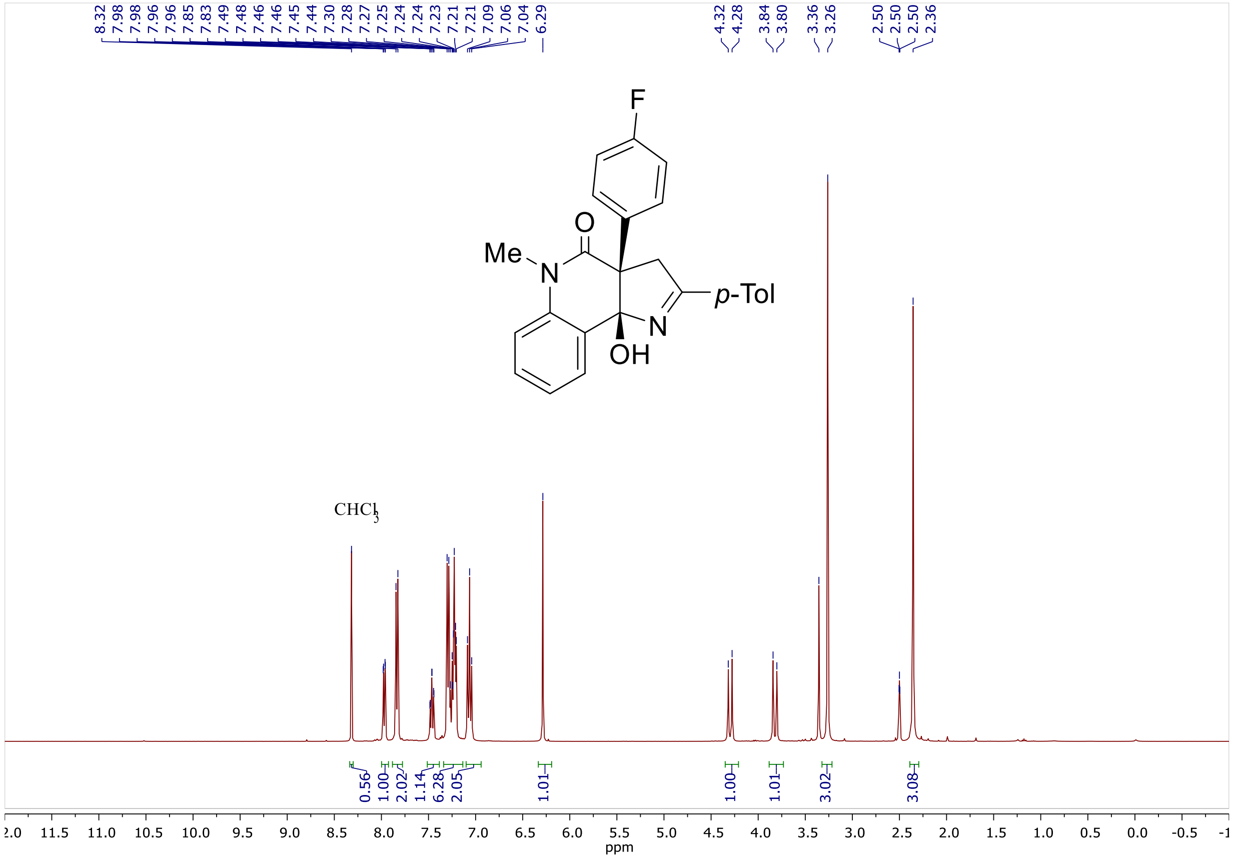

$^{13}C$ NMR (100 MHz, DMSO-d$_6$) spectrum of compound **3d**

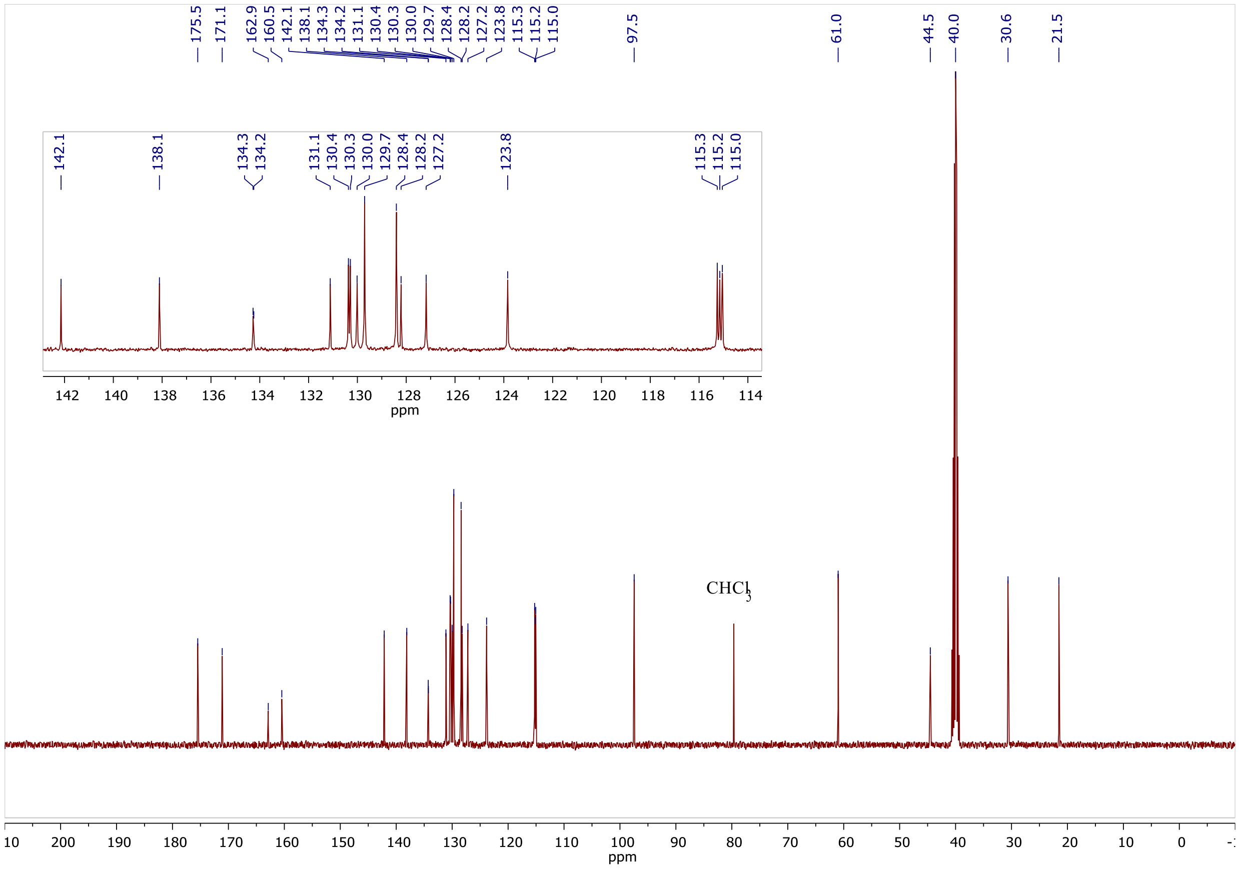

$^{1}$H NMR (400 MHz, DMSO-d$_6$) spectrum of compound **3e**

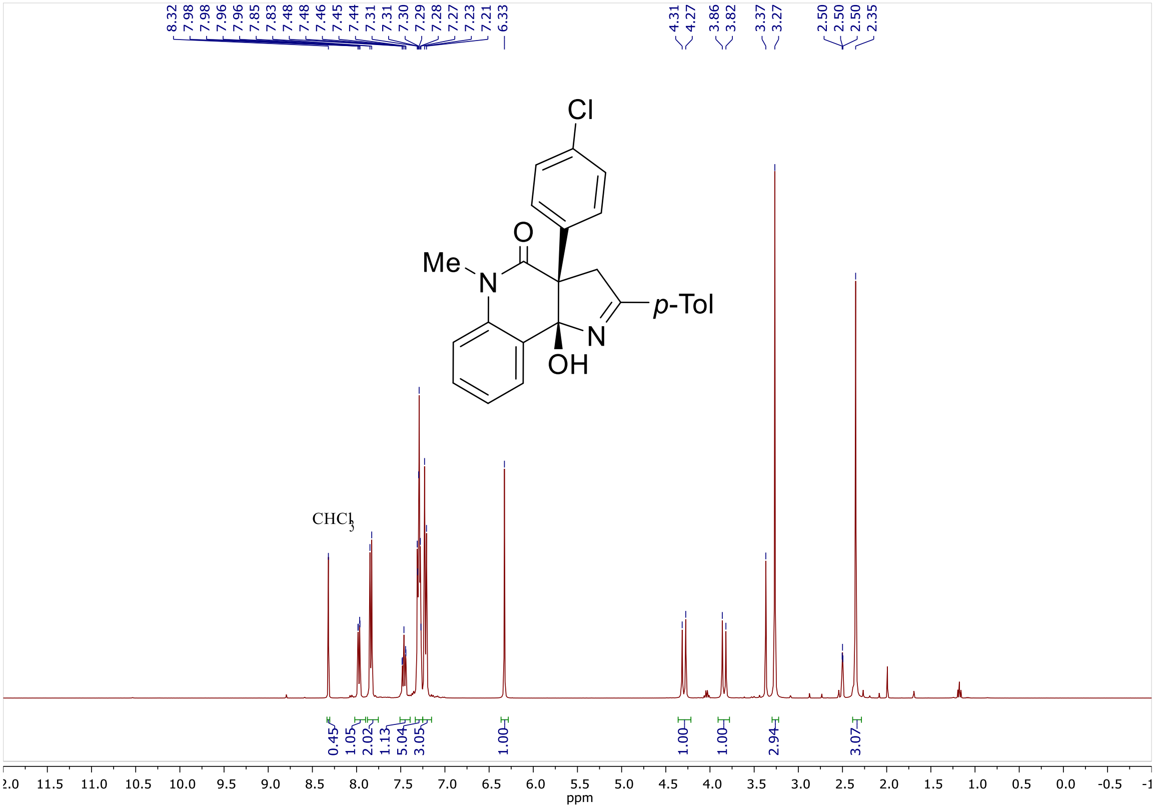

$^{13}$C NMR (100 MHz, DMSO-d$_6$) spectrum of compound **3e**

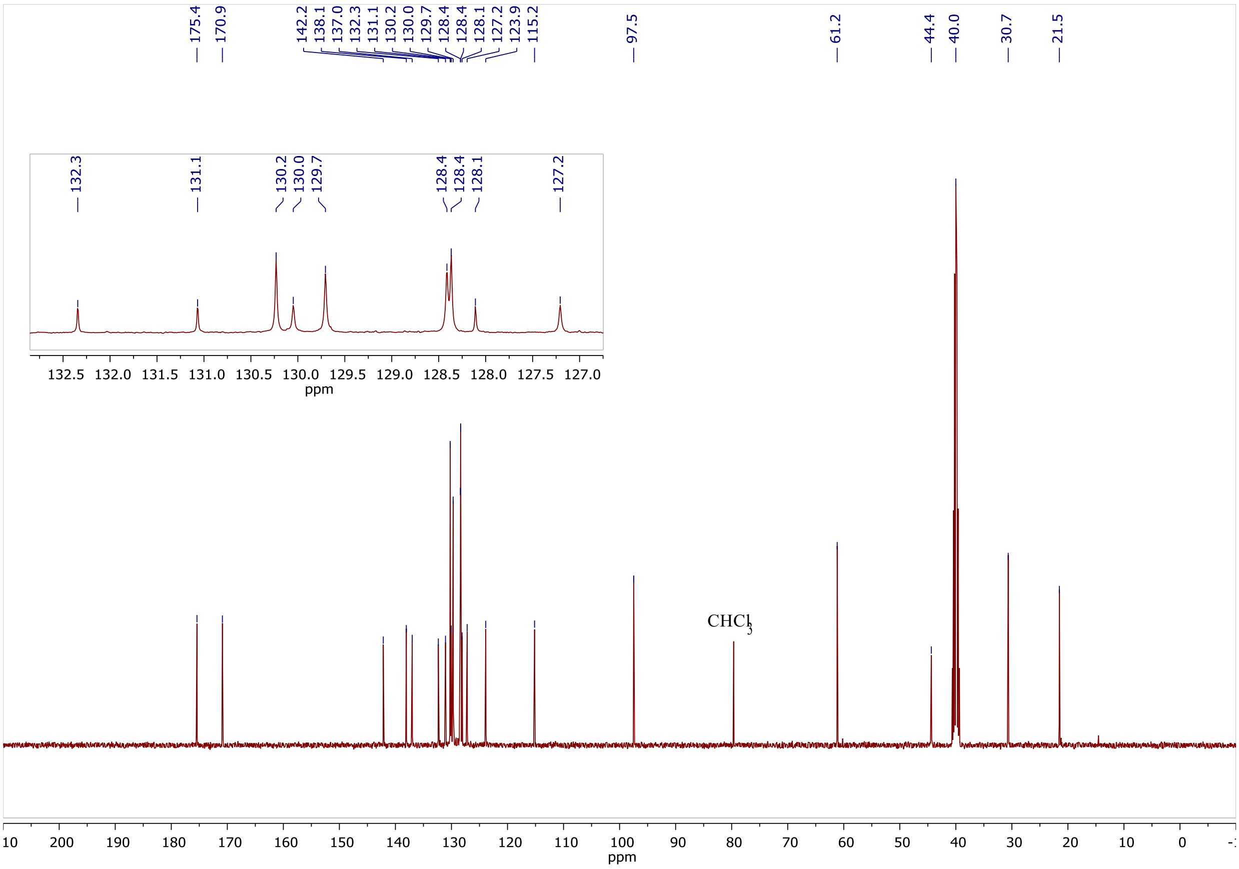

$^{1}H$ NMR (400 MHz, DMSO-$d_6$) spectrum of compound **3f**

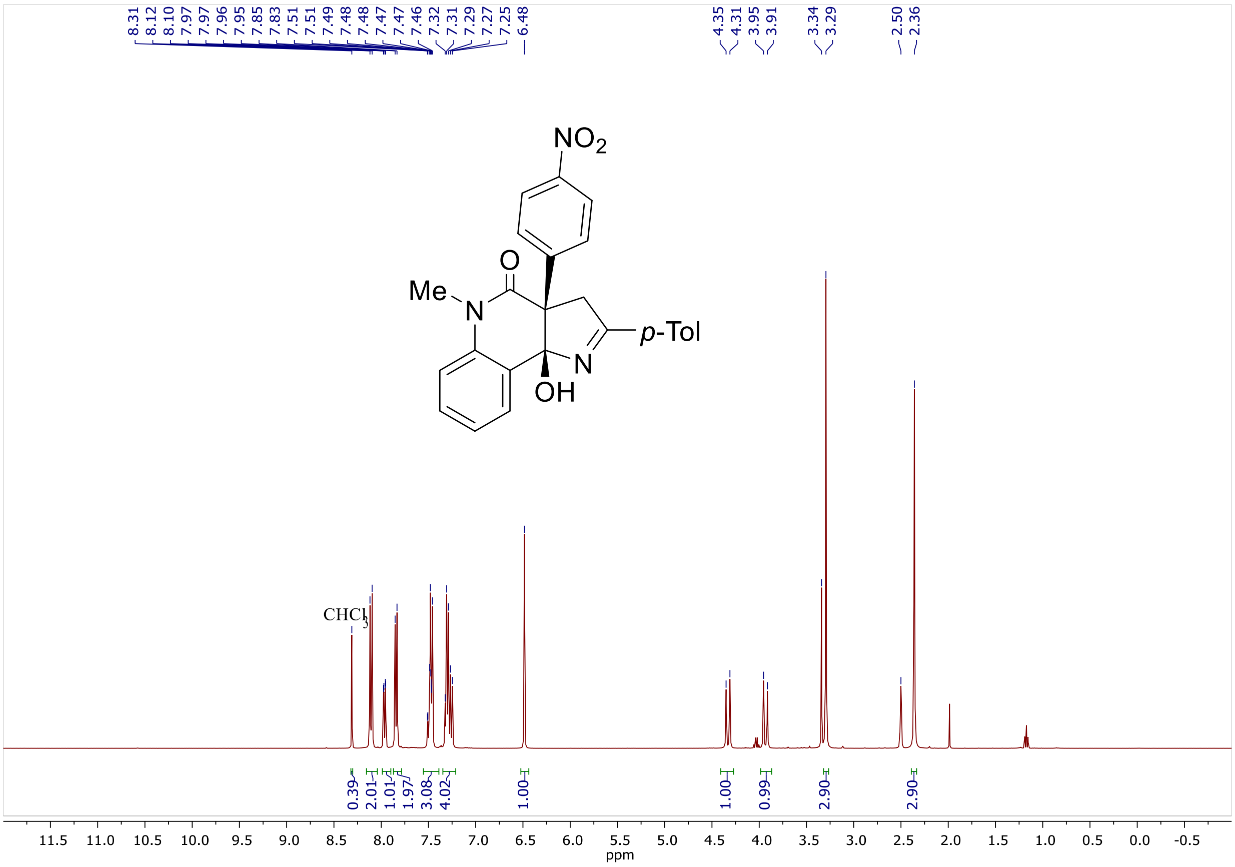


$^{13}C$ NMR (100 MHz, DMSO-$d_6$) spectrum of compound **3f**

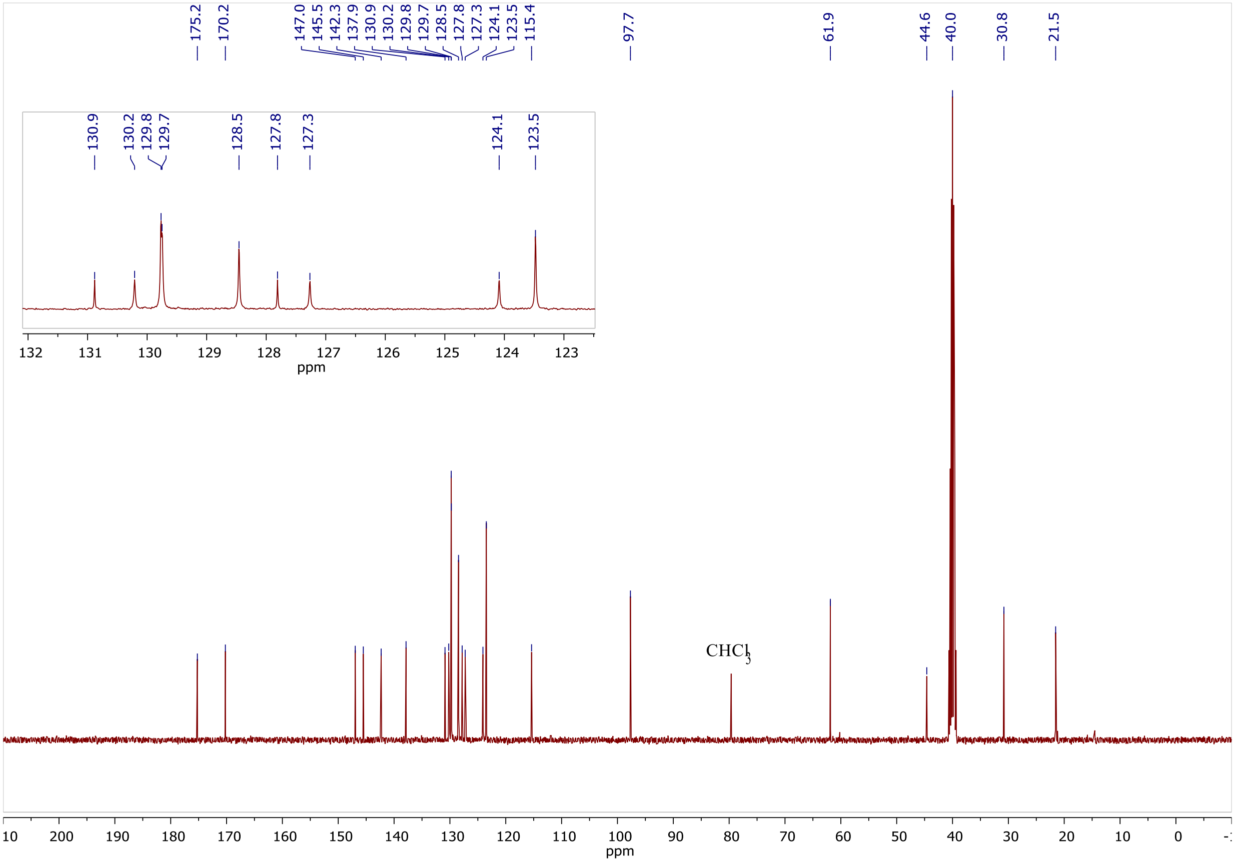

$^{1}H$ NMR (400 MHz, DMSO-$d_6$) spectrum of compound **3g**

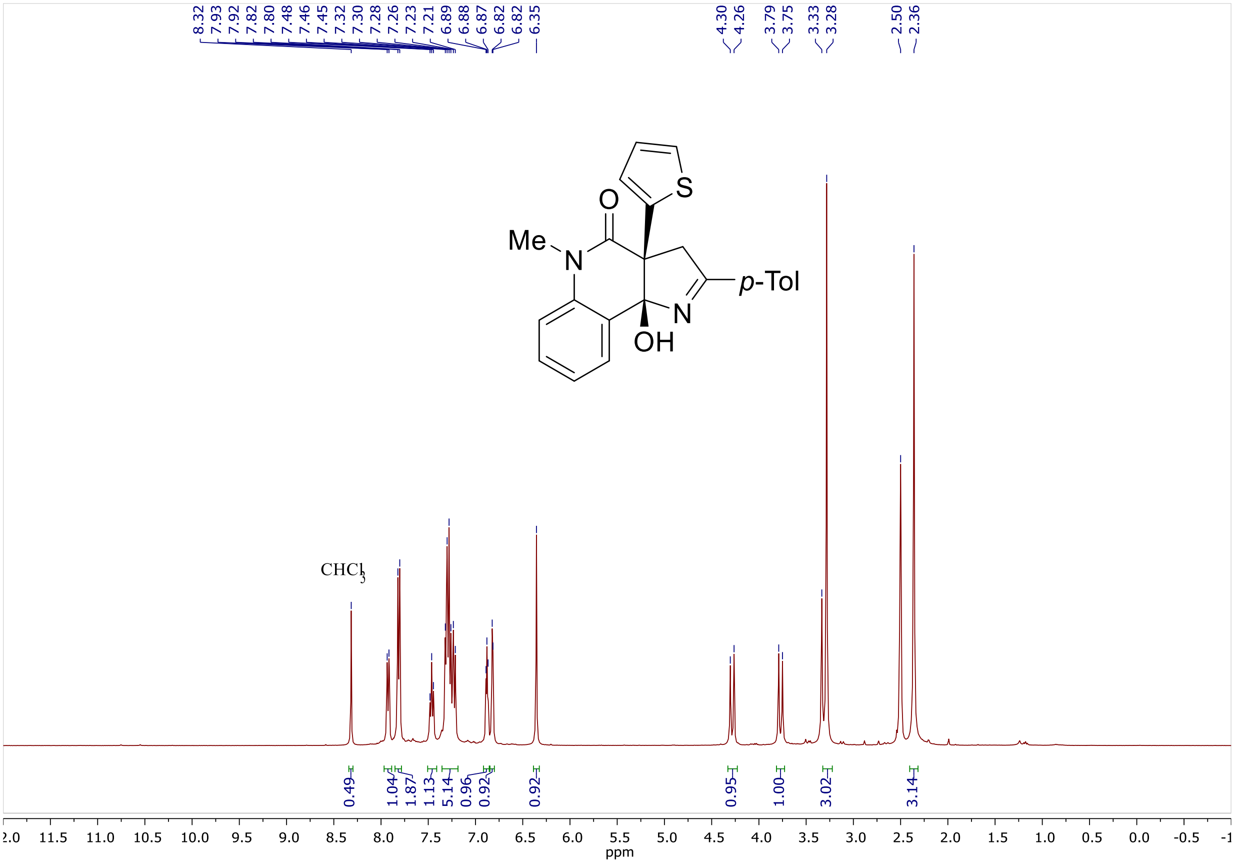


$^{13}C$ NMR (100 MHz, DMSO-$d_6$) spectrum of compound **3g**

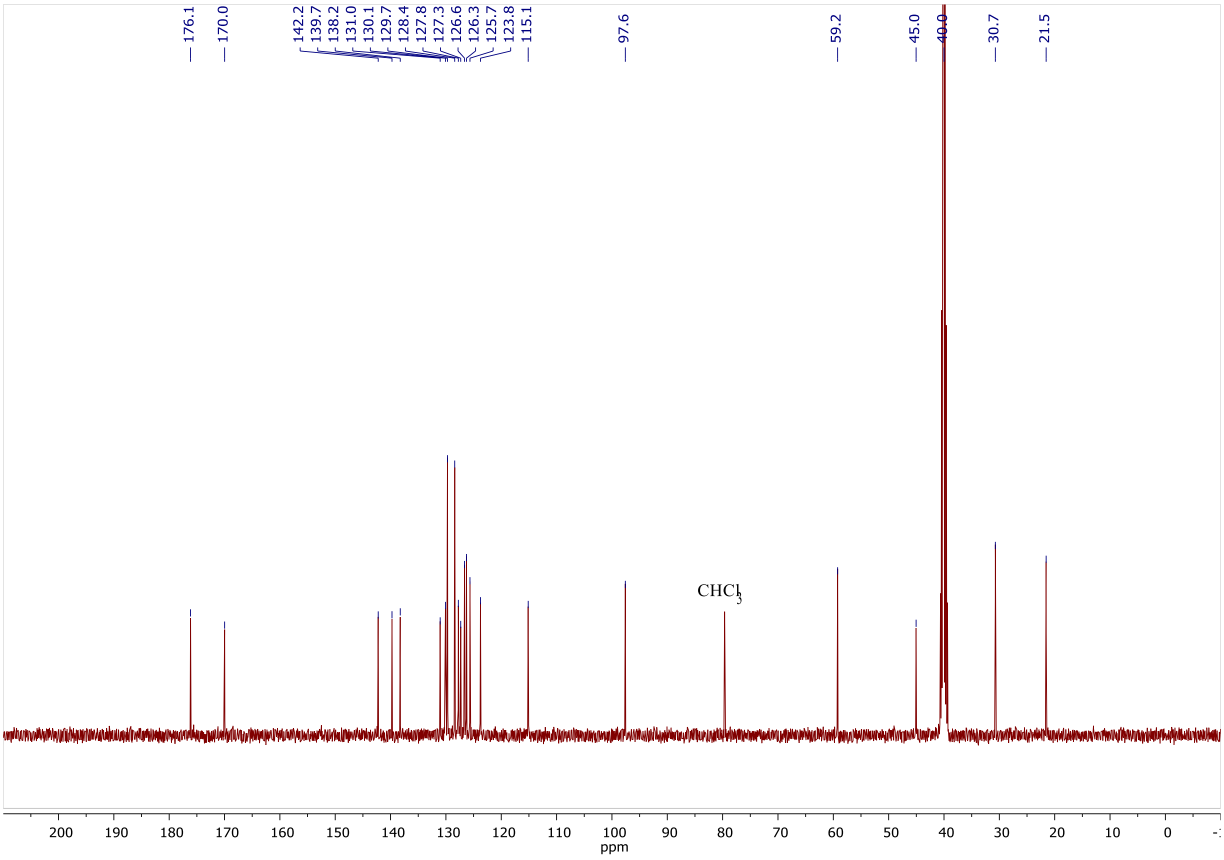

$^{1}$H NMR (400 MHz, DMSO-d$_6$) spectrum of compound **3h**

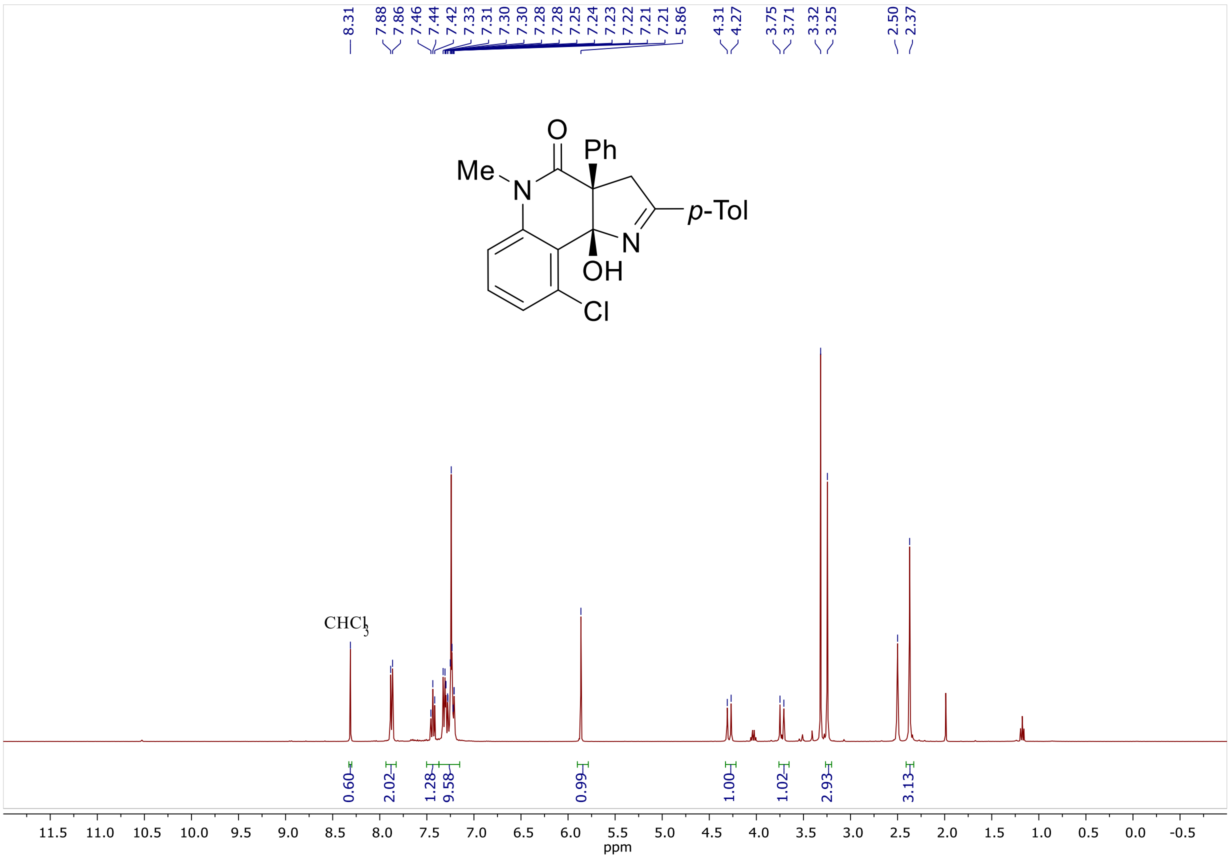


$^{13}$C NMR (100 MHz, DMSO-d$_6$) spectrum of compound **3h**

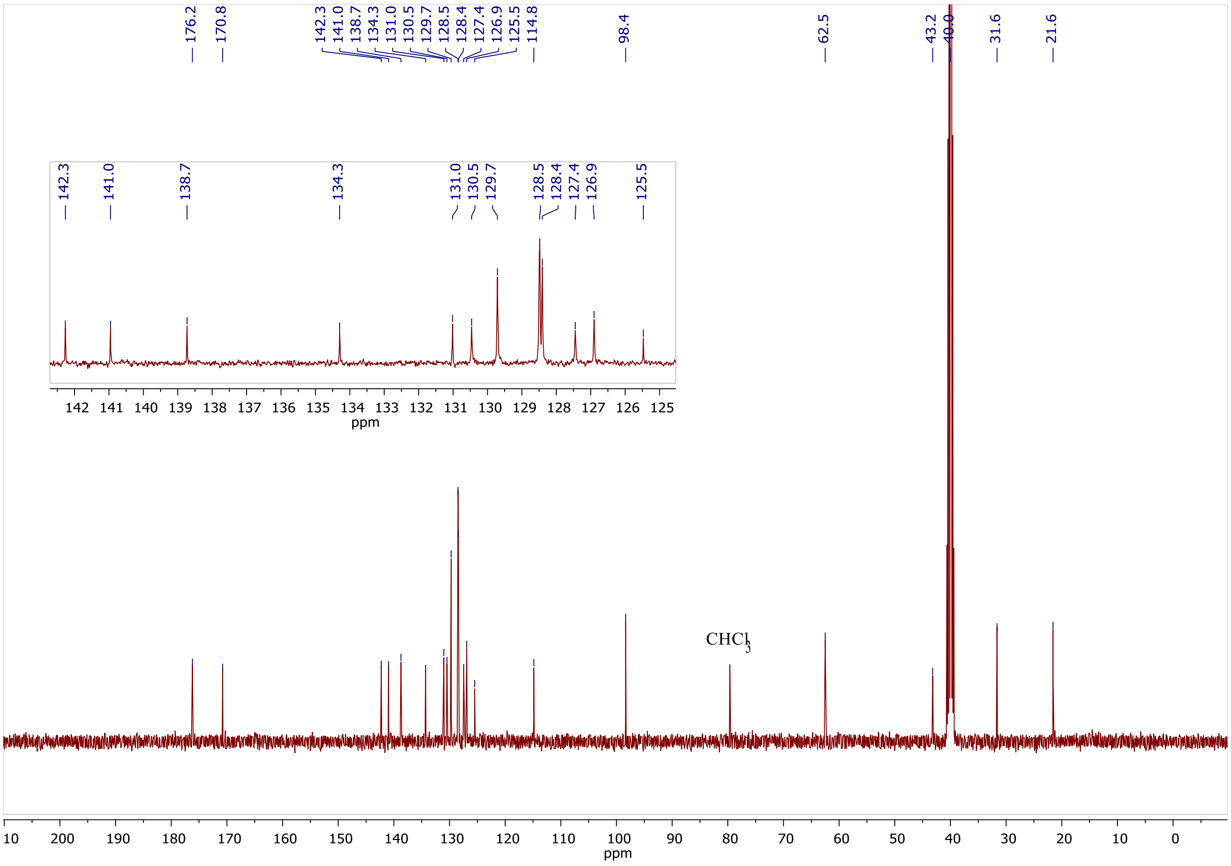

$^{1}$H NMR (400 MHz, DMSO-$d_6$) spectrum of compound **3i**

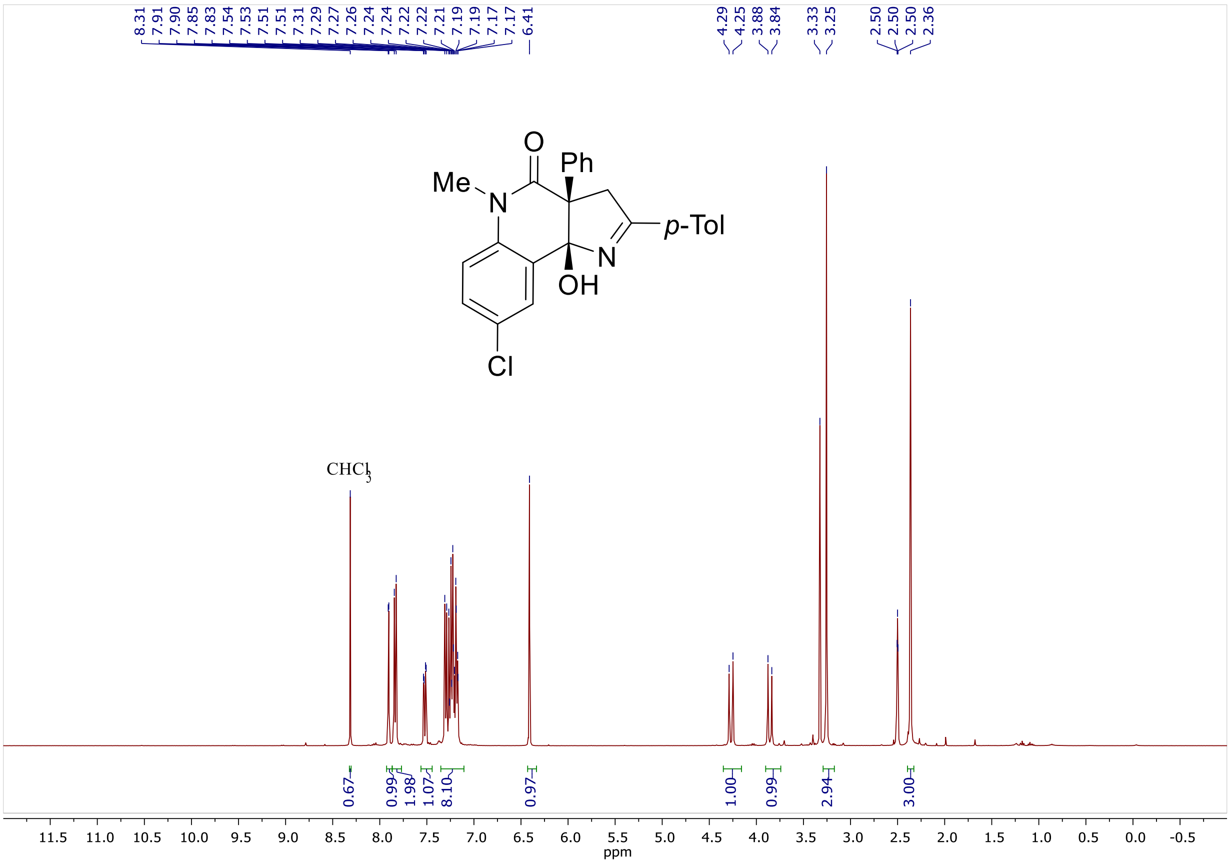

$^{13}$C NMR (100 MHz, DMSO-$d_6$) spectrum of compound **3i**

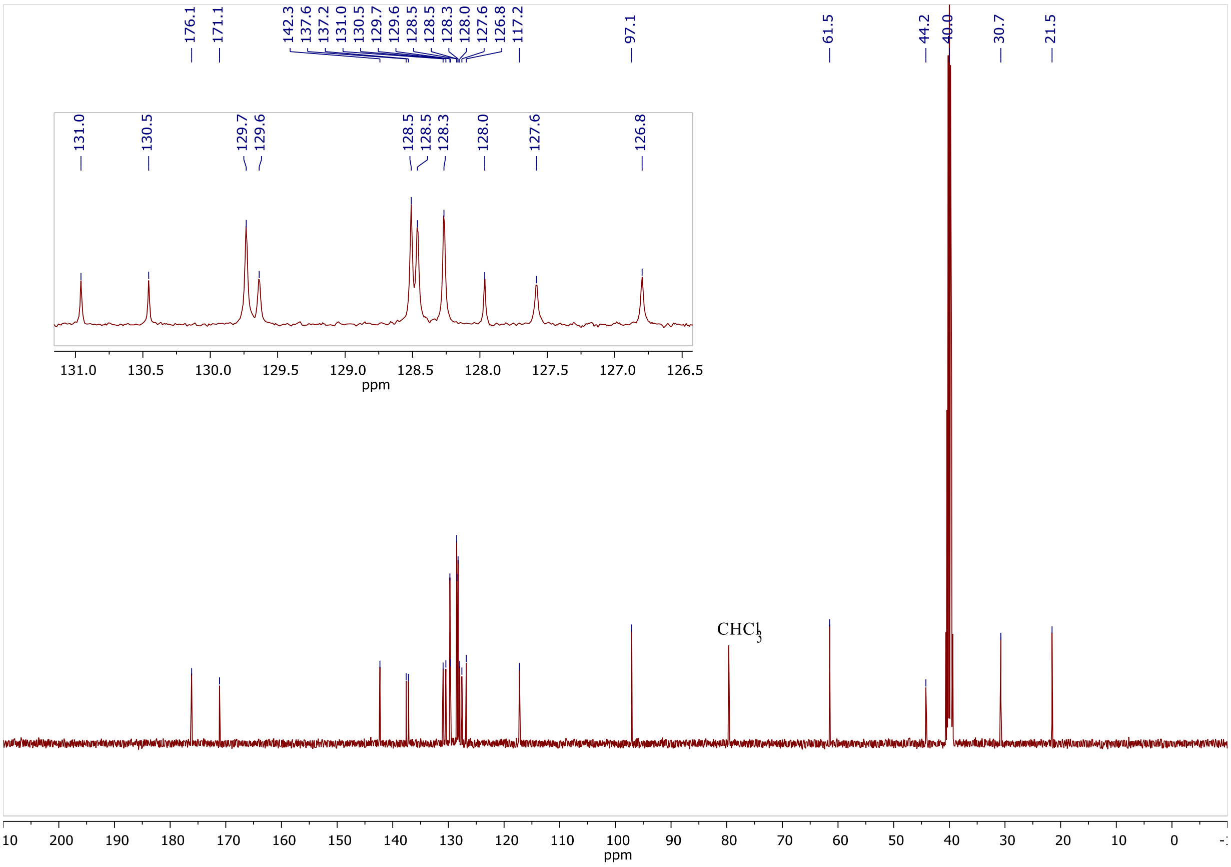

$^{1}H$ NMR (400 MHz, DMSO-d$_6$) spectrum of compound **3i** prepared by evaporation of DMSO-d$_6$ solution of solvate **3i** with $CHCl_3$

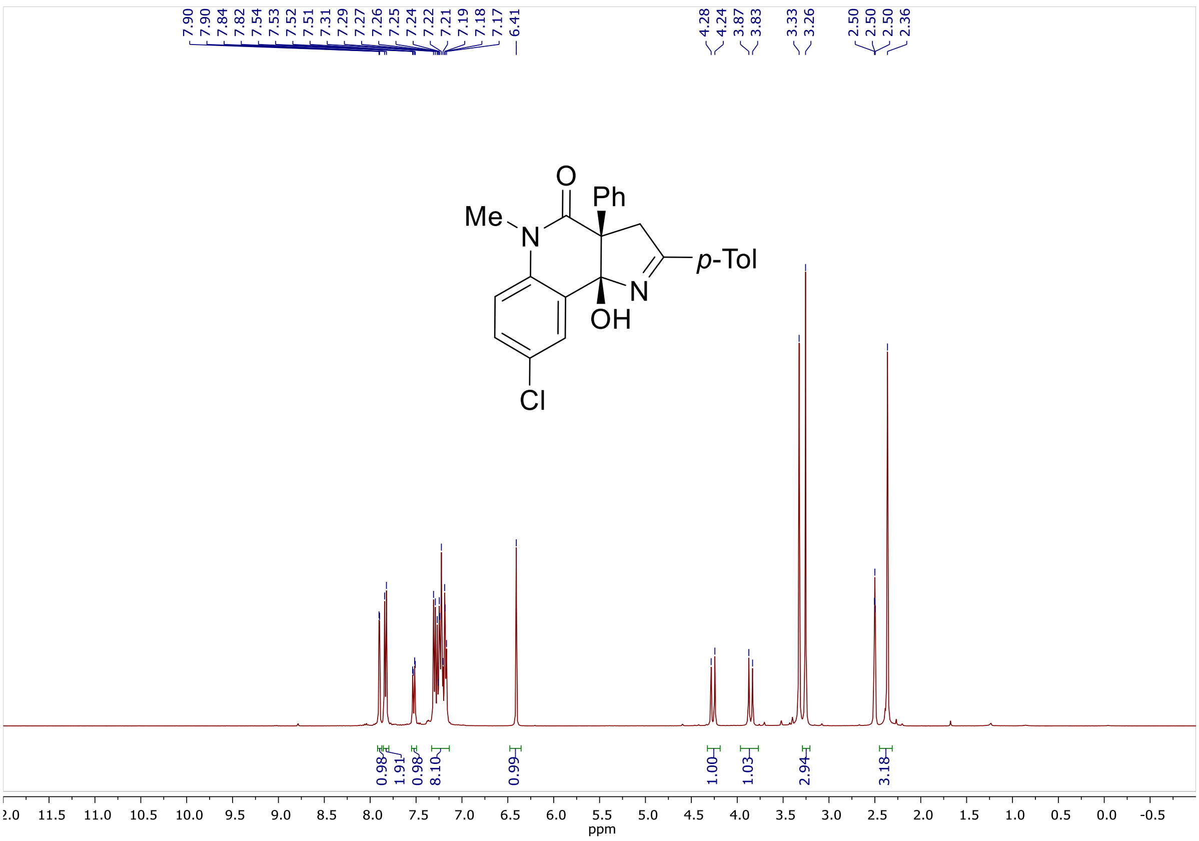


$^{13}C$ NMR (100 MHz, DMSO-d$_6$) spectrum of compound **3i** prepared by evaporation of DMSO-d$_6$ solution of solvate **3i** with $CHCl_3$

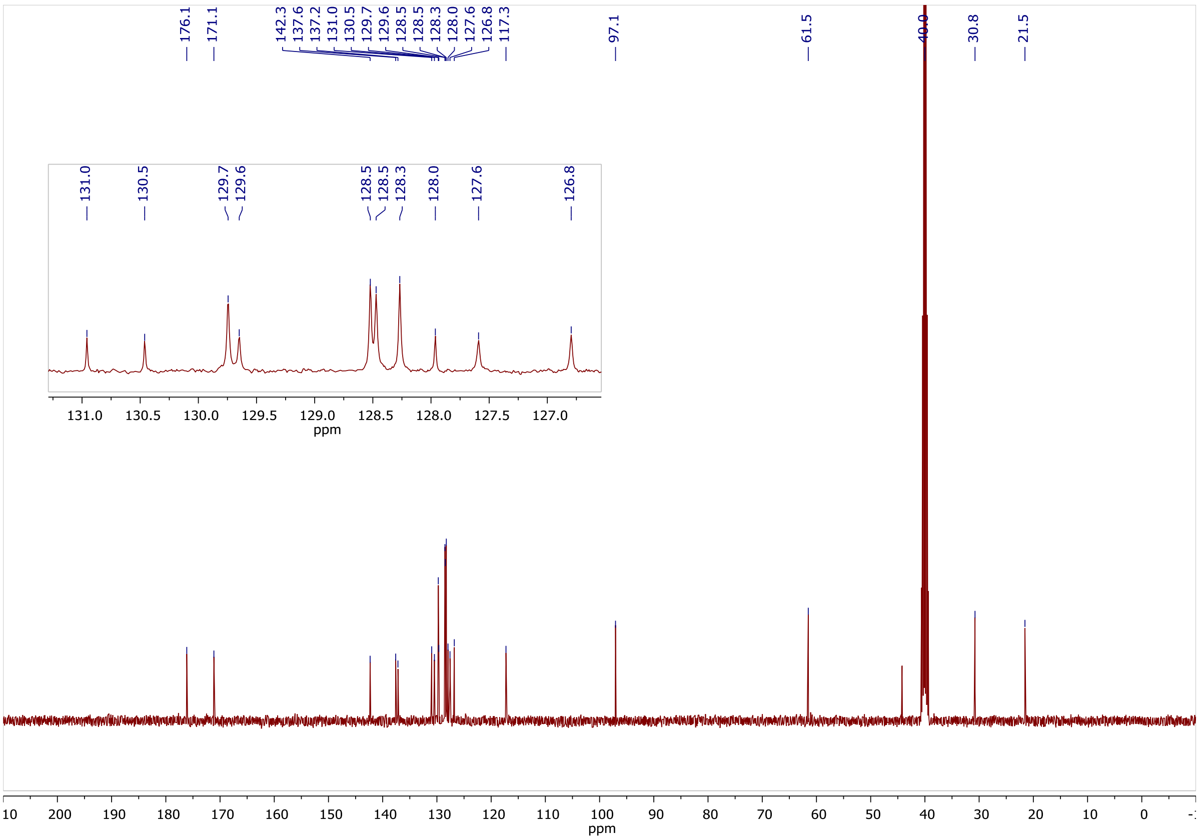

$^{1}H$ NMR (400 MHz, DMSO-d$_6$) spectrum of compound **3j**

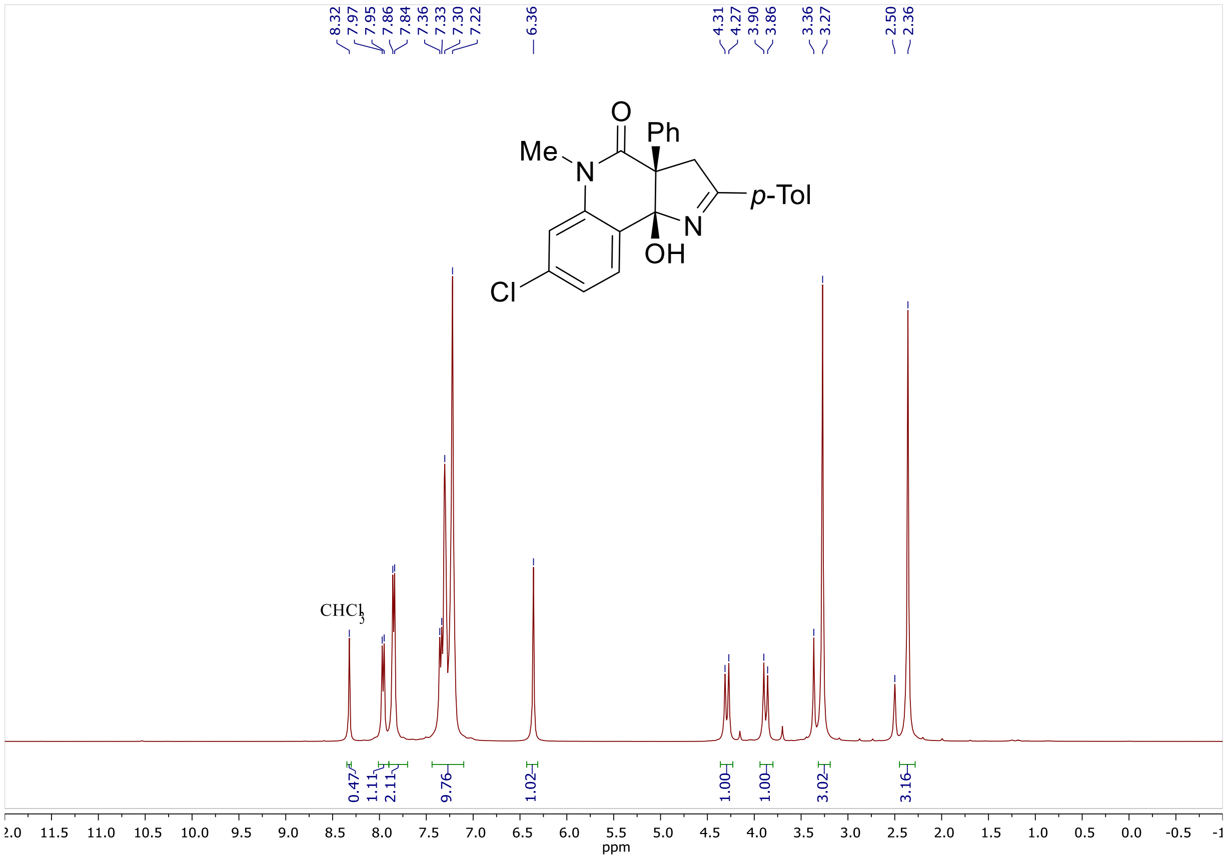


$^{13}C$ NMR (100 MHz, DMSO-d$_6$) spectrum of compound **3j**

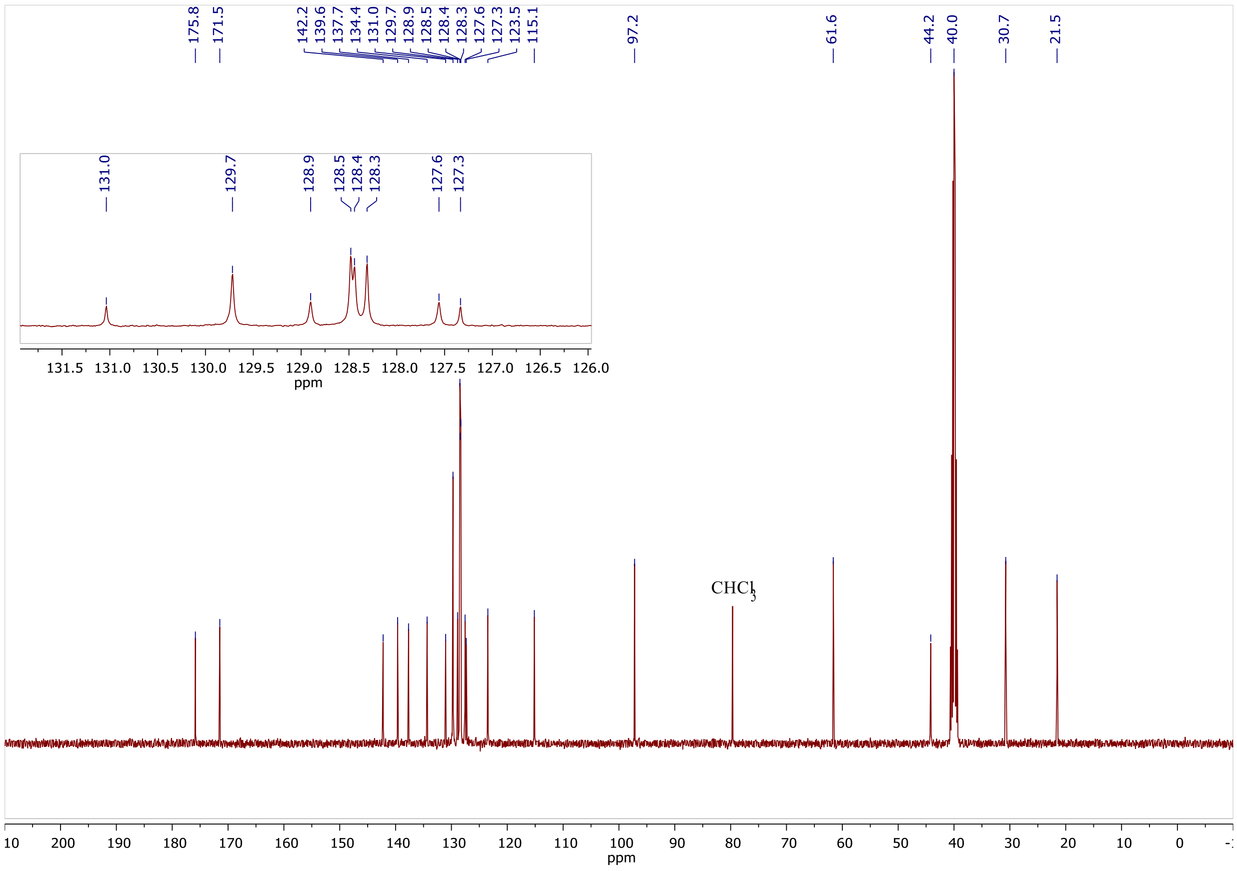

$^{1}H$ NMR (400 MHz, DMSO-d$_6$) spectrum of compound **3k**

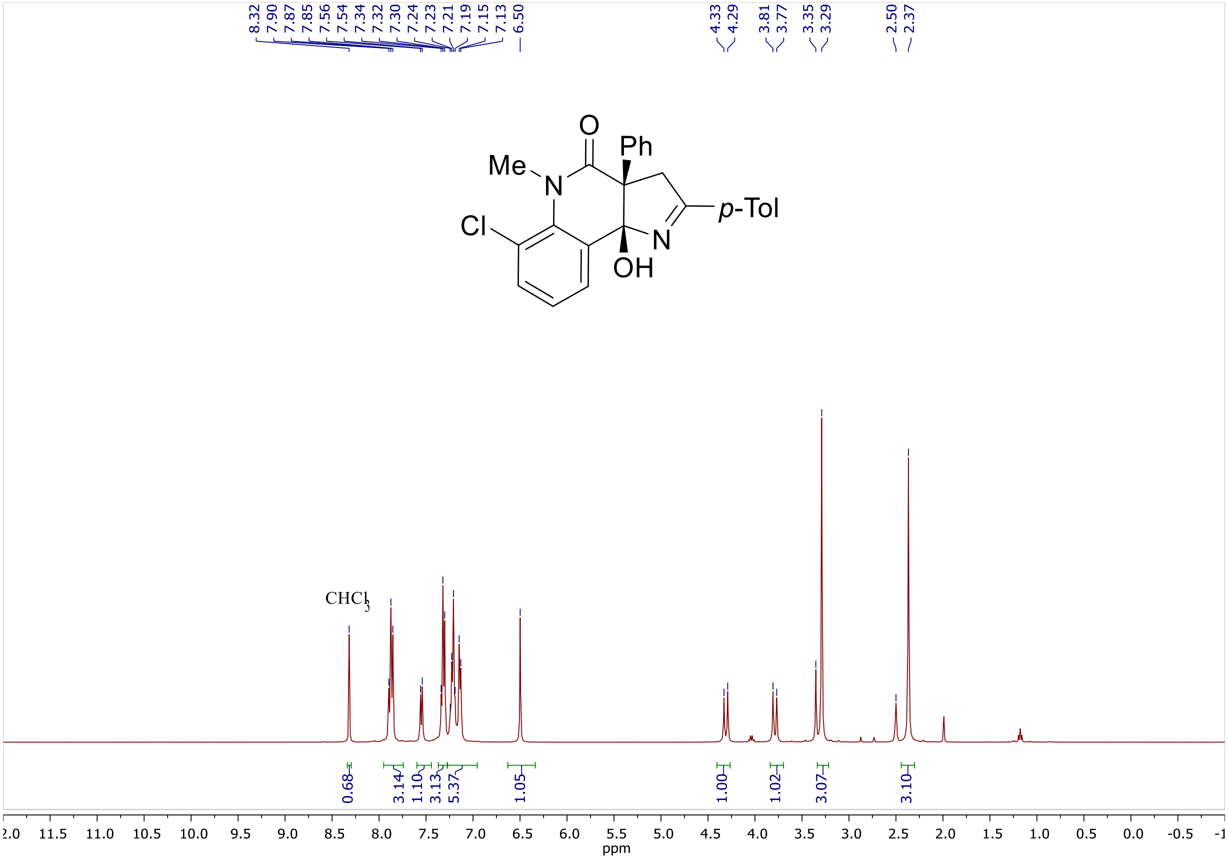


$^{13}C$ NMR (100 MHz, DMSO-d$_6$) spectrum of compound **3k**

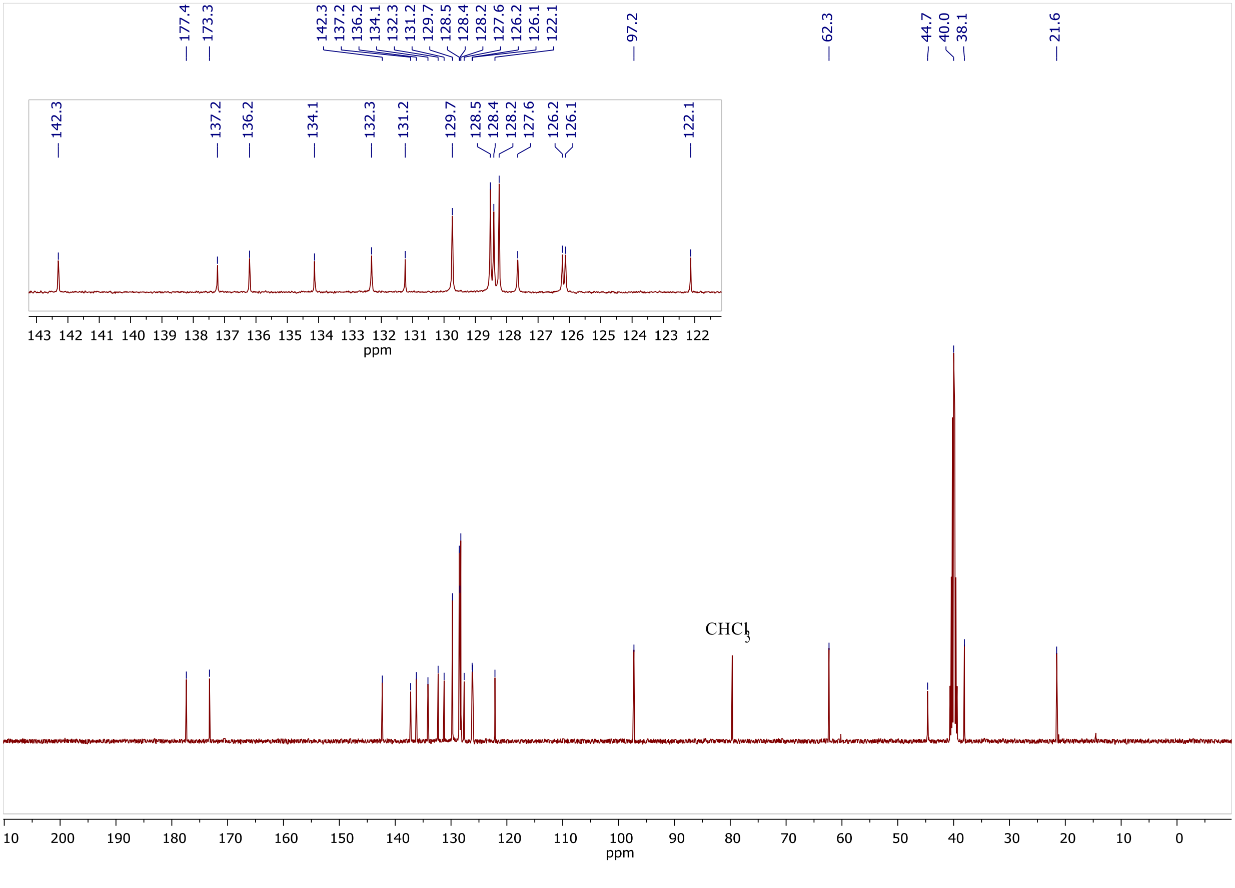

$^{1}$H NMR (400 MHz, DMSO-d$_6$) spectrum of compound **3l**

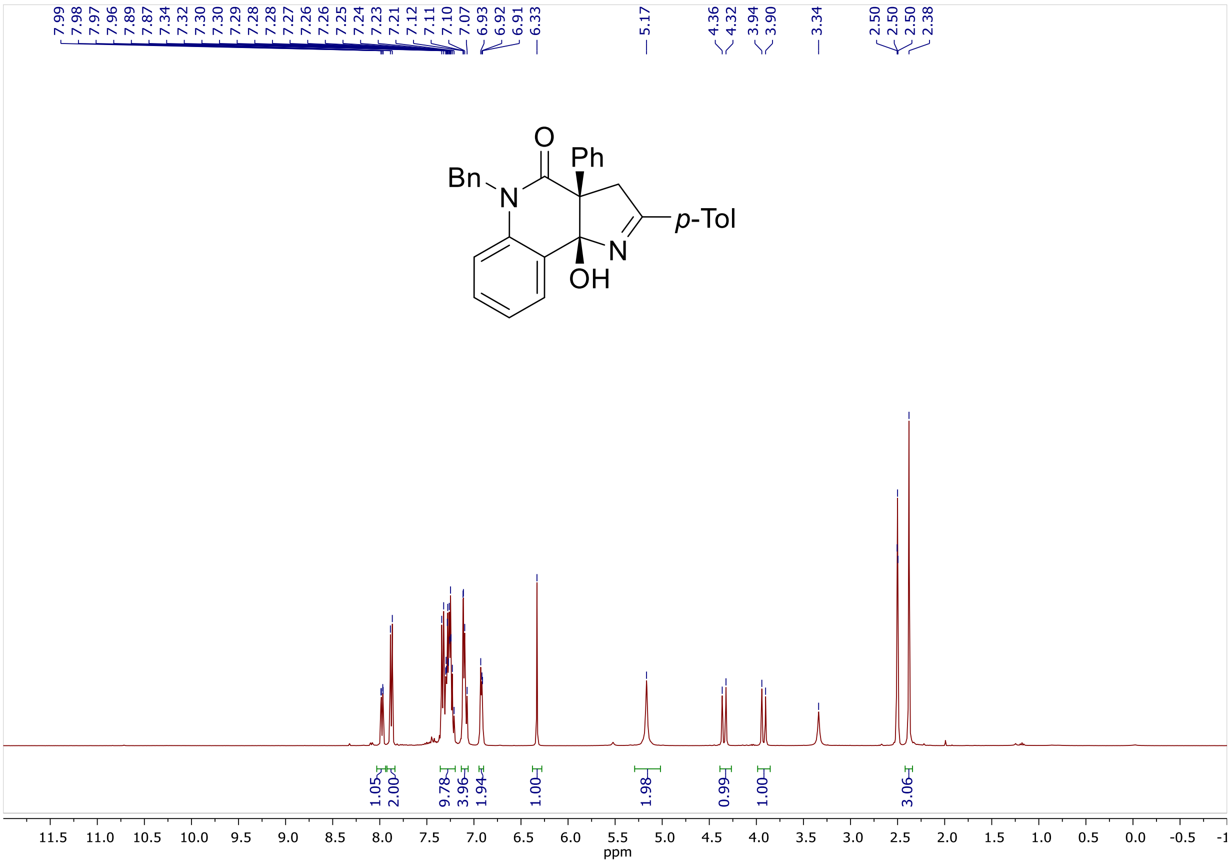


$^{13}$C NMR (100 MHz, DMSO-d$_6$) spectrum of compound **3l**

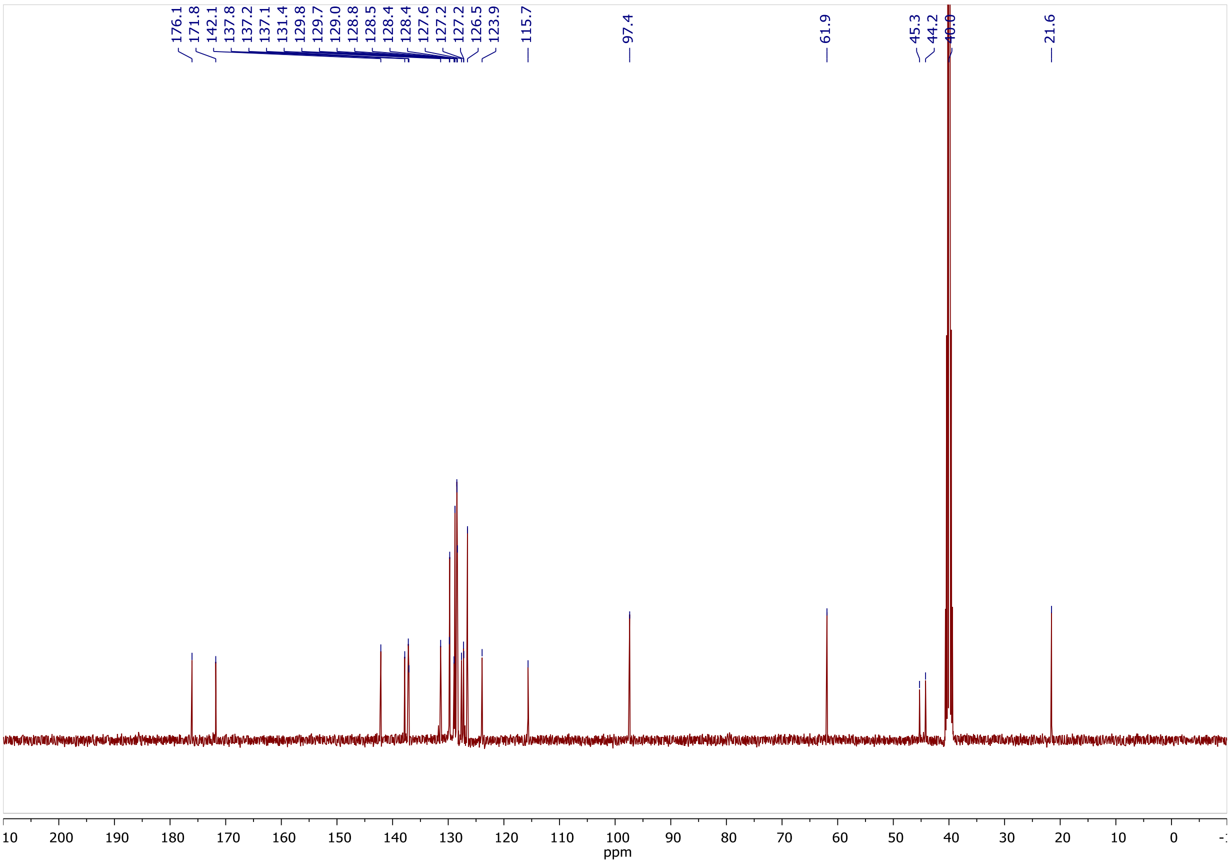

$^{1}H$ NMR (400 MHz, DMSO-d$_6$) spectrum of compound **3m**

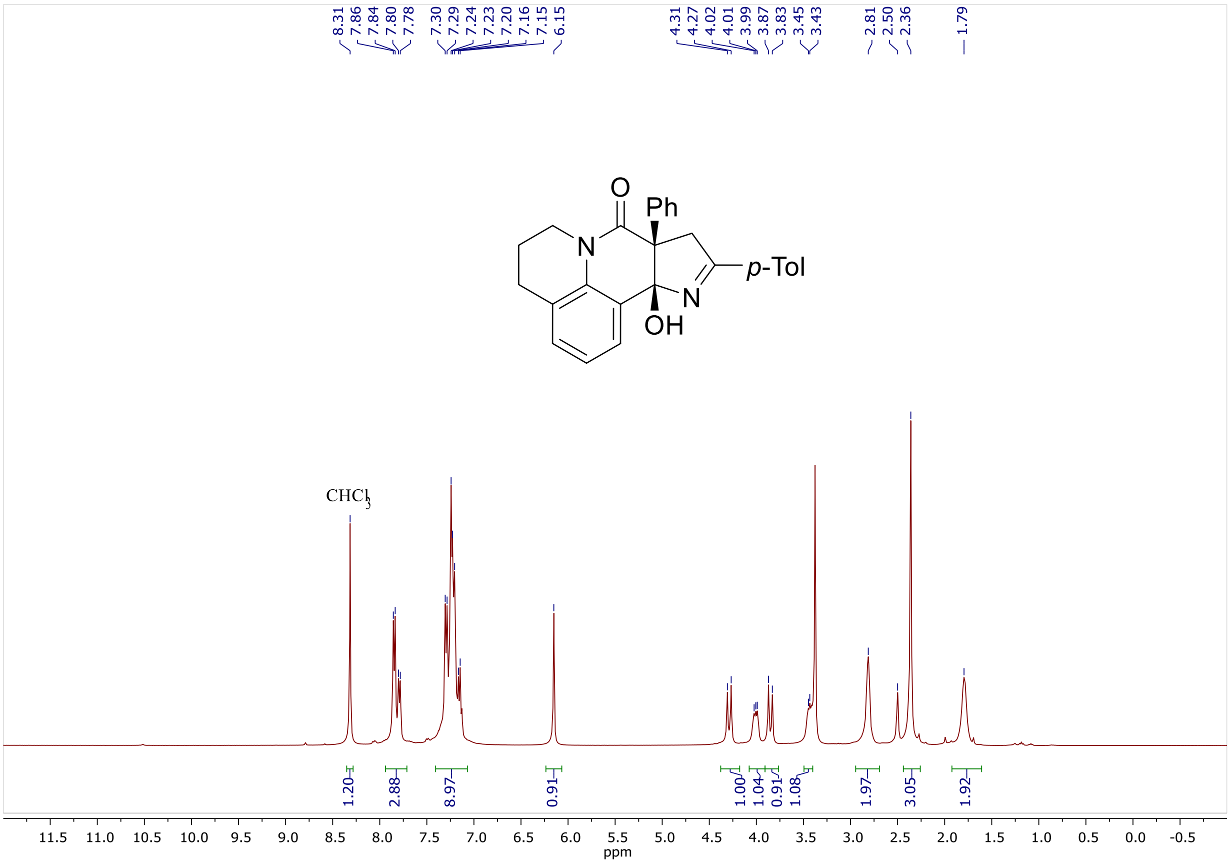


$^{13}C$ NMR (100 MHz, DMSO-d$_6$) spectrum of compound **3m**

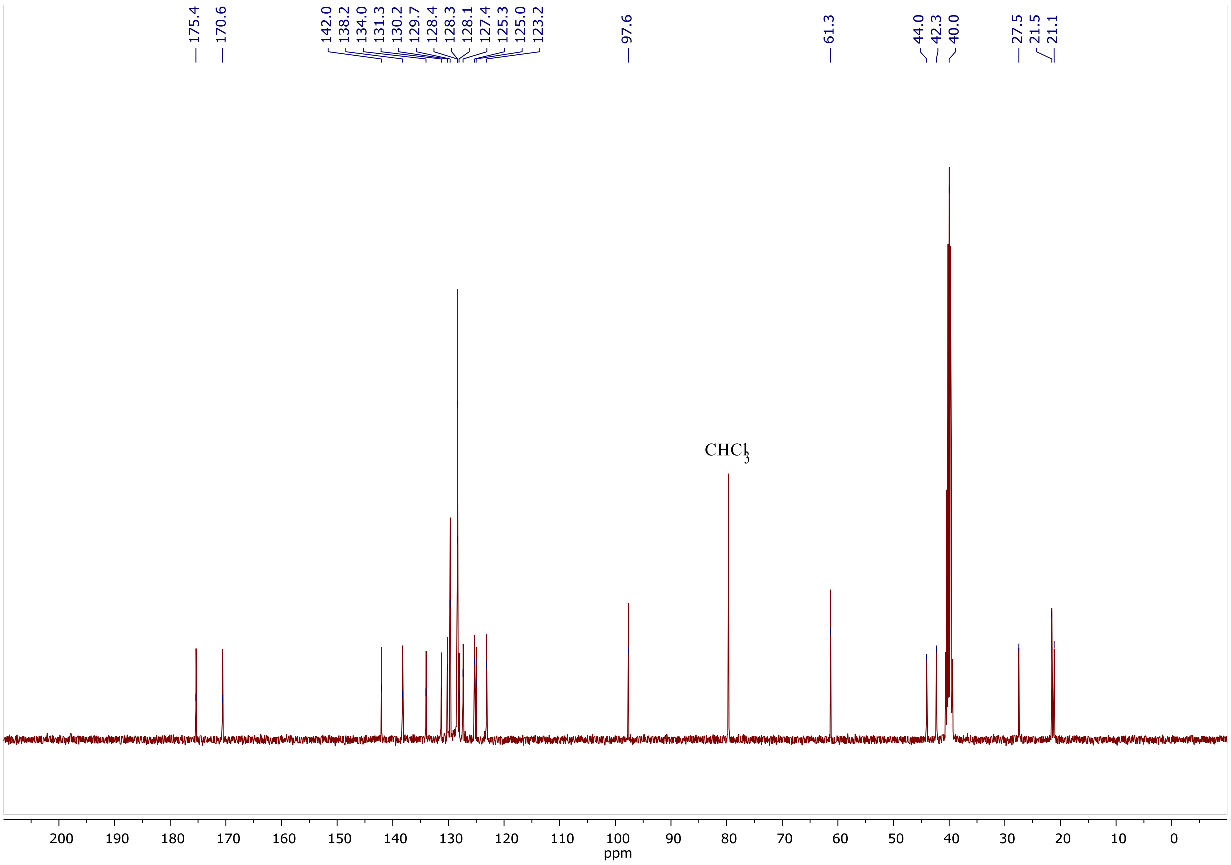

$^{1}H$ NMR (400 MHz, DMSO-$d_6$) spectrum of compound **3n**

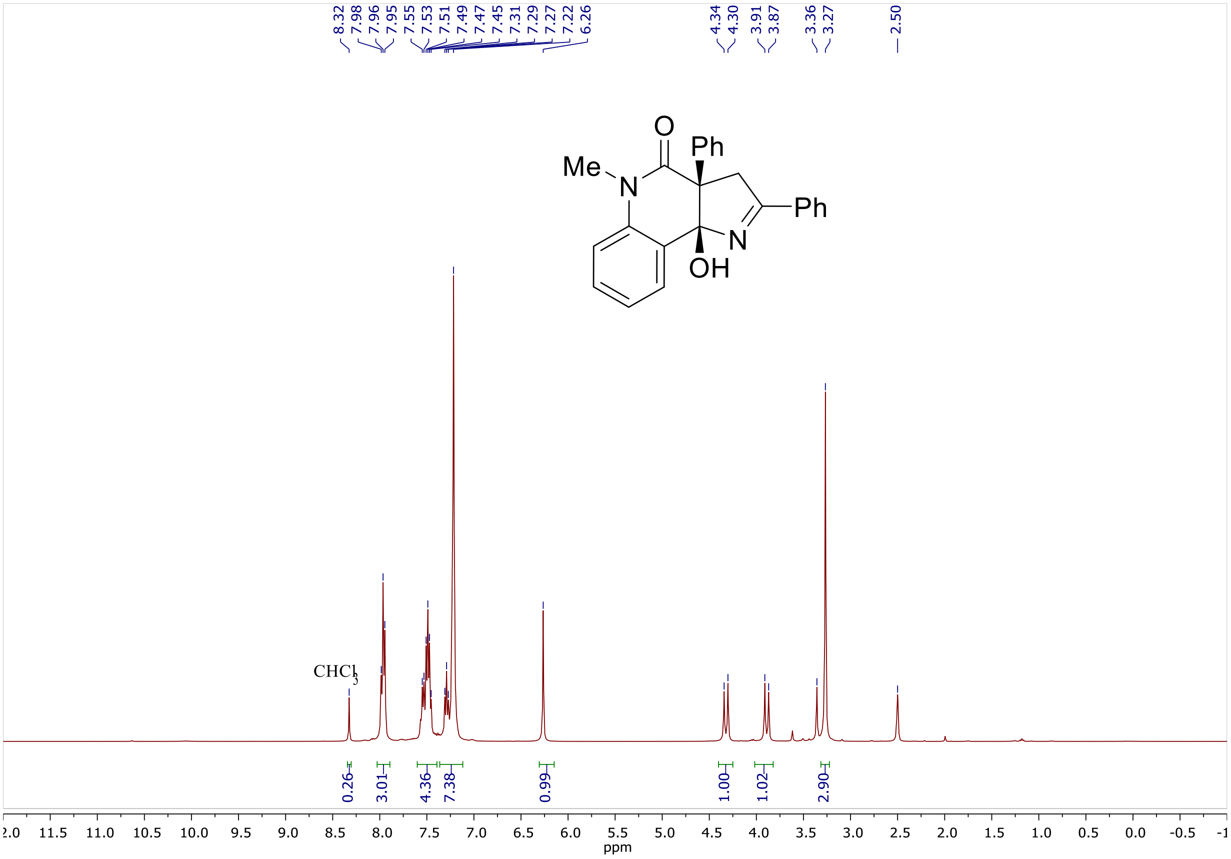


$^{13}C$ NMR (100 MHz, DMSO-$d_6$) spectrum of compound **3n**

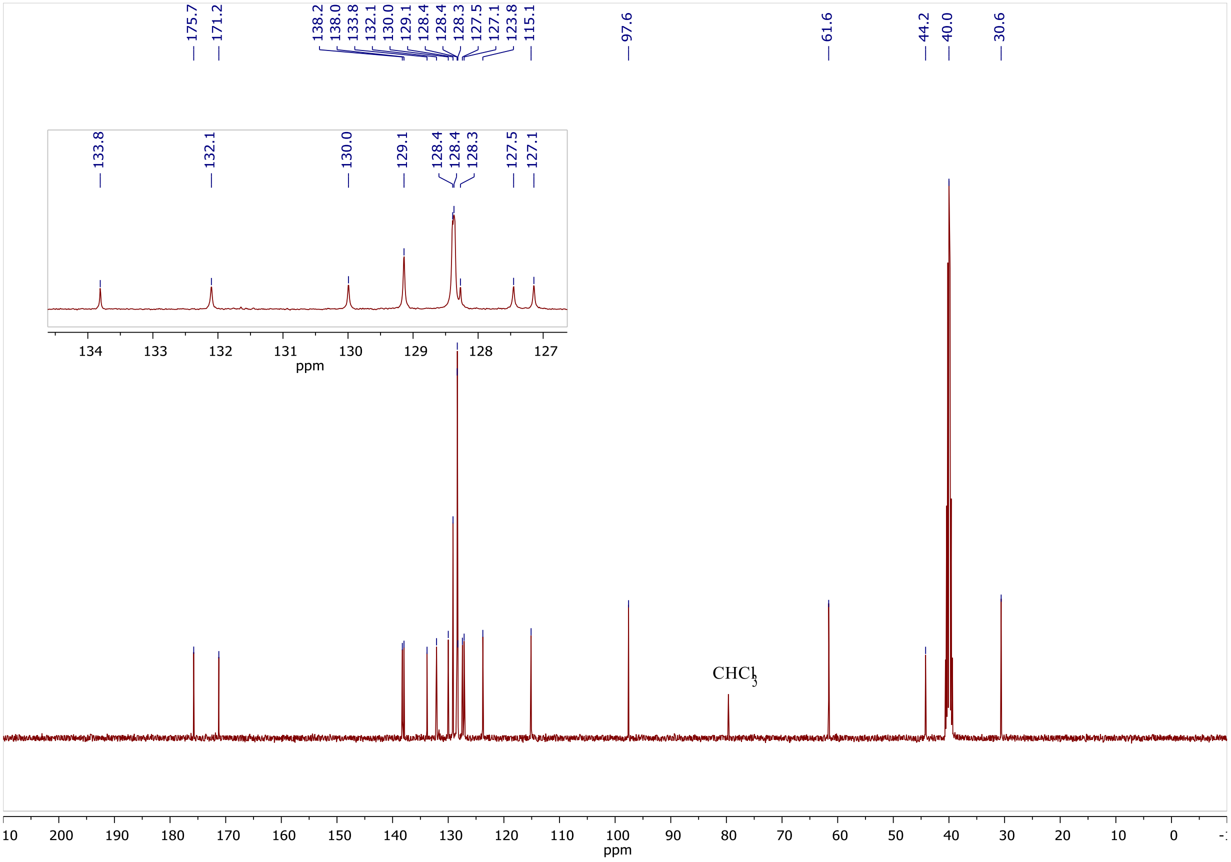

$^{1}$H NMR (400 MHz, DMSO-d$_6$) spectrum of compound **3o**

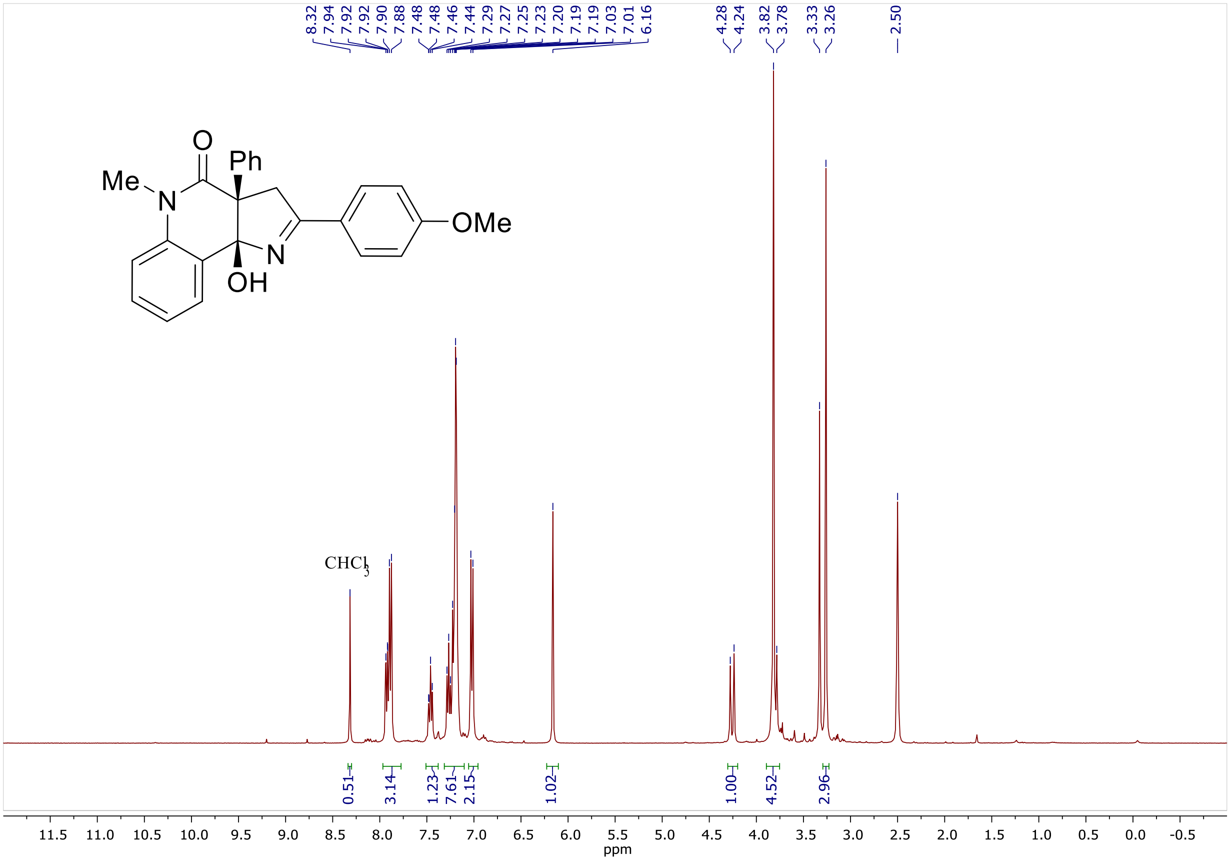


$^{13}$C NMR (100 MHz, DMSO-d$_6$) spectrum of compound **3o**

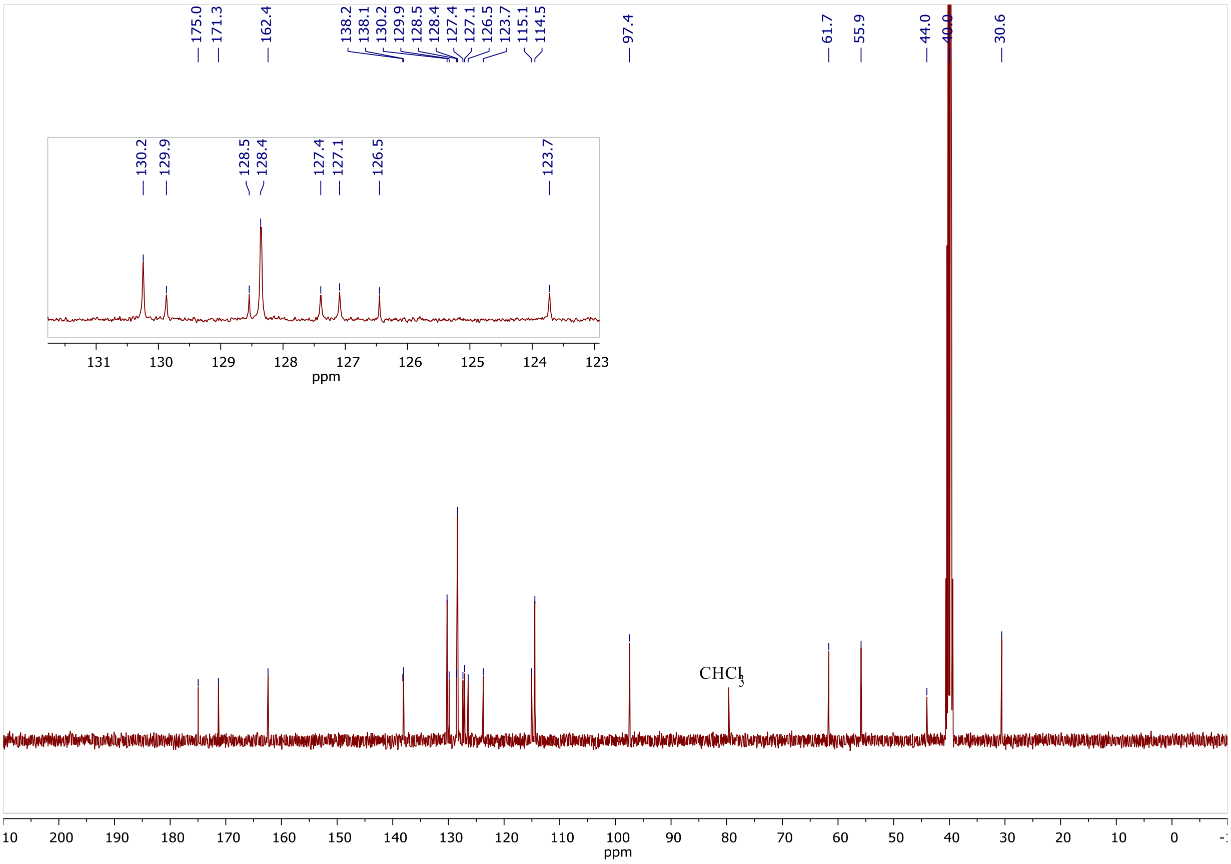

$^{1}H$ NMR (400 MHz, DMSO-$d_6$) spectrum of compound **3p**

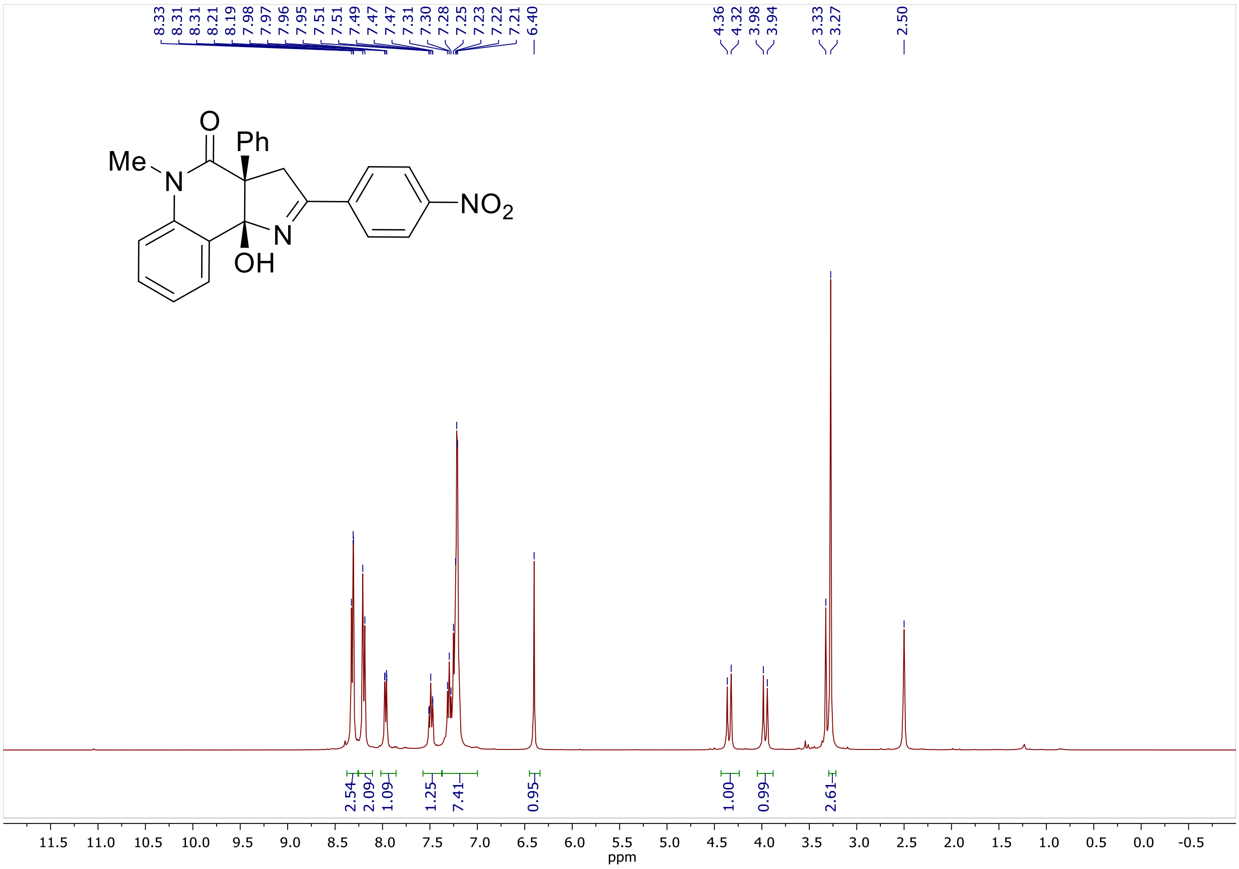


$^{13}C$ NMR (100 MHz, DMSO-$d_6$) spectrum of compound **3p**

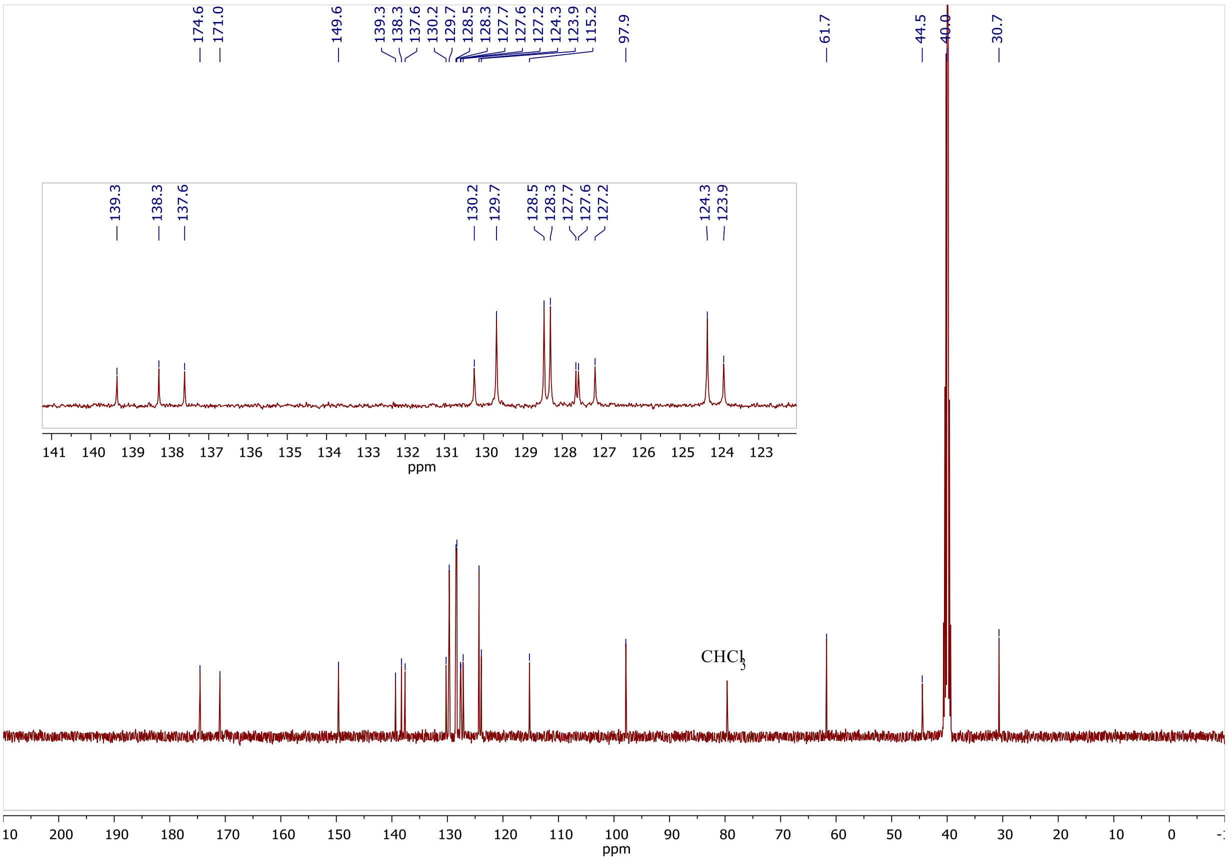

$^{1}$H NMR (400 MHz, DMSO-d$_6$) spectrum of compound **6a**

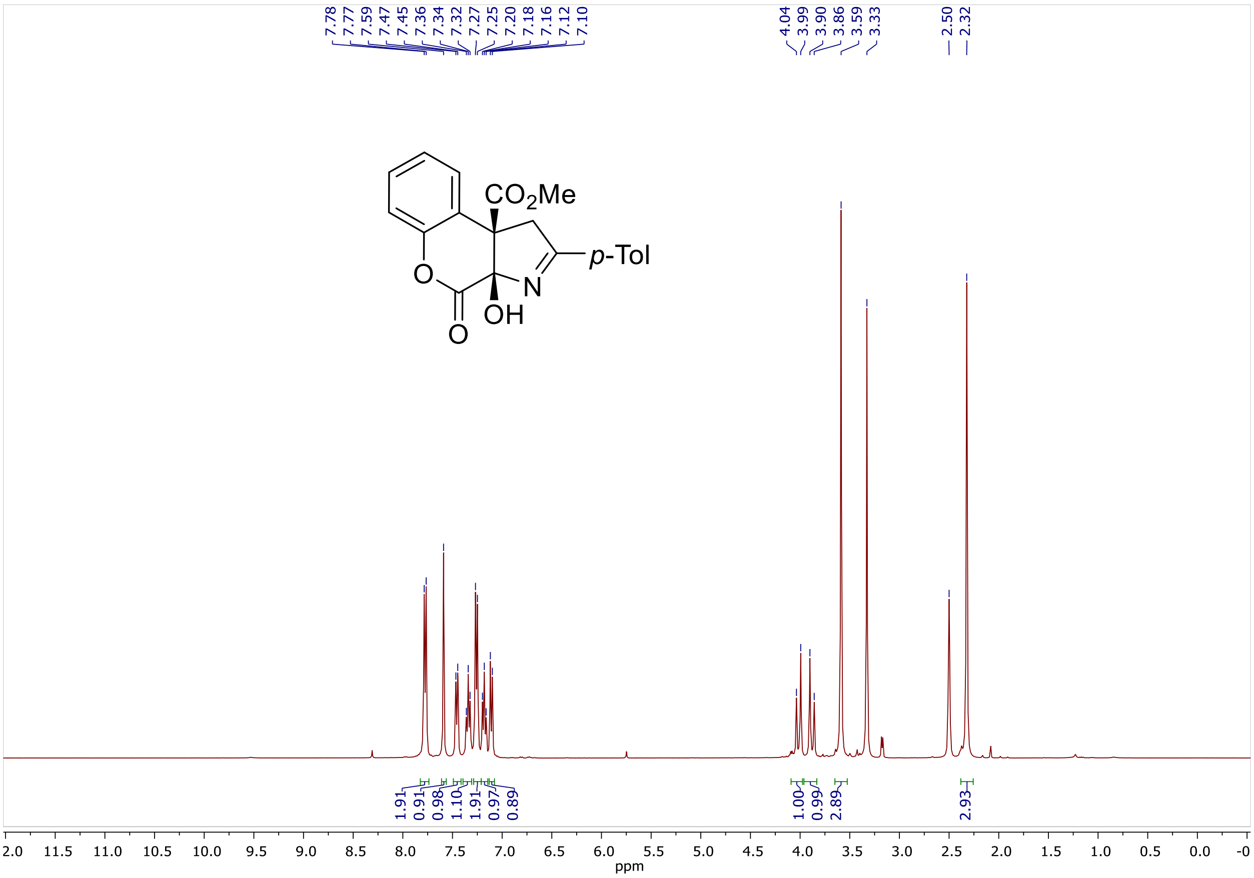


$^{13}$C NMR (100 MHz, DMSO-d$_6$) spectrum of compound **6a**

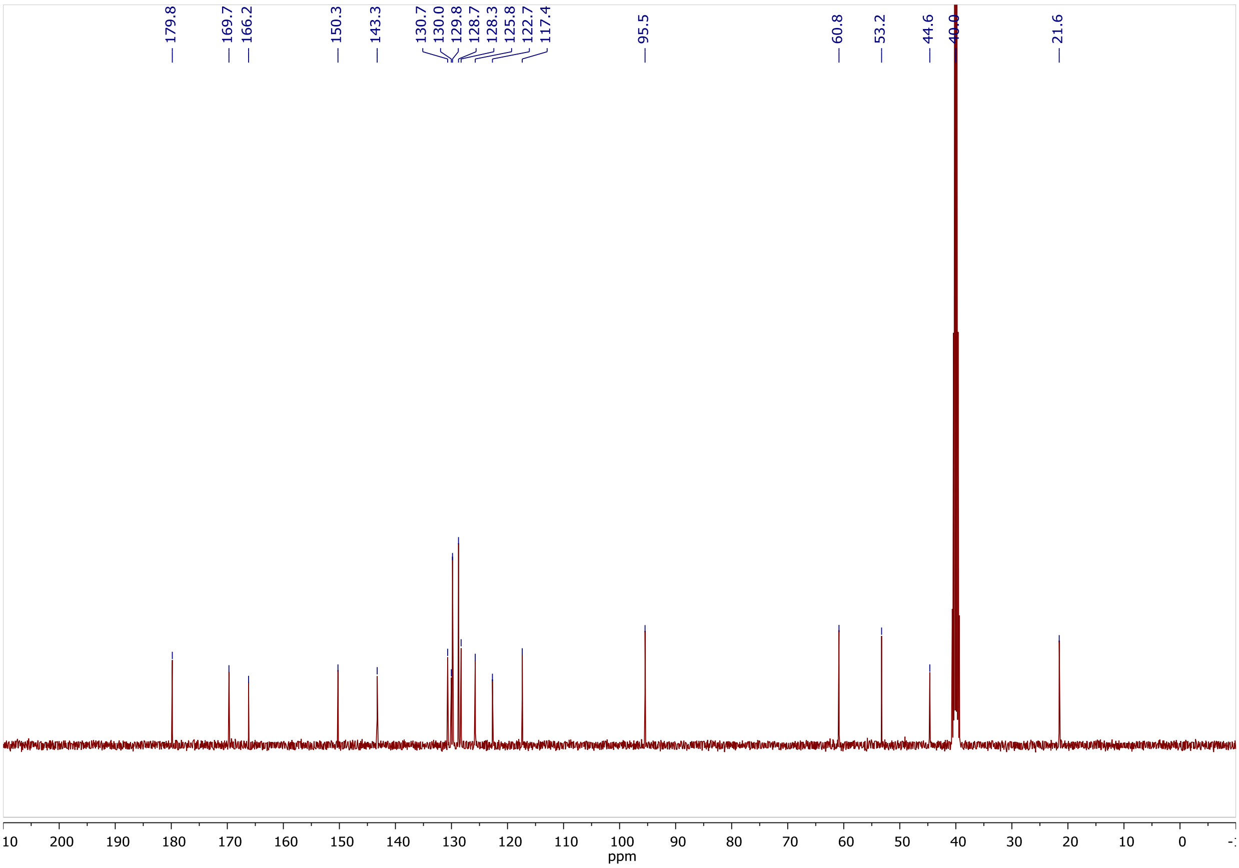

$^{1}$H NMR (400 MHz, DMSO-d$_6$) spectrum of compound **6b**

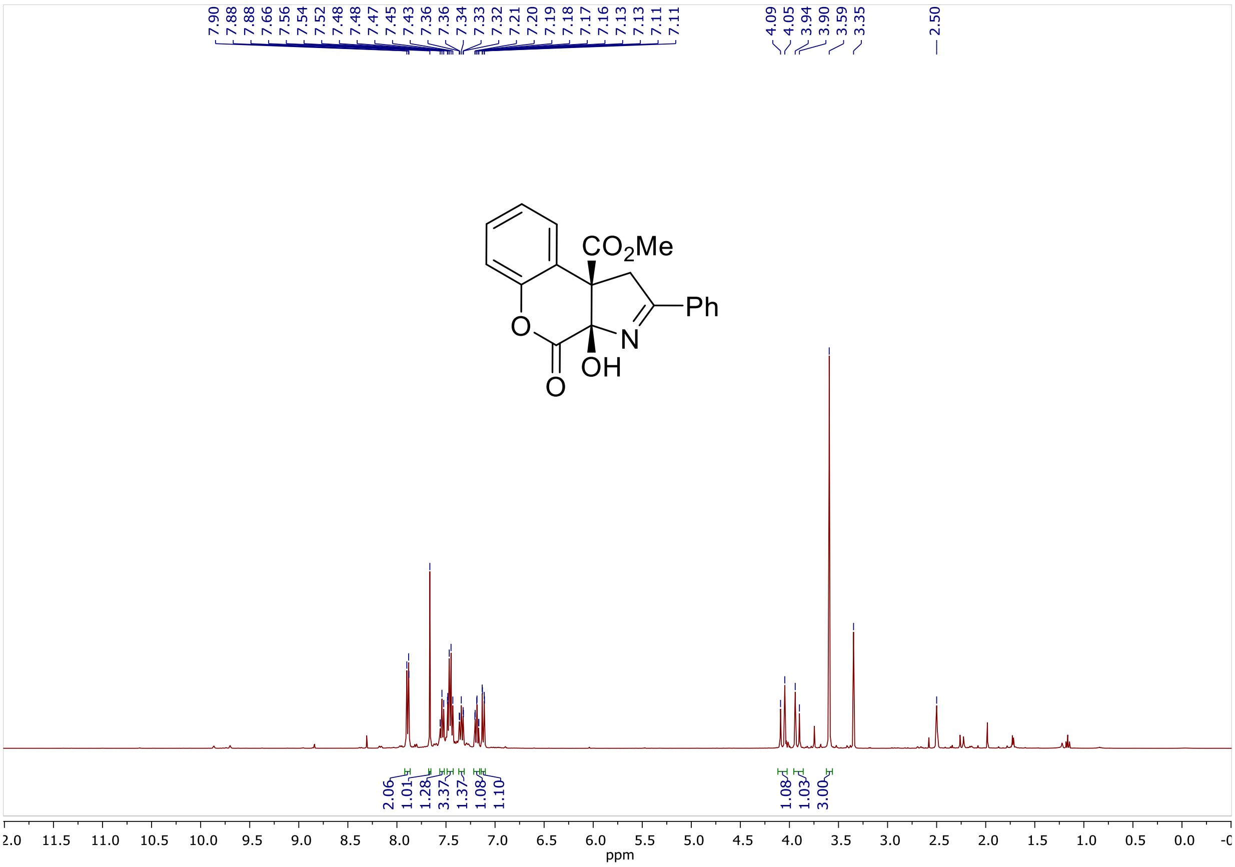


$^{13}$C NMR (100 MHz, DMSO-d$_6$) spectrum of compound **6b**

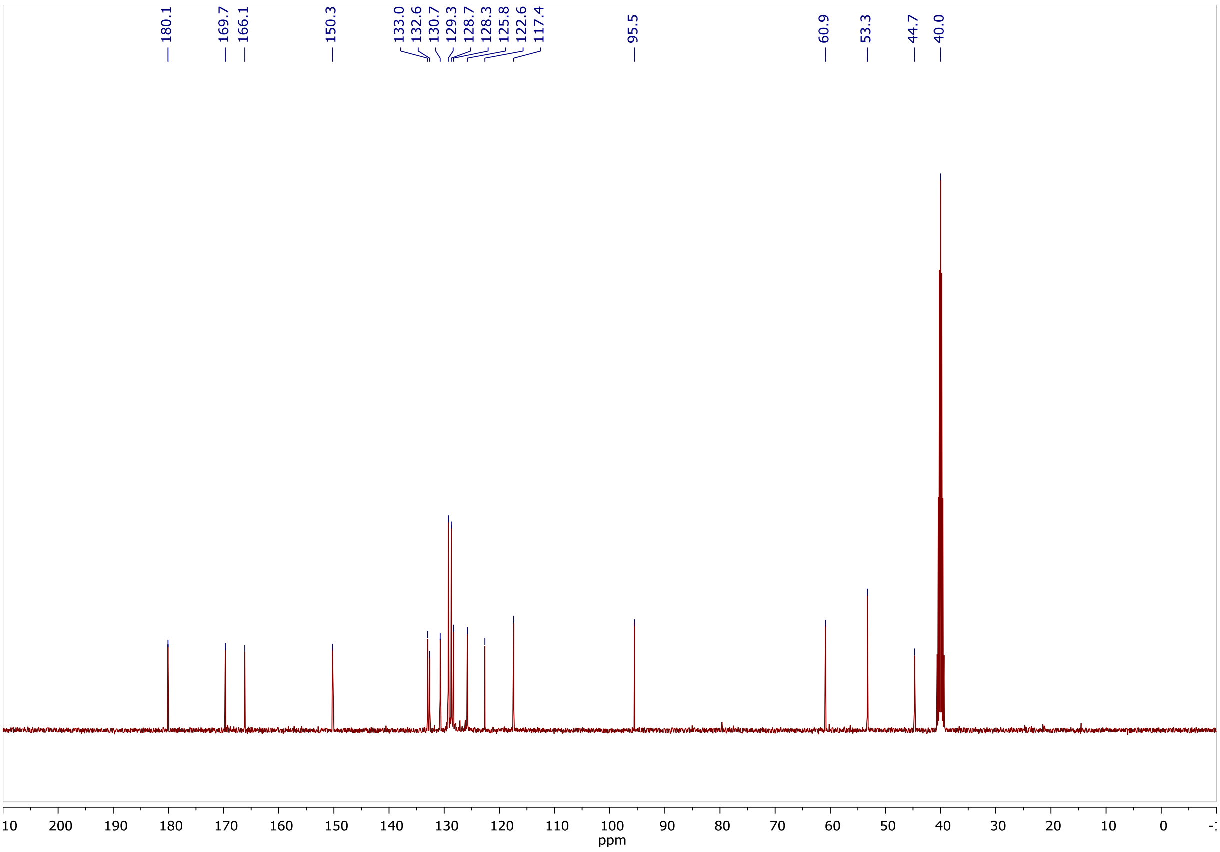

$^{1}H$ NMR (400 MHz, DMSO-$d_6$) spectrum of compound **6c**

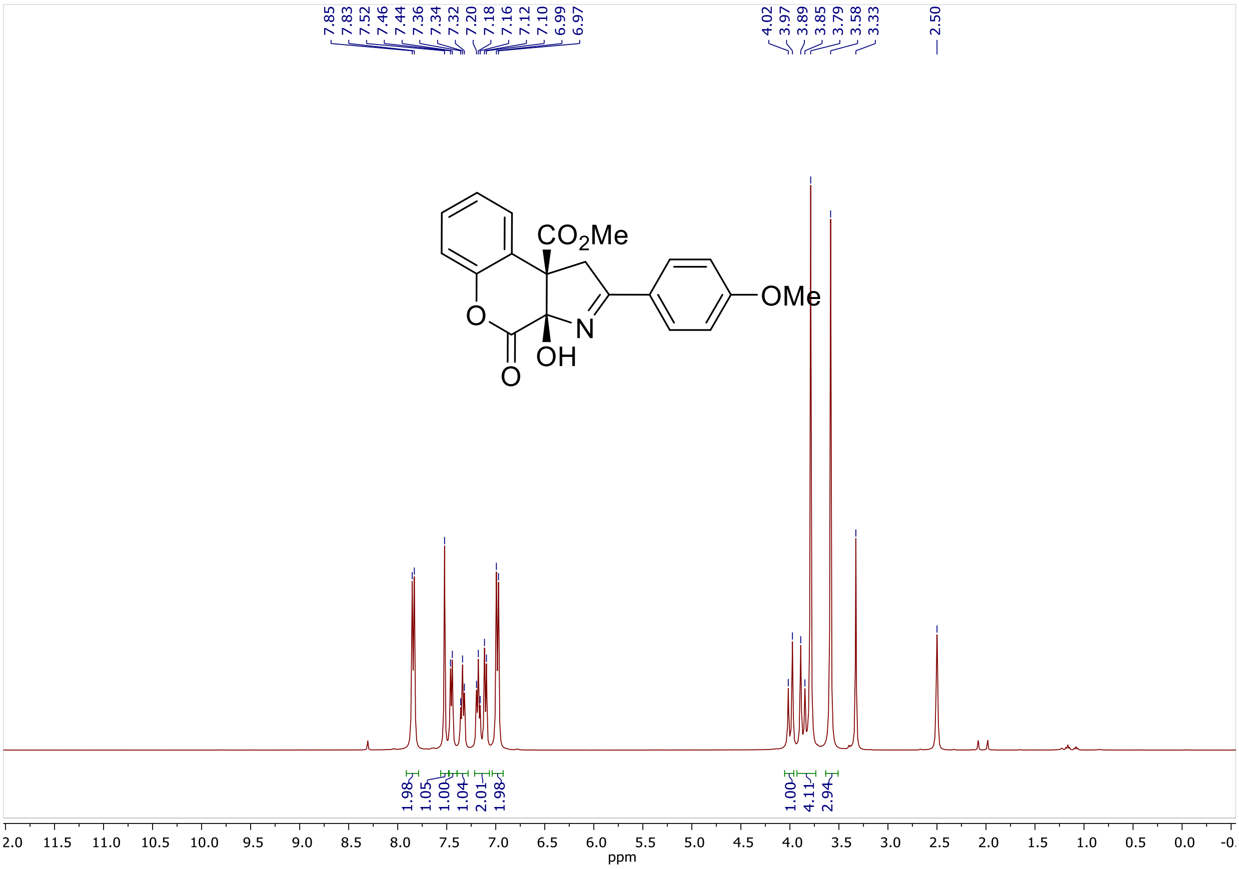


$^{13}C$ NMR (100 MHz, DMSO-$d_6$) spectrum of compound **6c**

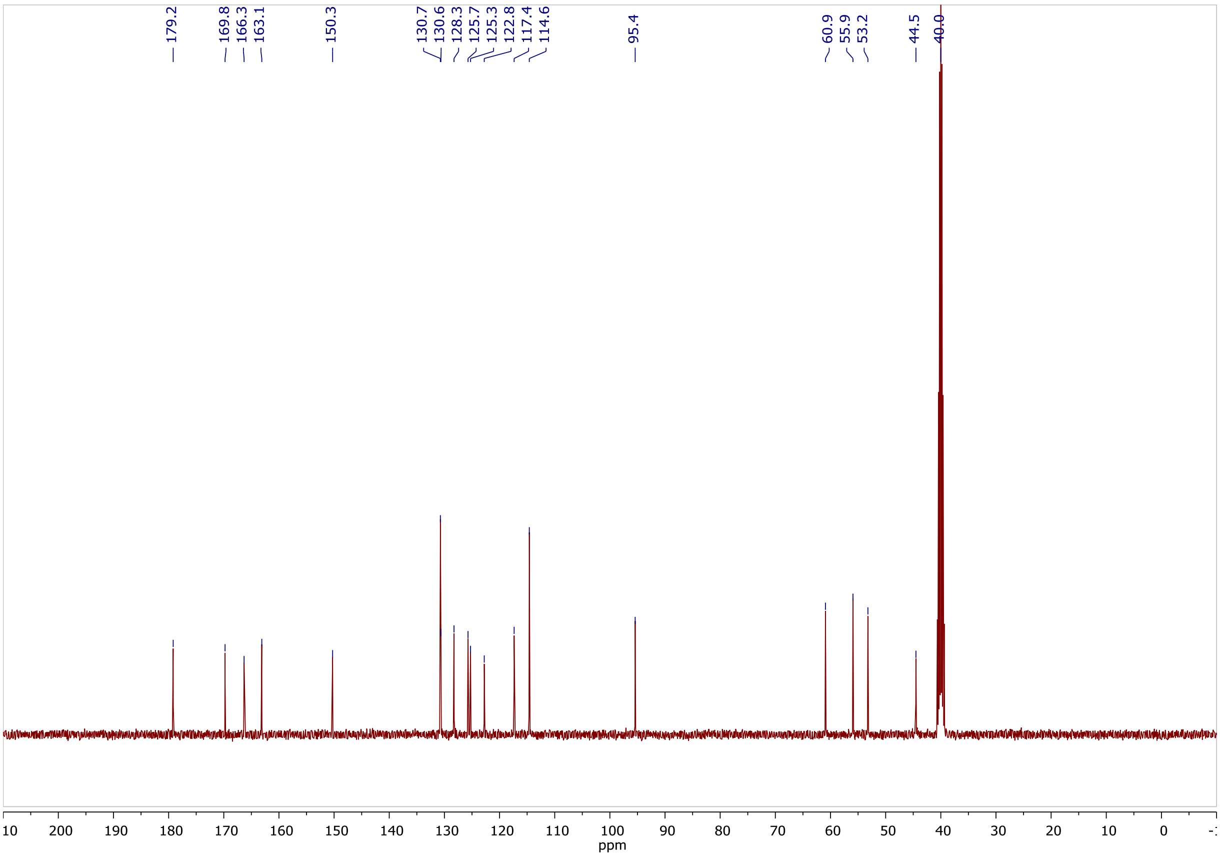

$^{1}H$ NMR (400 MHz, DMSO-$d_6$) spectrum of compound **6d**

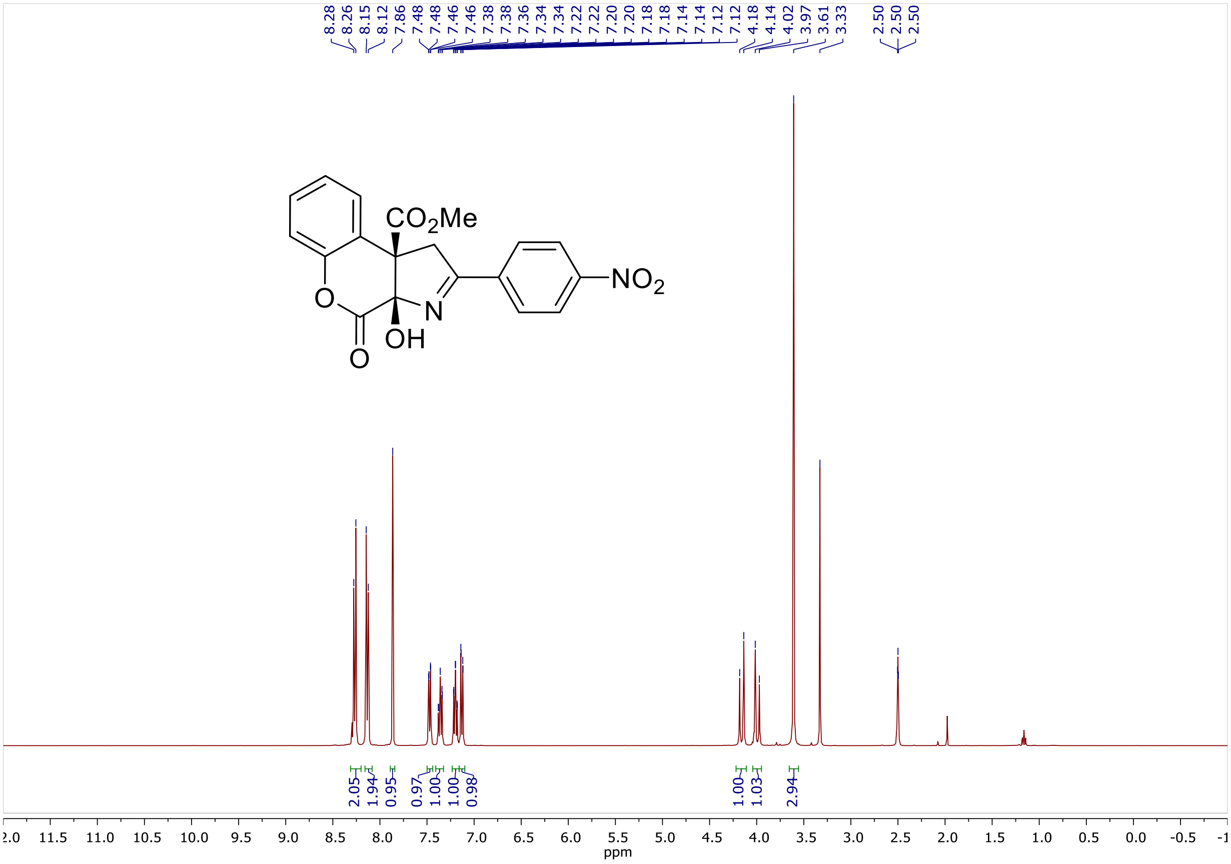


$^{13}C$ NMR (100 MHz, DMSO-$d_6$) spectrum of compound **6d**

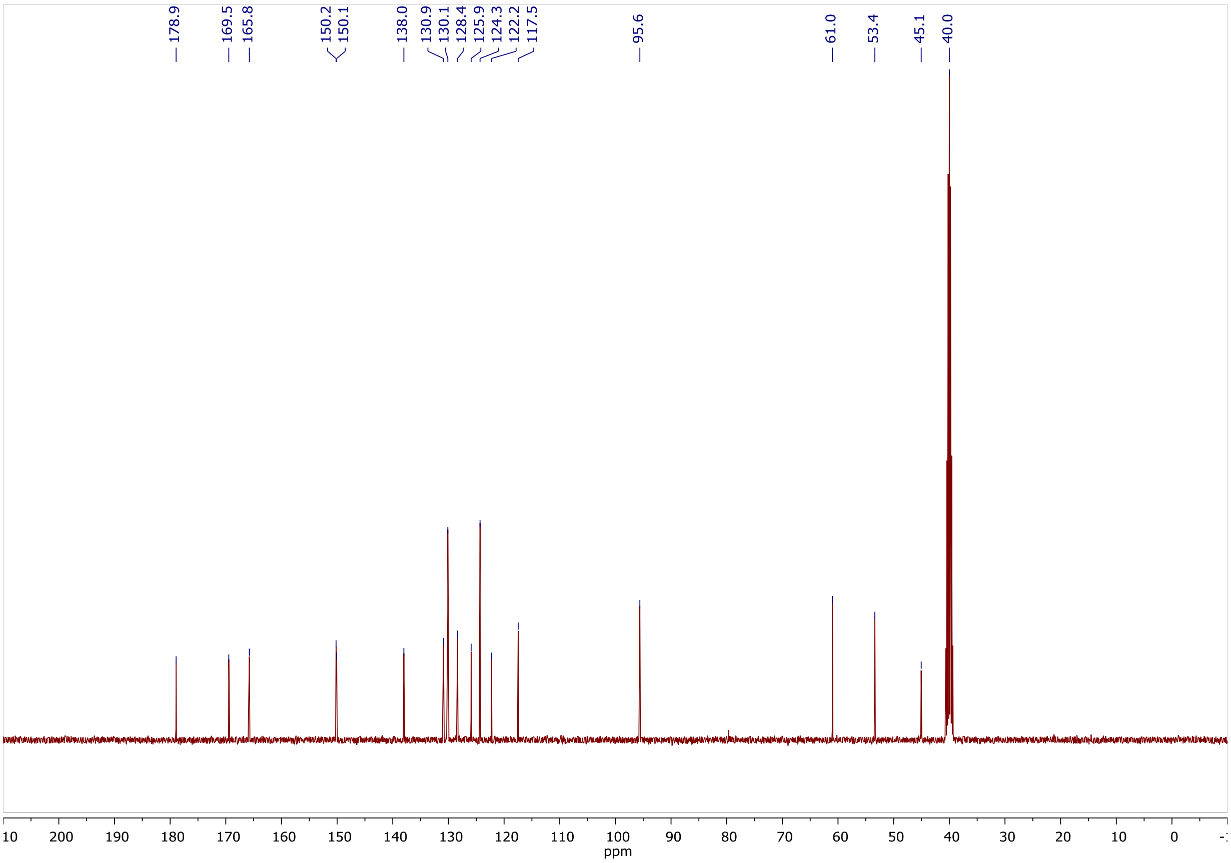

$^{1}H$ NMR (400 MHz, $CDCl_3$/DMSO-$d_6$ mixture) spectrum of compound **8**

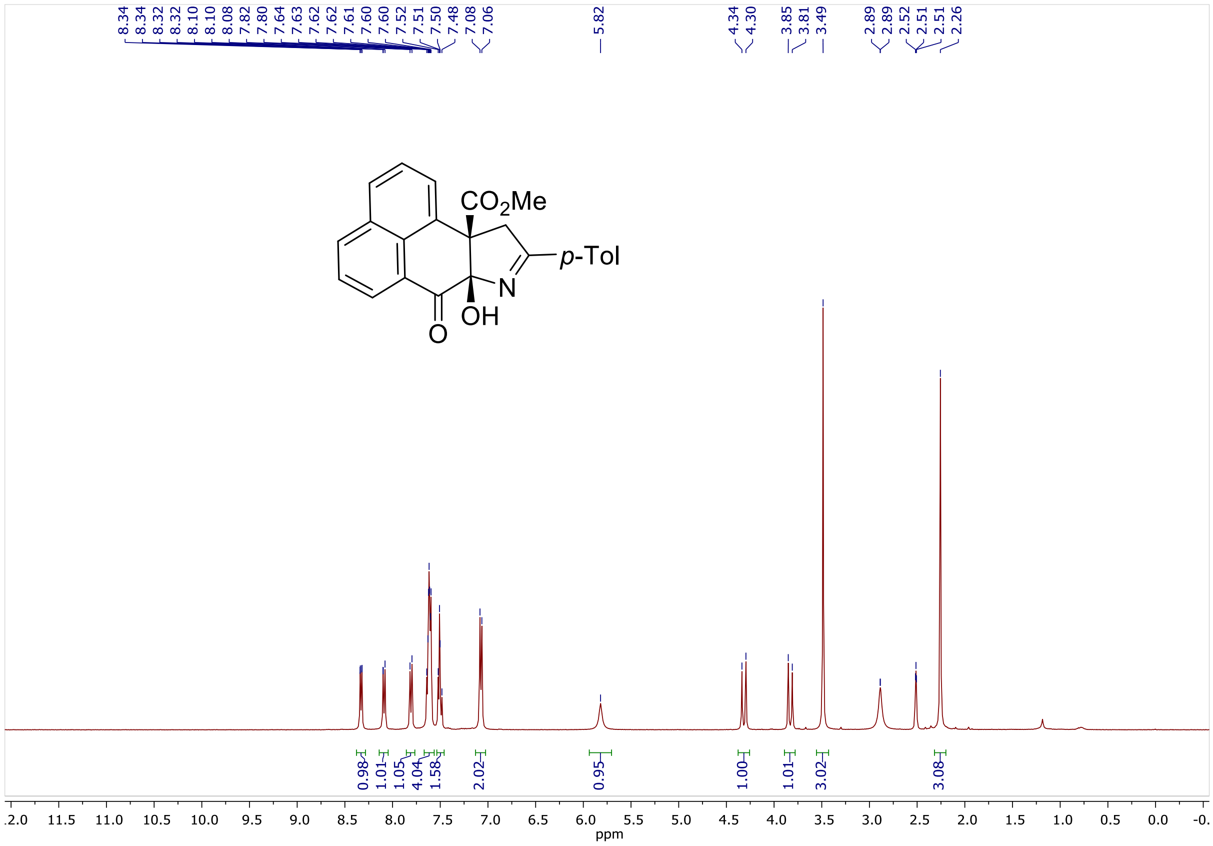


$^{13}C$ NMR (100 MHz, $CDCl_3$/DMSO-$d_6$ mixture) spectrum of compound **8**

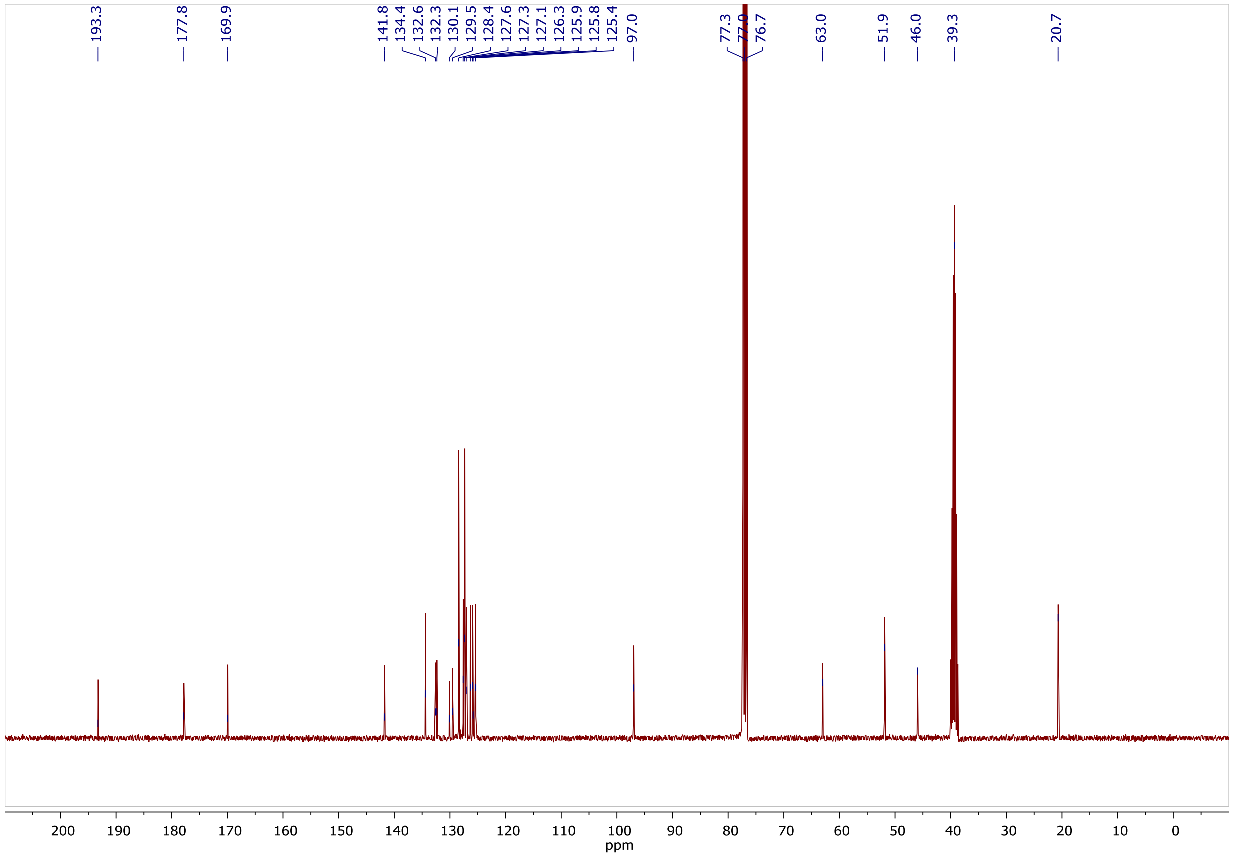

$^{1}$H NMR (400 MHz, $CDCl_3$) spectrum of compound **15**

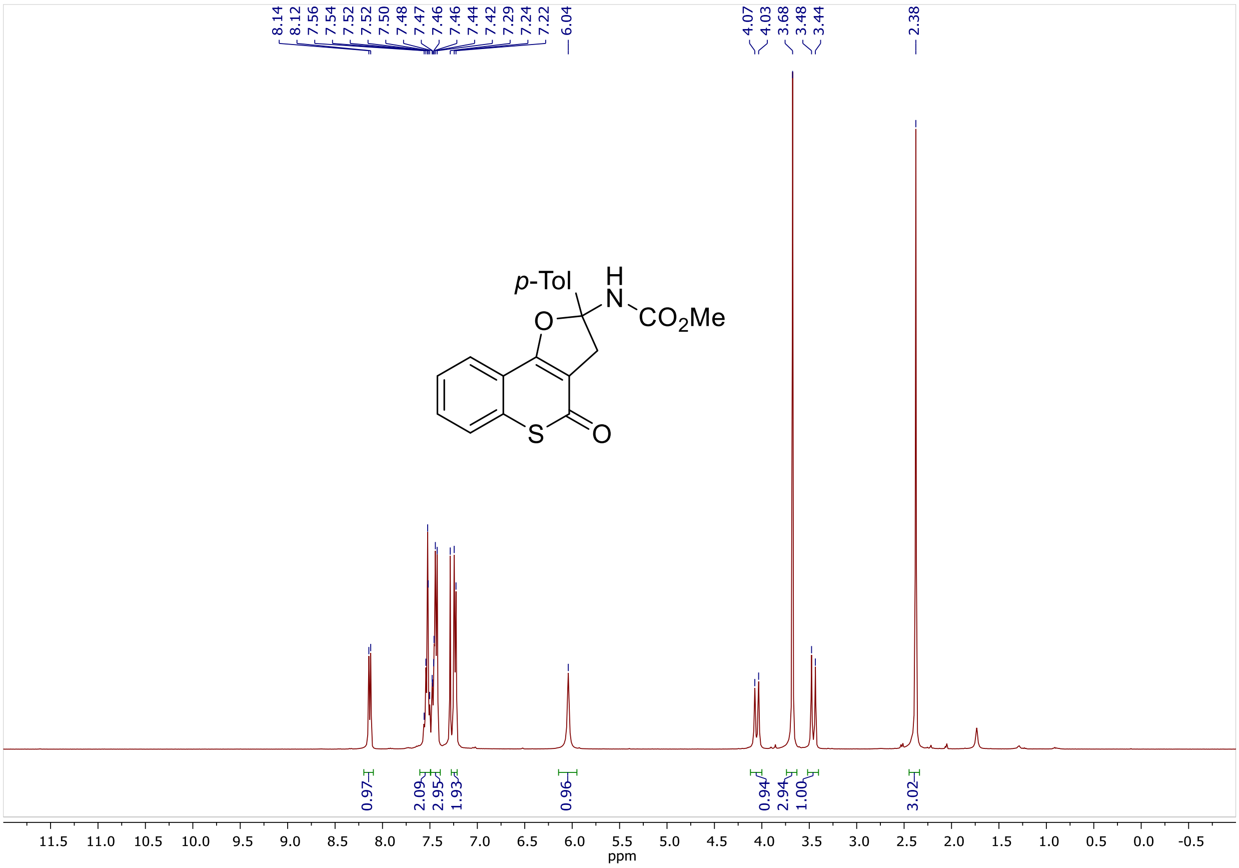


$^{13}$C NMR (100 MHz, $CDCl_3$) spectrum of compound **15**

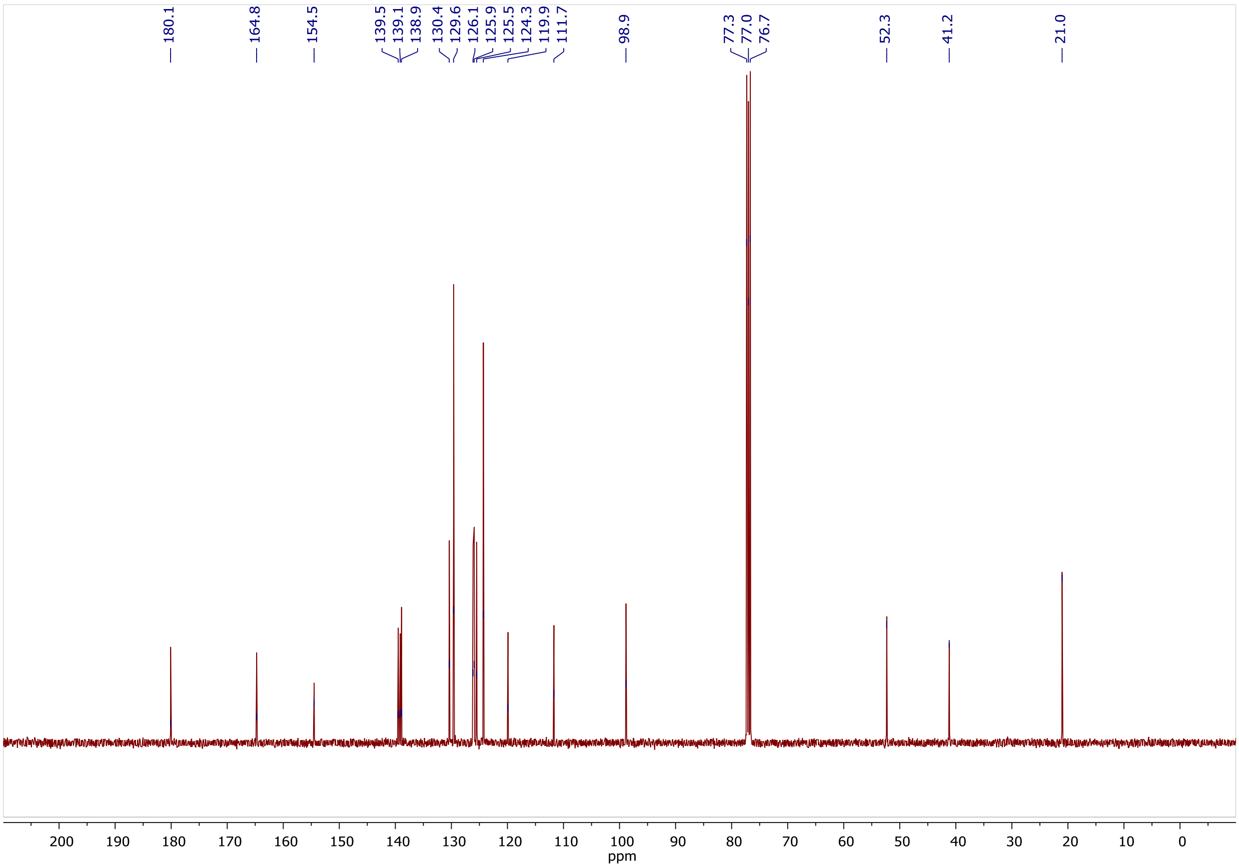

$^{1}$H NMR (400 MHz, $CDCl_3$) spectrum of compound **16**

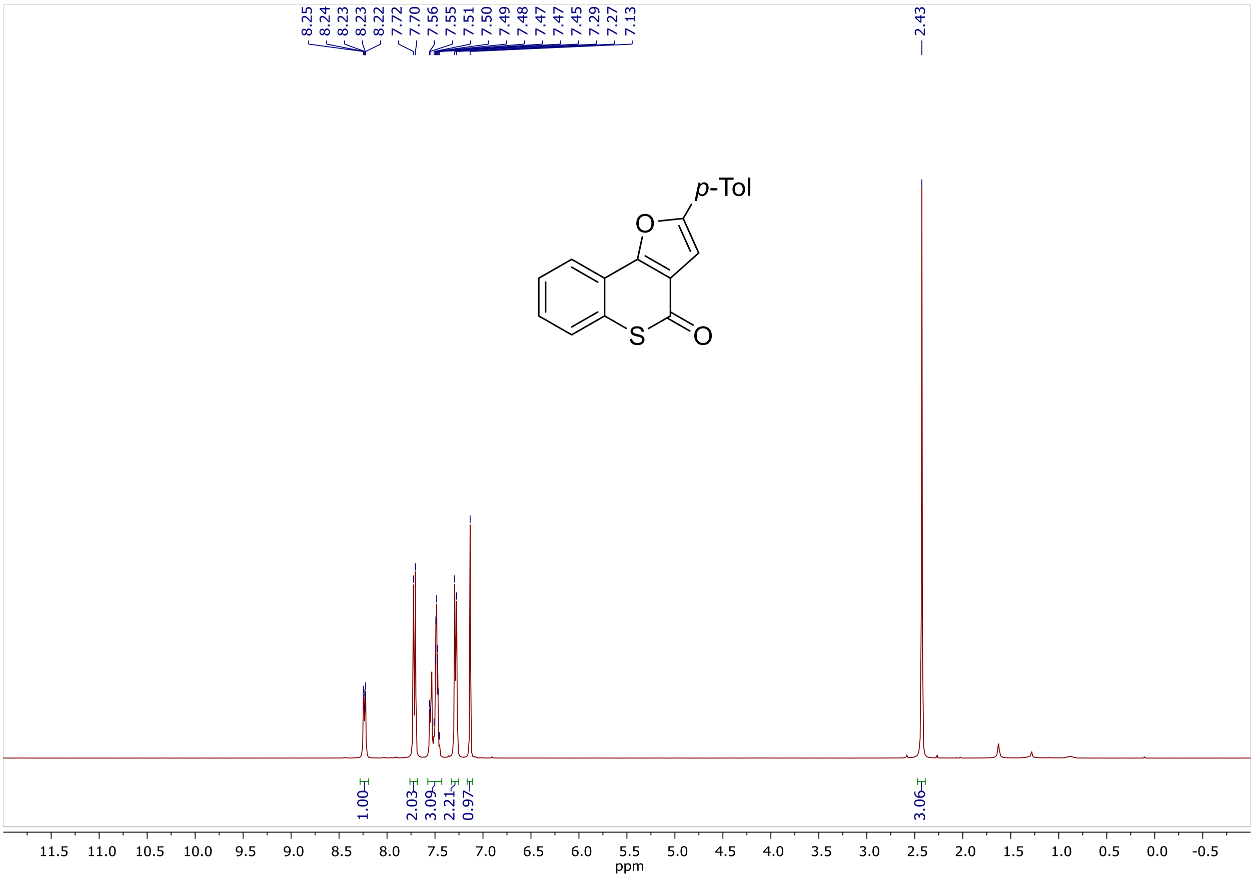


$^{13}$C NMR (100 MHz, $CDCl_3$) spectrum of compound **16**

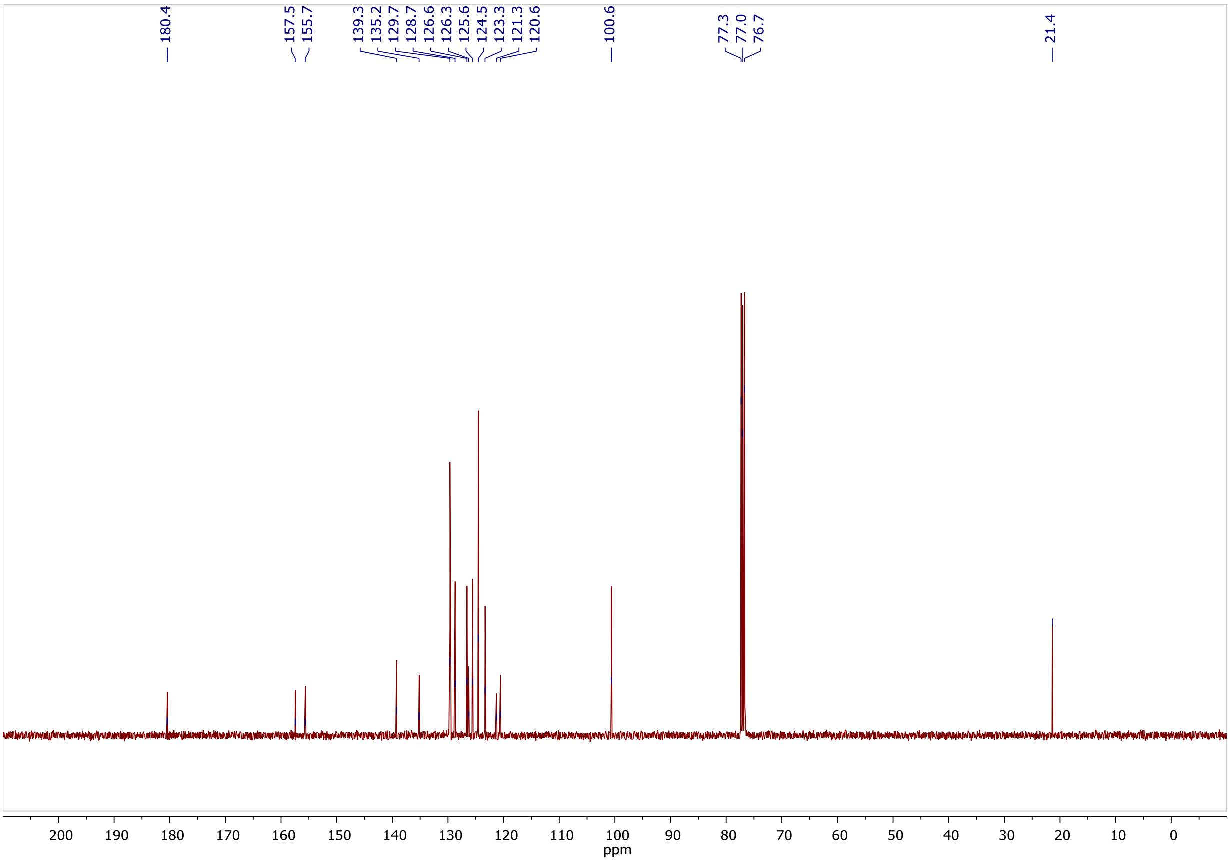

$^{1}H$ NMR (400 MHz, DMSO-$d_6$) spectrum of compound **18**

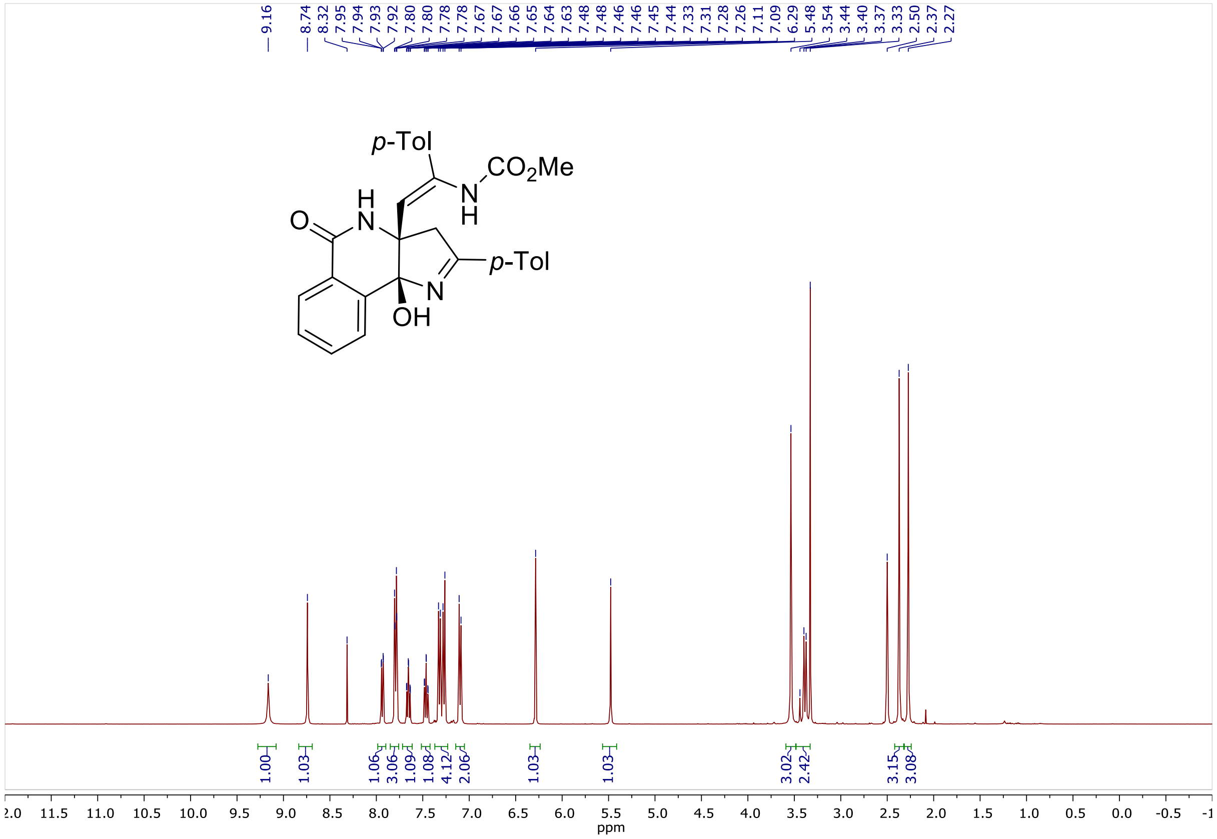

$^{13}C$ NMR (100 MHz, DMSO-$d_6$) spectrum of compound **18**

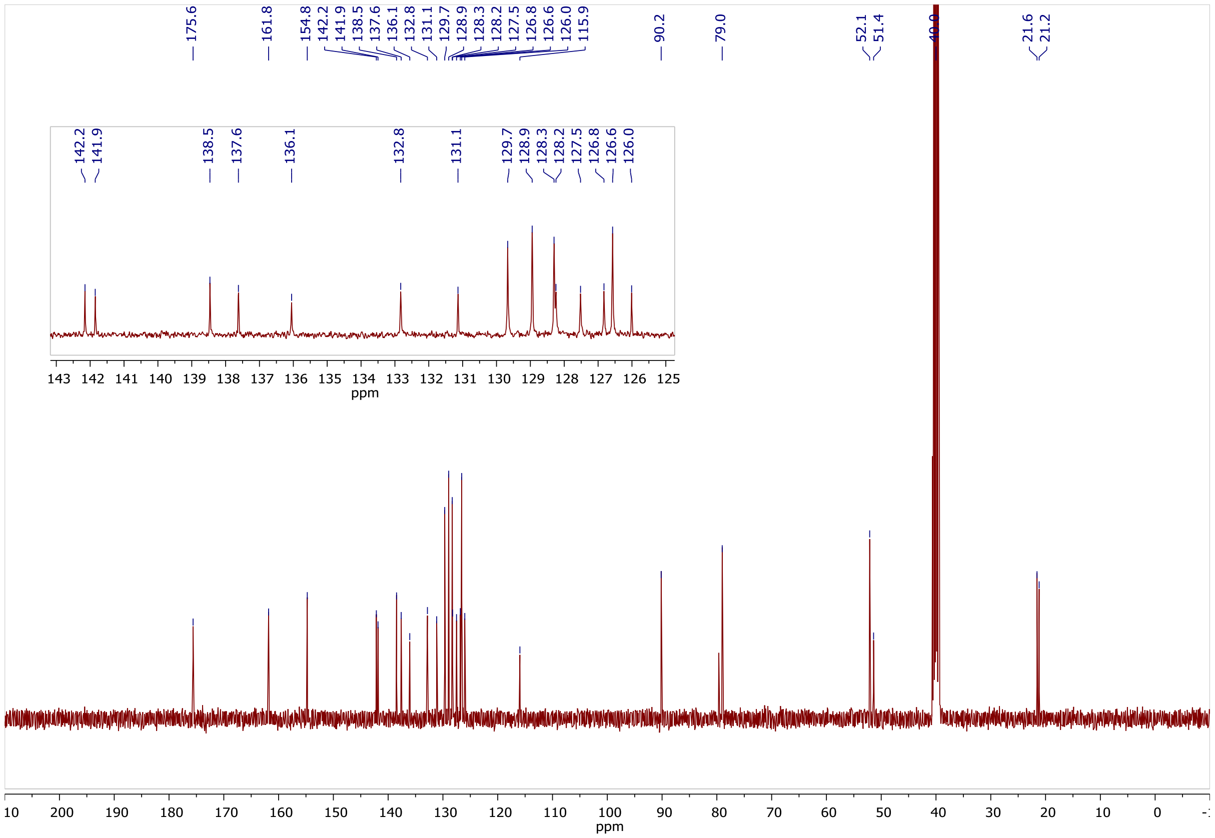

HSQC $^{1}H$-$^{13}C$ NMR (DMSO-$d_6$) spectrum of compound **18**

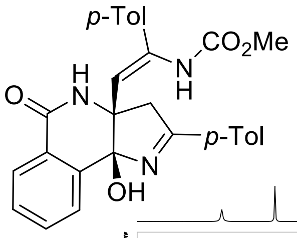


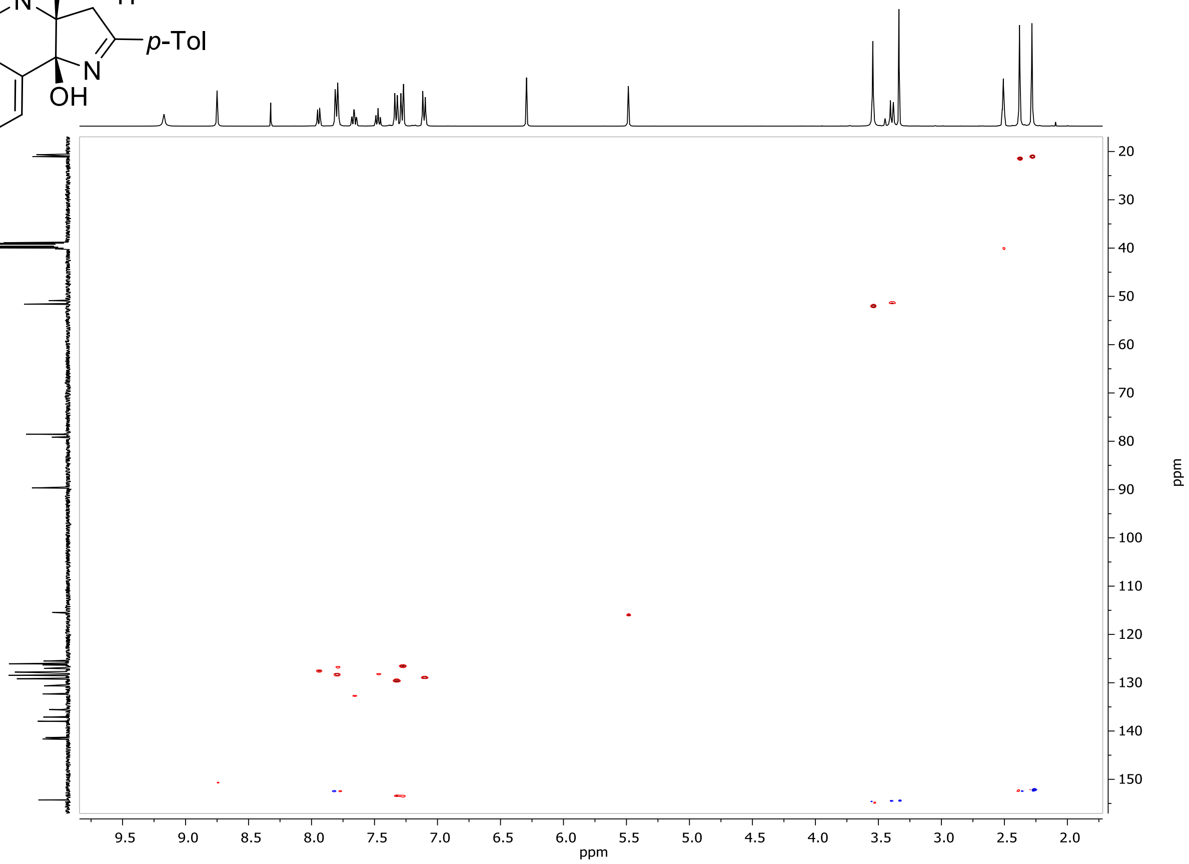


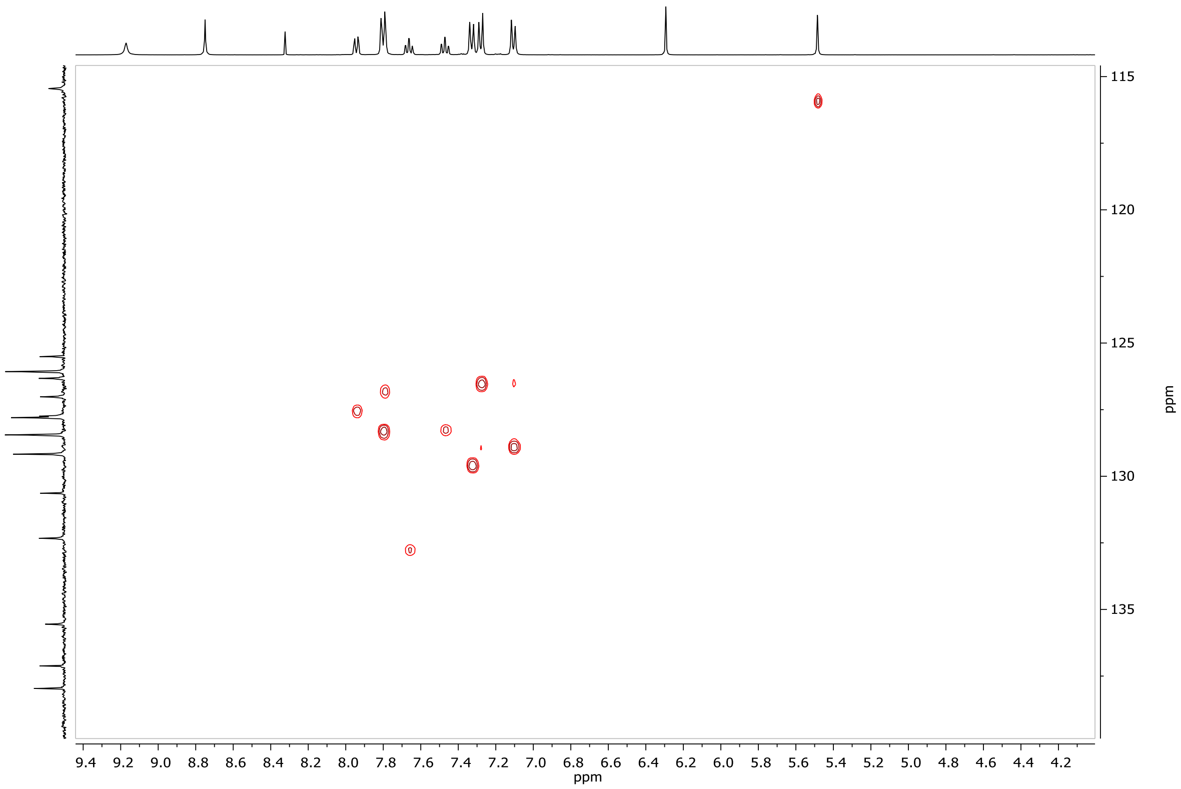

HMBC $^{1}H$-$^{13}C$ NMR (DMSO-$d_6$) spectrum of compound **18**

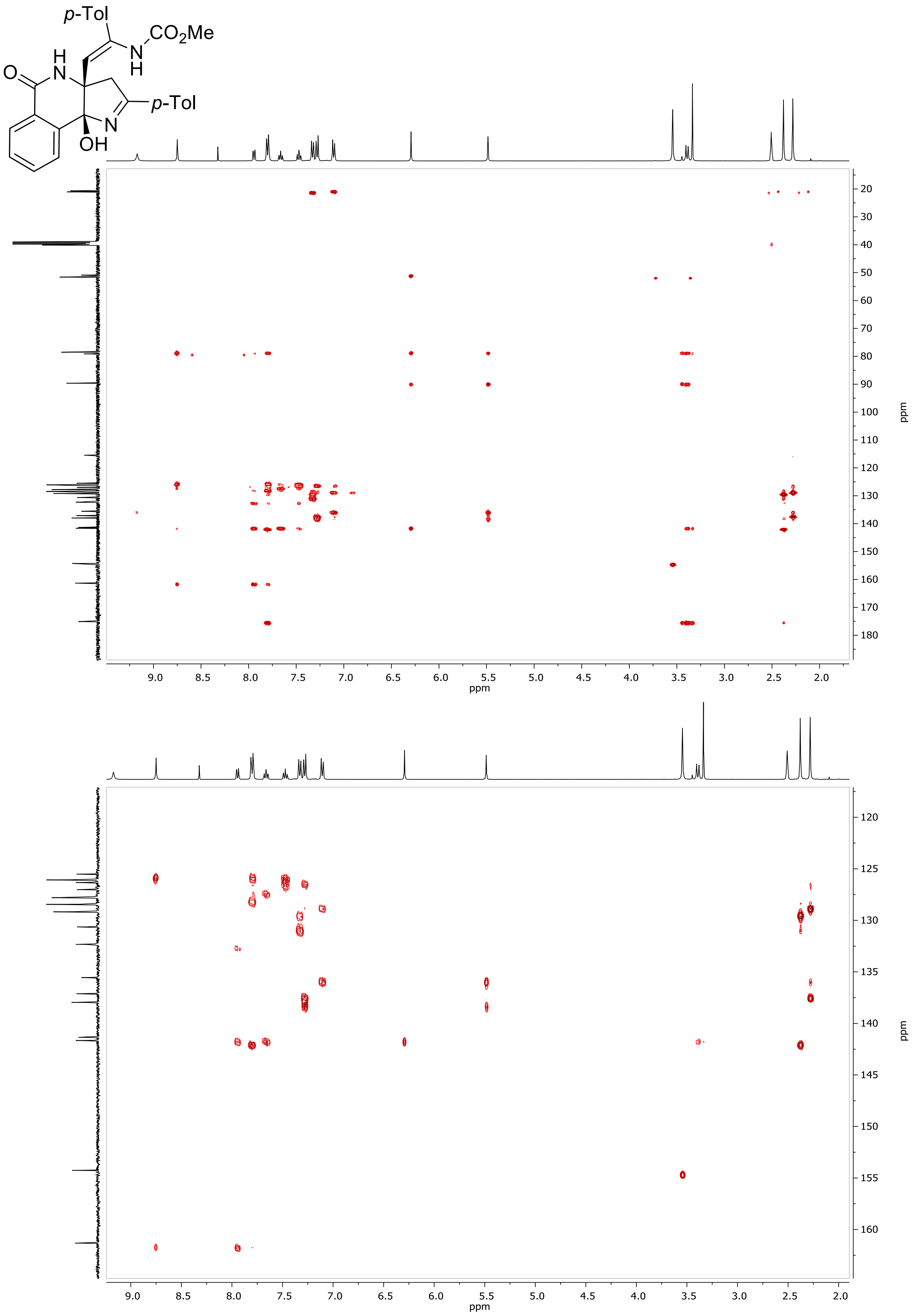

HMBC $^1H$-$^{15}N$ (DMSO-$d_6$) spectrum of compound **18**

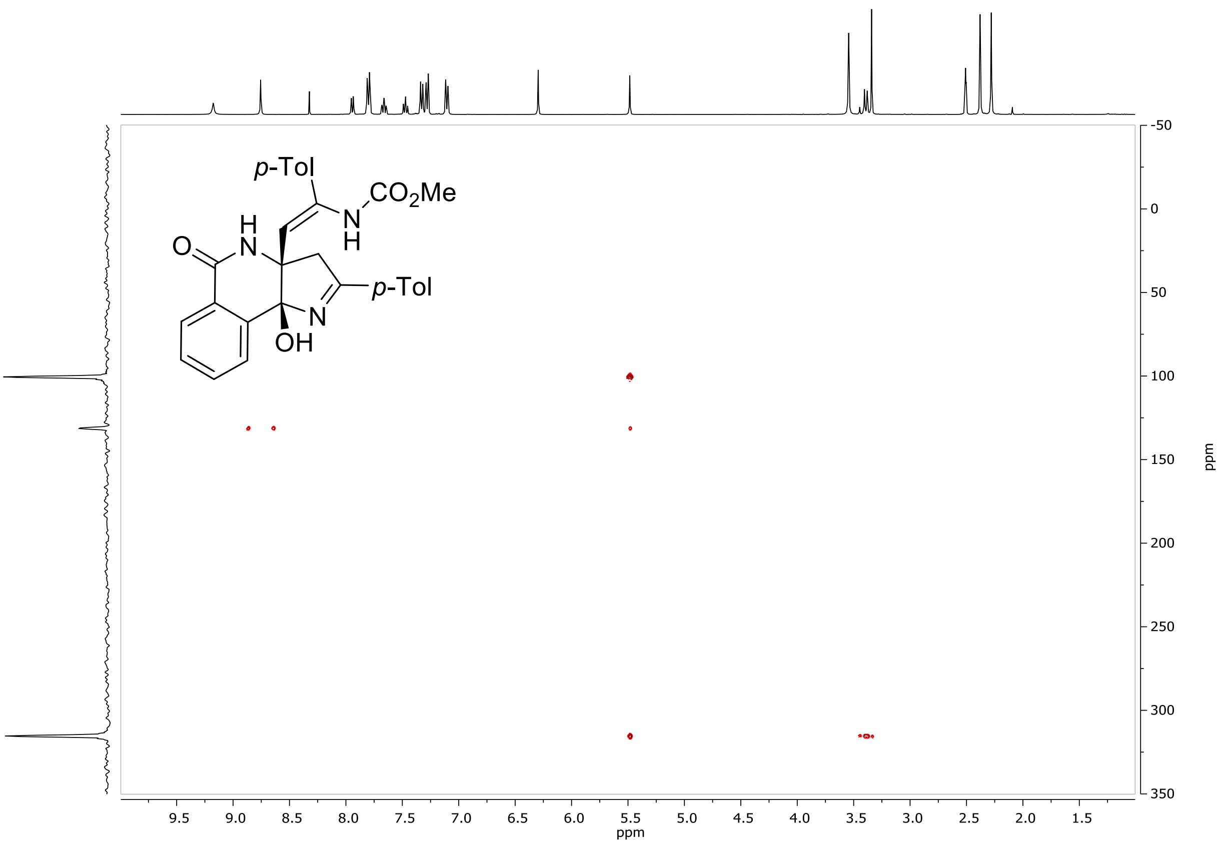


HSQC $^1H$-$^{15}N$ NMR (DMSO-$d_6$) spectrum of compound **18**

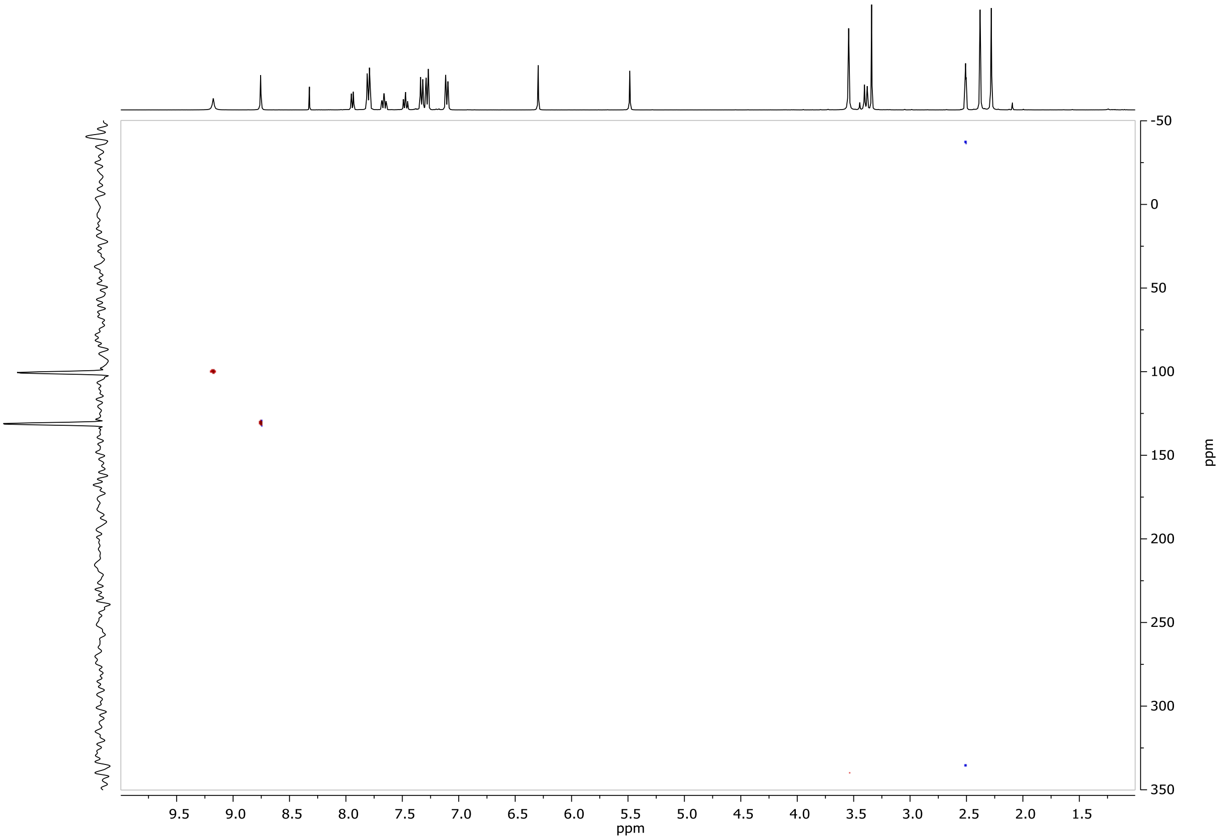

## 2. X-ray Data

### Crystallographic data of compound 3e (CCDC 1535459)

Single crystal of compound **3e** was grown by slow evaporation of hexane-diethyl ether solution. A suitable crystal was selected and studied on a Agilent Technologies Xcalibur Eos diffractometer. The crystal was kept at 100(2) K during data collection. Using Olex2 [1], the structure was solved with the ShelXS [2] structure solution program using Direct Methods and refined with the ShelXL [3] refinement package using Least Squares minimization.

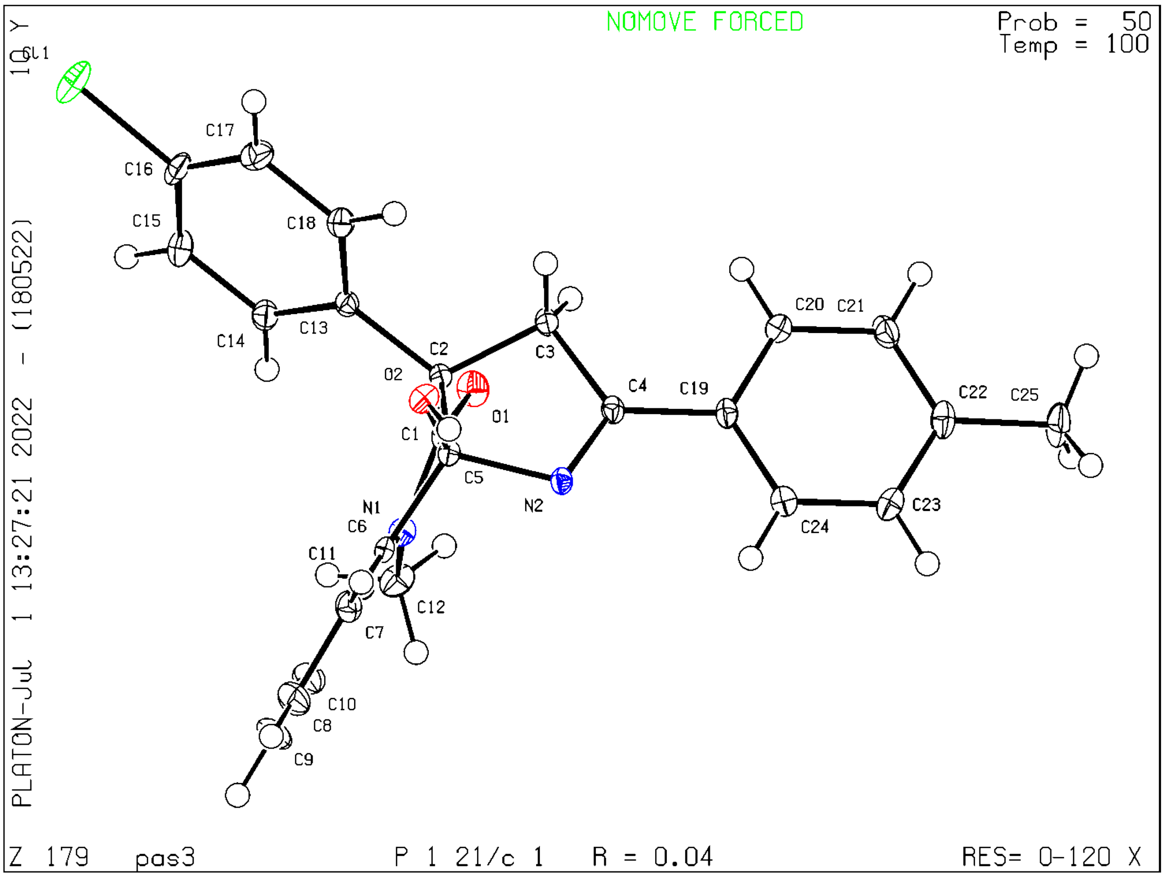


**Figure S1.** Molecular structure of compound **3e**, displacement parameters are drawn at 50% probability level.

**Table S1 Crystal data and structure refinement for 3e.**

| | |
|---|---|
| Empirical formula | $C_{25}H_{21}N_2O_2Cl$ |
| Formula weight | 416.89 |
| Temperature/K | 100(2) |
| Crystal system | monoclinic |
| Space group | $P2_1/c$ |
| a/Å | 12.0877(6) |
| b/Å | 9.3537(4) |
| c/Å | 18.5922(10) |
| α/° | 90 |
| β/° | 105.038(5) |
| γ/° | 90 |
| Volume/Å$^3$ | 2030.15(18) |
| Z | 4 |
| $\rho_{calc}$g/cm$^3$ | 1.364 |
| μ/mm$^{-1}$ | 0.213 |
| F(000) | 872.0 |
| Crystal size/mm$^3$ | 0.34 × 0.18 × 0.14 |
| Radiation | MoKα (λ = 0.71073) |
| 2Θ range for data collection/° | 6.29 to 55 |
| Index ranges | $-11 \leq h \leq 15$, $-11 \leq k \leq 12$, $-21 \leq l \leq 24$ |
| Reflections collected | 11952 |
| Independent reflections | 4647 [$R_{int}$ = 0.0263, $R_{sigma}$ = 0.0345] |
| Data/restraints/parameters | 4647/0/274 |
| Goodness-of-fit on $F^2$ | 1.020 |
| Final R indexes [I>=2σ (I)] | $R_1$ = 0.0373, $wR_2$ = 0.0888 |
| Final R indexes [all data] | $R_1$ = 0.0476, $wR_2$ = 0.0957 |
| Largest diff. peak/hole / e Å$^{-3}$ | 0.36/-0.30 |

## Crystallographic data of compound 6c (CCDC 2182542)

Single crystal of compound **6c** was grown by slow evaporation of EtOAc-hexane solution. A suitable crystal was selected and studied on an Agilent Technologies Supernova diffractometer. The crystal was kept at 100(2) K during data collection. Using Olex2 [1], the structure was solved with the ShelXT [4] structure solution program using Intrinsic Phasing and refined with the ShelXL [3] refinement package using Least Squares minimization.

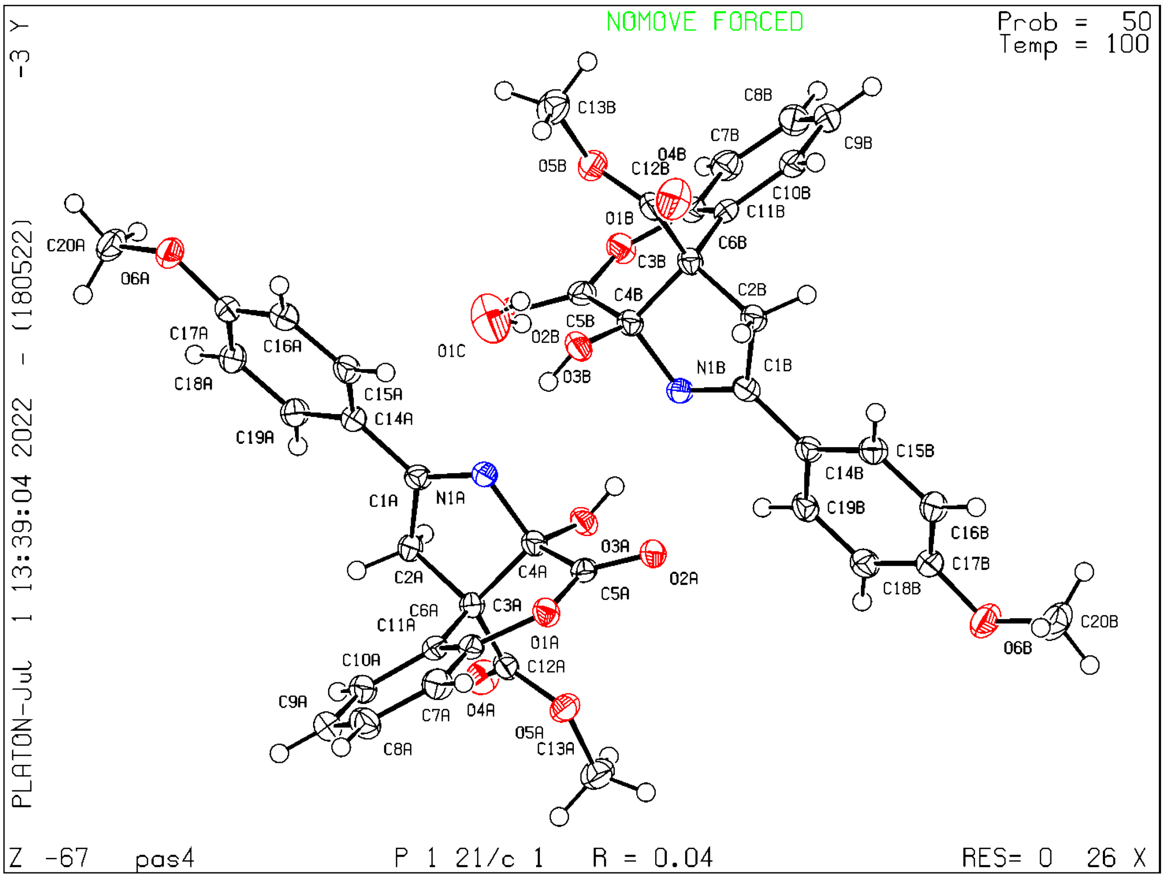


**Figure S2.** Molecular structure of compound **6c**, displacement parameters are drawn at 50% probability level.

**Table S2 Crystal data and structure refinement for 6c.**

| | |
|---|---|
| Empirical formula | $C_{20}H_{17.46}NO_{6.23}$ |
| Formula weight | 371.45 |
| Temperature/K | 100.01(10) |
| Crystal system | monoclinic |
| Space group | $P2_1/c$ |
| a/Å | 18.9991(6) |
| b/Å | 11.0188(2) |
| c/Å | 17.6331(7) |
| α/° | 90 |
| β/° | 107.678(4) |
| γ/° | 90 |
| Volume/Å$^3$ | 3517.1(2) |
| Z | 8 |
| $\rho_{calc}$g/cm$^3$ | 1.403 |
| μ/mm$^{-1}$ | 0.882 |
| F(000) | 1554.0 |
| Crystal size/mm$^3$ | 0.45 × 0.34 × 0.26 |
| Radiation | Cu Kα (λ = 1.54184) |
| 2Θ range for data collection/° | 9.396 to 144.988 |
| Index ranges | $-23 \leq h \leq 22$, $-13 \leq k \leq 5$, $-20 \leq l \leq 21$ |
| Reflections collected | 12891 |
| Independent reflections | 6683 [$R_{int}$ = 0.0310, $R_{sigma}$ = 0.0355] |
| Data/restraints/parameters | 6683/0/505 |
| Goodness-of-fit on F$^2$ | 1.077 |
| Final R indexes [I>=2σ (I)] | $R_1$ = 0.0409, $wR_2$ = 0.1090 |
| Final R indexes [all data] | $R_1$ = 0.0591, $wR_2$ = 0.1335 |
| Largest diff. peak/hole / e Å$^{-3}$ | 0.34/-0.30 |

## 3. References


1. Dolomanov, O.V.; Bourhis, L.J.; Gildea, R.J; Howard, J.A.K.; Puschmann, H. OLEX2: a complete structure solution, refinement and analysis program. *J. Appl. Cryst*. **2009**, *42*, 339–341.
2. Sheldrick, G.M. A short history of SHELX. *Acta Cryst*. 2008, A64, 112-122.
3. Sheldrick, G.M. Crystal Structure Refinement with SHELXL. *Acta Cryst.* **2015**, C71, 3–8.
4. Sheldrick, G.M. SHELXT – Integrated Space-Group and Crystal-Structure Determination. *Acta Cryst*. **2015**, A71, 3–8.
